# Intermolecular Aryl C−H Amination through Sequential Iron and Copper Catalysis

**DOI:** 10.1002/chem.201605671

**Published:** 2016-12-16

**Authors:** Mohamed A. B. Mostafa, Ewen D. D. Calder, Daugirdas T. Racys, Andrew Sutherland

**Affiliations:** ^1^ WestCHEM, School of Chemistry, The Joseph Black BuildingUniversity of GlasgowUniversity AvenueGlasgowG12 8QQUK

**Keywords:** amination, bromination, copper catalysis, cross-coupling, iron catalysis

## Abstract

A mild, efficient and regioselective method for *para*‐amination of activated arenes has been developed through a combination of iron and copper catalysis. A diverse range of products were obtained from an operationally simple one‐pot, two‐step procedure involving bromination of the aryl substrate with the powerful Lewis acid iron(III) triflimide, followed by a copper(I)‐catalysed *N*‐arylation reaction. This two‐step dehydrogenative process for the regioselective coupling of aromatic C−H bonds with non‐activated amines was applicable to anisole‐, phenol‐, aniline‐ and acetanilide‐type aryl compounds. Importantly, the arene substrates were used as the limiting reagent and required no protecting‐group manipulations during the transformation.

The ability to efficiently and selectively form aryl C−N bonds is an important challenge in organic chemistry owing to the widespread occurrence of this key linkage in natural products, pharmaceuticals, agrochemicals and organic materials.^1^ Historically, aryl amination was conducted by nitration of the aromatic ring, followed by reduction but the use of strongly acidic conditions has limited the general application of this method.^2^ Another approach is the copper‐ or palladium‐catalysed amination of aryl (pseudo)halides through Ullmann–Goldberg, Chan–Evans–Lam or Buchwald–Hartwig protocols.^3^, ^4^ Although this method typically produces a single regioisomer, prefunctionalisation of the aromatic ring is required.

To circumvent prefunctionalisation or preoxidation of either partner, new methods for direct intermolecular dehydrogenative coupling of aryl C−H bonds with non‐activated amines have recently been reported.^5^ For example, highly regioselective intermolecular *ortho*‐amination through transition‐metal‐catalysed chelation‐directed aryl C−H activation under oxidative conditions has been developed for efficient C−N bond formation (Figure 1 a).^6^, ^7^ Procedures for directed aryl C−H and N−H bond coupling at more distal positions are less well established. Only recently has a directed *meta*‐amination process been reported^8^ and only a few examples are known for directed *para*‐amination utilising transition‐metal‐, organo‐ or photocatalysis, as well as non‐catalytic oxidative methods.^9^ Many of these tend to use the aryl compound as the solvent and/or generate mixtures of regioisomers. Exceptions that overcome these issues are known^10^ and include work by Suna and co‐workers who have used the electrophilic reaction of arenes with hypervalent iodonium reagents to form unsymmetrical diaryl‐λ^3^‐iodane intermediates that are then subjected to a copper(I)‐catalysed amination (Figure 1 b).^11^ We were interested in developing a catalytic method for amination of aryl C−H bonds that utilised only readily available, inexpensive, nontoxic first‐row transition metals. Here, we now report a one‐pot, two‐step method for the coupling of aryl C−H bonds with N−H bonds by using a combination of iron(III)‐ and copper(I)‐catalysis (Figure 1 c). This *para*‐directed process is complementary to *ortho*‐directed methods and is applicable for the general coupling of aryl compounds with N‐heterocycles, amides and sulfonamides.

**Figure 1:**
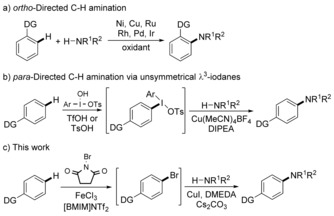
Strategies for regioselective aryl C−H amination. DIPEA=*N*,*N*‐diisopropylethylamine; DMEDA=*N*,*N*′‐dimethylethylenediamine.

Before developing the one‐pot *para*‐amination process, the scope, efficiency and regioselectivity of the iron(III)‐catalysed aryl bromination reaction was initially assessed. We recently demonstrated that metal triflimide catalysis could be used to activate *N*‐iodosuccinimide (NIS) for regioselective iodination of arenes and so a similar procedure was used to investigate bromination (Scheme 1).^12^, ^13^ A mixture of iron(III) chloride (5 mol %) with the inexpensive ionic liquid 1‐butyl‐3‐methylimidazolium bis(trifluoromethylsulfonyl)imide ([BMIM]NTf_2_), which forms the powerful Lewis acid Fe(NTf_2_)_3_ in situ,^14^ was found to activate *N*‐bromosuccinimide (NBS) for the rapid and efficient bromination of a wide range of anisoles, phenols, anilines and acetanilides under mild conditions.^15^ Arenes with strongly deactivating groups (e.g., **1 l** and **1 m**), naphthalenes (**1 q** and **1 r**) and 2,3‐benzodihydrofuran (**1 s**) were all tolerated as substrates for effective bromination. Apart from anisole (**1 a**), which yielded a 95:5 ratio of *p*‐ and *o*‐isomers, the transformation was found to generate the aryl bromides as single regioisomers. A dibromination reaction was also investigated with cyanophenol (**1 t**). The use of two equivalents of NBS resulted in clean dibromination and the efficient synthesis of bromoxynil (**2 t**), a commercially used nitrile herbicide.^16^

**Scheme 1:**
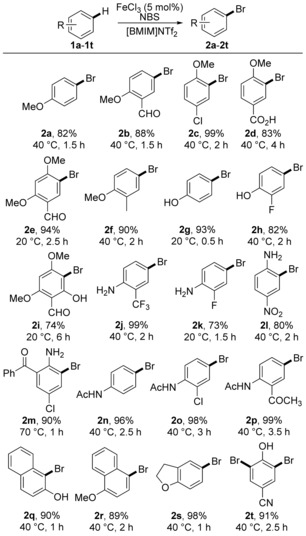
Scope of iron(III)‐catalysed bromination.

Having shown the generality of the Fe(NTf_2_)_3_‐catalysed aryl bromination, conditions for the one‐pot, two‐step amination process were then investigated (Table 1). Initially, the standard method for the bromination reaction [with FeCl_3_ (5 mol %) and the ionic liquid as the solvent] was then followed with a copper(I) iodide‐catalysed amination with indole as the nucleophile and caesium carbonate as the base (Table 1, entry 1). Although bromination was successful, no amination was observed. To allow better compatibility between the two steps, a bromination protocol with catalytic amounts of both FeCl_3_ and [BMIM]NTf_2_ in toluene was developed. Through this strategy and under typical conditions (40 °C, 4 h), complete bromination was observed and, when combined with the copper(I)‐catalysed amination, conversion to the coupled product **3 a** was observed (14 %, entry 2). A further reduction of the catalyst loading in either DMF or toluene resulted in improved overall conversions (entries 3 and 4). However, a significant breakthrough was achieved when water was added as a co‐solvent to the second step. This allowed dissolution of the base and better mixing of reagents, with the result of nearly quantitative conversion to **3 a** (entry 5). Further optimisation studies showed that the catalyst loading of both FeCl_3_ (2.5 mol %) and [BMIM]NTf_2_ (7.5 mol %) could be lowered further without affecting the rate or overall conversion of the one‐pot process (entry 6).

**Table 1:** Optimisation of the one‐pot amination process.^[a]^

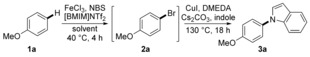				
Entry	FeCl_3_ [mol %]	[BMIM]NTf_2_ [mol %]	Solvent	**3 a** Conv.^[b]^ [%]
1	5	–	[BMIM]NTf_2_	0
2	10	30	toluene	14
3	5	15	DMF	39
4	5	15	toluene	45
5	5	15	toluene^[c]^	>95
6	2.5	7.5	toluene^[c]^	>95

[a] Copper(I) iodide (10 mol %) and DMEDA (20 mol %) were used in the amination reactions; [b] determined by ^1^H NMR analysis of the crude reaction mixtures; [c] water (40 % of reaction volume) was added to the second step.

Following optimisation studies, the scope of the one‐pot amination process of anisole (**1 a**) with various N‐nucleophiles was explored (Scheme 2). With a range of N‐heterocycles such as indole, pyrazole, imidazole, pyrrole and pyrrolidin‐2‐one as well as amides and sulfonamides, the one‐pot process was found to generate the coupled products cleanly and in good yields as single regioisomers. The use of this procedure for multigram synthesis of aminated aryl compounds was also demonstrated with the large‐scale synthesis of pyrazole **3 b** in essentially quantitative yield.

**Scheme 2:**
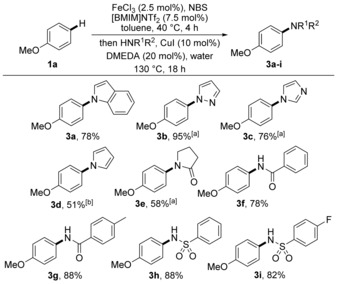
Scope of the one‐pot amination process with anisole (**1 a**). [a] Amination was performed at 150 °C for 24 h; [b] amination was performed at 150 °C for 36 h.

With pyrazole and benzamide as nucleophiles, the scope of the aryl coupling partner was then explored (Scheme 3). The one‐pot process was found to afford *para*‐aminated products in good yields for various anisole‐, phenol‐, aniline‐ and acetanilide‐based coupling partners, in the presence of either activating or deactivating substituents. In addition, despite the use of aryl compounds containing nucleophilic functional groups (e.g., phenols, anilines), the one‐pot transformation afforded the *N*‐coupled products cleanly.

**Scheme 3:**
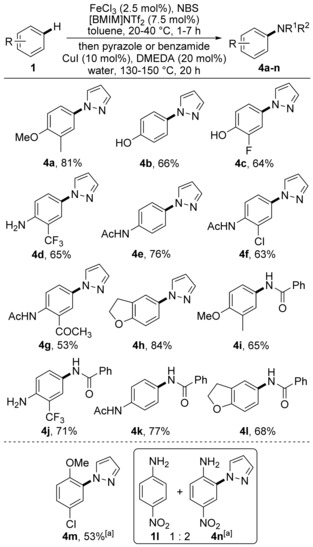
Scope and limitations of the aryl coupling partner. [a] Brominations were performed at 70 °C with FeCl_3_ (5 mol %) and [BMIM]NTf_2_ (15 mol %).

As part of this research program, a preliminary investigation into *ortho*‐*N*‐arylation by using substrates with the *para*‐position blocked was also conducted. The one‐pot bromination and amination of *p*‐chloroanisole (**1 c**) with pyrazole required more forcing conditions but afforded **4 m** cleanly in 53 % yield (Scheme 3). However, a limitation of this approach was demonstrated in a similar one‐pot process with *p*‐nitroaniline (**1 l**), which yielded an inseparable mixture of **1 l** and the coupled product **4 n**.^17^ In this case, slower copper‐catalysed *ortho*‐amination results in a competing pathway involving reduction of the organocopper intermediate, as evidenced by the regeneration of **1 l** from the bromide intermediate.^18^

Methods for the one‐step amination of aromatic halides involving iron and copper catalysis have been previously reported. Correa and Bolm reported an iron‐catalysed *N*‐arylation reaction that was later found to be catalysed by copper contaminants,^19^ whereas Taillefer et al. described an iron–copper co‐catalysed process.^20^ To investigate the individual role of each metal catalyst during the amination step of this current process, a series of experiments was performed to mimic the second step of the one‐pot process (Table 2). As expected, these results confirmed that copper iodide is essential for efficient conversion to the *N*‐coupled product. Although this does not rule out an iron–copper co‐catalysed amination, the Taillefer study does emphasise that pre‐complexation of iron(III) with the acetylacetonate ligand was critical for effective co‐catalysis (use of FeCl_3_ as a co‐catalyst afforded low yields).^20^

**Table 2:** Role of iron and copper catalysts during the amination step.^[a]^

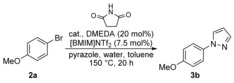		
Entry	Catalyst	Yield [%]
1	FeCl_3_	<5
2	CuI	92
3	FeCl_3_/CuI	87

[a] Standard catalyst loadings were used (FeCl_3_: 2.5 mol %; CuI: 10 mol %).

In conclusion, a general and efficient approach for the regioselective bromination of aryl compounds has been demonstrated by using iron(III) triflimide activation of NBS. Combination of a modified version of this transformation that utilises catalytic amounts of both FeCl_3_ and [BMIM]NTf_2_ with a copper(I)‐catalysed amination allowed the development of a one‐pot, two‐step dehydrogenative process for the regioselective coupling of aromatic C−H bonds with non‐activated amines. This process was found to be general for the *para*‐amination of a range of aromatic compounds in combination with various nitrogen nucleophiles such as N‐heterocycles, amides and sulfonamides. This study provides a conceptually new, one‐pot approach for the preparation of valuable synthetic building blocks that is complementary to *ortho*‐C−H amination methods and overcomes the limitations of the multistep and harsh conditions of more traditional chemistries.

## Supporting information

As a service to our authors and readers, this journal provides supporting information supplied by the authors. Such materials are peer reviewed and may be re‐organized for online delivery, but are not copy‐edited or typeset. Technical support issues arising from supporting information (other than missing files) should be addressed to the authors.

Supplementary
