# Supplementary material for: Intermolecular Aryl C−H Amination through Sequential Iron and Copper Catalysis
Source: Chemistry. 2016 Dec 16;23(5):1044–7. doi: 10.1002/chem.201605671 (PMC5396366; doi:10.1002/chem.201605671)
Supplement: Supplementary file 1 — Supplementary [file CHEM-23-1044-s001.pdf]

CHEMISTRY
A European Journal

Supporting Information

Intermolecular Aryl C–H Amination through Sequential Iron and Copper Catalysis

Mohamed A. B. Mostafa, Ewen D. D. Calder, Daugirdas T. Racys, and Andrew Sutherland*[a]

chem_201605671_sm_miscellaneous_information.pdf

Table of Contents

1.	General Experimental	2
2.	General Procedure A: Bromination	2–3
3.	General Procedure B: One-pot Bromination/*N*-Arylation Reaction	3
4.	Experimental Procedures and Spectroscopic Data for All Compounds	3–23
5.	References	23–24
6.	^{1}H NMR and ^{13}C NMR Spectra of All Compounds	25–108

1. General Experimental

All reagents and starting materials were obtained from commercial sources and used as received. All dry solvents were purified using a solvent purification system. All reactions were performed open to air unless otherwise mentioned. Brine refers to a saturated solution of sodium chloride. Flash column chromatography was performed using silica gel 60 (35–70 μm). Aluminium-backed plates pre-coated with silica gel $60F_{254}$ were used for thin layer chromatography and were visualized with a UV lamp or by staining with potassium permanganate. ^{1}H NMR spectra were recorded on a NMR spectrometer at either 400 or 500 MHz and data are reported as follows: chemical shift in ppm relative to tetramethylsilane as the internal standard, multiplicity (s = singlet, d = doublet, t = triplet, q = quartet, m = multiplet or overlap of nonequivalent resonances, integration). ^{13}C NMR spectra were recorded on a NMR spectrometer at either 101 or 126 MHz and data are reported as follows: chemical shift in ppm relative to tetramethylsilane or the solvent as internal standard ($CDCl_3$, δ 77.0 ppm), multiplicity with respect to hydrogen (deduced from DEPT experiments, C, CH, CH_2 or CH_3). Infrared spectra were recorded on a FTIR spectrometer; wavenumbers are indicated in cm^{-1}. Mass spectra were recorded using electron impact or electrospray techniques. HRMS spectra were recorded using a dual-focusing magnetic analyzer mass spectrometer. Melting points are uncorrected.

2. General Procedure A: Bromination

N-Bromosuccinimide (1.0 mmol) was added to a solution of iron(III) chloride (0.05 mmol) in 1-butyl-3-methylimidazolium bis(trifluoromethanesulfonyl)imide ([BMIM]NTf_2) (0.30 mL) under an atmosphere of air. The mixture was stirred at room temperature for 0.5 h before the substrate (1.0 mmol) in [BMIM]NTf_2 (0.10 mL) was added. The reaction mixture was stirred at the required temperature. The reaction progress was monitored by ^{1}H NMR spectroscopy. The reaction mixture was extracted into 5% ethyl acetate/hexane (3 × 10 mL) using sonication in a water bath for 0.1 h. The suspension was washed with an aqueous solution of 1 M sodium thiosulfate (10 mL) and brine (10 mL), dried over $MgSO_4$ and then filtered through a pad of Celite®. The solvent was removed under reduced pressure and the crude product was purified by flash column chromatography. **Work Up for Carboxylic acids and phenol derivatives.** The reaction mixture was diluted with dichloromethane (20 mL) and extracted with an aqueous solution of 1 M sodium hydroxide. The aqueous phase was separated and then acidified with 1 M hydrochloric acid, and extracted into dichloromethane (2 × 50 mL).

The organic phase was dried over $MgSO_4$ and the solvent was removed under reduced pressure. The crude product was purified by flash column chromatography.

3. General Procedure B: One-pot Bromination/*N*-Arylation Reaction.

Iron(III) trichloride (0.025 mmol) was dissolved in [BMIM]NTf_2 (0.075 mmol) and pre-stirred for 0.5 h at room temperature and then added to a solution of *N*-bromosuccinimide (1.0 mmol) in toluene (1 mL). The substrate (1.0 mmol) was then added and the mixture was stirred at the required temperature. Upon the completion of the bromination, the reaction mixture was cooled to room temperature and the *N*-nucleophile (1.50 mmol), copper(I) iodide (0.10 mmol), cesium carbonate (2.00 mmol), *N*,*N*′-dimethylethylenediamine (0.20 mmol) and water (0.7 mL) were added. The reaction mixture was degassed under argon for 0.1 h before heated to the required temperature. The reaction mixture was then cooled to room temperature, diluted with ethyl acetate (10 mL), washed with a 10% sodium thiosulfate solution (10 mL) and brine (10 mL). The organic phase was dried ($MgSO_4$), filtered and concentrated *in vacuo*. The crude product was purified by flash column chromatography.

4. Experimental Procedures and Spectroscopic Data for all Compounds

4-Bromoanisole (2a)[1]

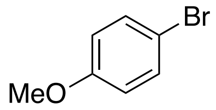

The reaction was performed as described in general procedure A using anisole (**1a**) (0.10 g, 0.93 mmol). The reaction mixture was heated to 40 °C for 1.5 h. Purification by flash column chromatography (petroleum ether/ethyl acetate, 9:1) gave 4-bromoanisole (**2a**) (0.14 g, 82%) as a pale yellow oil. Spectroscopic data was consistent with the literature.[1] ^{1}H NMR (400 MHz, $CDCl_3$) δ 3.78 (s, 3H), 6.76–6.81 (m, 2H), 7.35–7.40 (m, 2H); ^{13}C NMR (101 MHz, $CDCl_3$) δ 55.4 (CH_3), 112.8 (C), 115.8 (2 × CH), 132.3 (2 × CH), 158.7 (C); MS (EI) *m/z* 186 (M^+. 100), 171 (35), 143 (28).

5-Bromo-2-methoxybenzaldehyde (2b)[2]

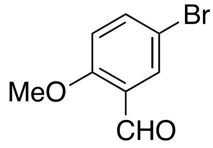

The reaction was performed as described in general procedure A using 2-methoxybenzaldehyde (**1b**) (60 mg, 0.44 mmol). The reaction mixture was heated to 40 °C for 1.5 h. Purification by flash column chromatography (petroleum ether/ethyl acetate, 9:1) gave 5-bromo-2-methoxybenzaldehyde (**2b**) (83 mg, 88%) as a white solid. Mp 112–114 °C (lit.[2] 116–117 °C); ^{1}H NMR (400 MHz, $CDCl_3$) δ 3.93 (s, 3H), 6.90 (d, J = 8.9 Hz, 1H), 7.64 (dd, J = 8.9, 2.6 Hz, 1H), 7.92 (d, J = 2.6 Hz, 1H), 10.39 (s, 1H); ^{13}C NMR (101 MHz, $CDCl_3$) δ 56.0 (CH_3), 113.5 (C), 113.7 (CH), 126.1 (C), 131.1 (CH), 138.3 (CH), 160.7 (C), 188.3 (CH); MS (EI) *m/z* 214 (M^+. 100), 170 (30), 143 (20), 135 (8), 118 (24), 92 (10), 75 (27), 63 (30), 50 (12).

2-Bromo-4-chloroanisole (2c)[3]

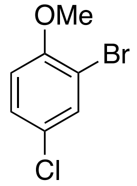

The reaction was performed as described in general procedure A using 4-chloroanisole (**1c**) (62 µL, 0.50 mmol). The reaction mixture was heated to 40 °C for 1 h. Purification by flash column chromatography (petroleum ether/ethyl acetate, 17:3) gave 2-bromo-4-chloroanisole (**2c**) (109 mg, 99%) as a colorless oil. Spectroscopic data was consistent with the literature.[3] ^{1}H NMR (500 MHz, $CDCl_3$) δ 3.88 (s, 3H), 6.82 (d, J = 8.8 Hz, 1H), 7.24 (dd, J = 8.8, 2.5 Hz, 1H), 7.54 (d, J = 2.5 Hz, 1H); ^{13}C NMR (126 MHz, $CDCl_3$) δ 56.6 (CH_3), 112.3 (C), 112.7 (CH), 126.1 (C), 128.4 (CH), 132.9 (CH), 154.9 (C); MS (EI) *m/z* 222 (M^+. 100), 207 (50), 179 (36), 126 (10), 75 (12), 63 (21).

3-Bromo-4-methoxybenzoic acid (2d)[4]

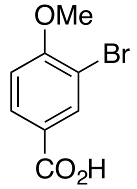

The reaction was performed as described in general procedure A using 4-methoxybenzoic acid (**1d**) (76 mg, 0.50 mmol). The reaction mixture was heated to 40 °C for 4 h. Purification by flash column chromatography (dichloromethane/methanol, 19:1) gave 3-bromo-4-methoxybenzoic acid (**2d**) (96 mg, 83%) as a white solid. Mp 206–208 °C (lit.[4] 201–206 °C); ^{1}H NMR (500 MHz, $CDCl_3$) δ 3.98 (s, 3H), 6.95 (d, J = 8.7 Hz, 1H), 8.06 (dd, J = 8.7, 2.1 Hz, 1H), 8.30 (d, J = 2.1 Hz, 1H); ^{13}C NMR (126 MHz, $CDCl_3$) δ 56.5 (CH_3), 111.1 (CH), 111.6 (C), 122.7 (C), 131.4 (CH), 135.5 (CH), 160.2 (C), 169.8 (C); MS (ESI) *m/z* 255 (MNa^+. 100).

5-Bromo-2,4-dimethoxybenzaldehyde (2e)[5]

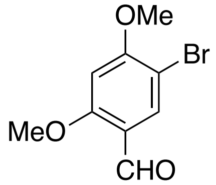

The reaction was performed as described in general procedure A using 2,4-dimethoxybenzaldehyde (**1e**) (0.080 g, 0.50 mmol). The reaction mixture was stirred at 20 °C for 2.5 h. Purification by flash column chromatography (petroleum ether/ethyl acetate, 5:1) gave 5-bromo-2,4-dimethoxybenzaldehyde (**2e**) (0.12 g, 94%) as a white solid. Mp 134–136 °C (lit.[5] 139–140 °C); ^{1}H NMR (500 MHz, $CDCl_3$) δ 3.93 (s, 3H), 3.96 (s, 3H), 6.42 (s, 1H), 7.96 (s, 1H), 10.20 (s, 1H); ^{13}C NMR (126 MHz, $CDCl_3$) δ 56.0 (CH_3), 56.5 (CH_3), 95.6 (CH), 103.5 (C), 119.4 (C), 132.8 (CH), 161.7 (C), 163.0 (C), 187.1 (CH); MS (ESI) *m/z* 267 (MNa^+. 100).

4-Bromo-2-methylanisole (2f)[6]

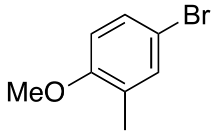

The reaction was performed as described in general procedure A using 2-methylanisole (**1f**) (62 μL, 0.50 mmol). The reaction mixture was heated to 40 °C for 2 h. Purification by flash column chromatography (petroleum ether/ethyl acetate, 9:1) gave 4-bromo-2-methylanisole (**2f**) (90 mg, 90%) as a light yellow solid. Mp 64–66 °C (lit.[6] 66–68 °C); ^{1}H NMR (400 MHz, $CDCl_3$) δ 2.21 (s, 3H), 3.82 (s, 3H), 6.70 (d, J = 9.0 Hz, 1H), 7.24–7.30 (m, 2H); ^{13}C NMR (126 MHz, $CDCl_3$) δ 16.0 (CH_3), 55.5 (CH_3), 111.5 (CH), 112.4 (C), 129.0 (C), 129.4 (CH), 133.2 (CH), 156.9 (C); MS (EI) *m/z* 200 (M^{+}. 100), 185 (47), 149 (27), 111 (36), 97 (49), 85 (54), 78 (35), 71 (69), 57 (100).

4-Bromophenol (2g)[7]

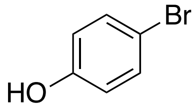

The reaction was performed as described in general procedure A using phenol (**1g**) (47 mg, 0.50 mmol). The reaction mixture was stirred at 20 °C for 0.5 h. Purification by flash column chromatography (petroleum ether/ethyl acetate, 9:1) gave 4-bromophenol (**2g**) (82 mg, 93%) as a white solid. Mp 58–60 °C (lit.[7] 56–59 °C); ^{1}H NMR (400 MHz, $CDCl_3$) δ 5.03 (br s, 1H), 6.70–6.75 (m, 2H), 7.31–7.36 (m, 2H); ^{13}C NMR (101 MHz, $CDCl_3$) δ 113.0 (C), 117.2 (2 × CH), 132.5 (2 × CH), 154.6 (C); MS (ESI) *m/z* 173 ($[M–H]^{-}$. 100).

4-Bromo-2-fluorophenol (2h)[8]

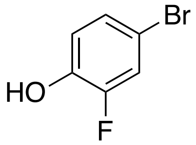

The reaction was performed as described in general procedure A using 2-fluorophenol (**1h**) (44 μL, 0.50 mmol). The reaction mixture was heated to 40 °C for 2 h. Purification by flash column chromatography (petroleum ether/ethyl acetate, 9:1) gave 4-bromo-2-fluorophenol (**2g**) (77 mg, 82%) as a colorless oil. Spectroscopic data was consistent with the literature.[8] ^{1}H NMR (500 MHz, $CDCl_3$) δ 5.07 (d, J = 4.2 Hz, 1H), 6.90 (dd, J = 9.0, 8.8 Hz, 1H), 7.16 (ddd, J = 8.8, 2.2, 1.6 Hz, 1H), 7.24 (dd, J = 9.9, 2.2 Hz, 1H); ^{13}C NMR (126 MHz, $CDCl_3$) δ

111.7 (C, d, ${}^{3}J_{CF}$ = 8.2 Hz), 118.7 (CH, d, ${}^{4}J_{CF}$ = 2.2 Hz), 119.2 (CH, d, ${}^{2}J_{CF}$ = 21.1 Hz), 128.1 (CH, d, ${}^{3}J_{CF}$ = 3.8 Hz), 143.0 (C, d, ${}^{2}J_{CF}$ = 14.1 Hz), 151.0 (C, d, ${}^{1}J_{CF}$ = 241.8 Hz); MS (EI) *m/z* 190 (M^{+}. 100), 161 (4), 142 (9), 111 (6), 83 (29), 63 (38), 57 (19).

5-Bromo-2,4-dimethoxy-6-hydroxybenzaldehyde (2i)

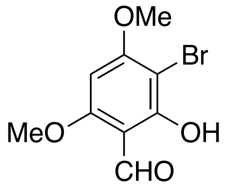

The reaction was performed as described in general procedure A using 2,4-dimethoxy-6-hydroxybenzaldehyde (**1i**) (92 mg, 0.50 mmol). The reaction mixture was stirred at 20 °C for 6 h. Purification by flash column chromatography (petroleum ether/ethyl acetate, 7:3) gave 5-bromo-2,4-dimethoxy-6-hydroxybenzaldehyde (**2i**) (97 mg, 74%) as a white solid. Mp 188–190 °C; IR (neat) 2924, 1636, 1615, 1470, 1450, 1427, 1406, 1283, 1233, 1217, 1124, 1089, 981, 772, 723 cm^{-1}; ${}^{1}H$ NMR (400 MHz, $CDCl_3$) δ 3.93 (s, 3H), 3.98 (s, 3H), 6.01 (s, 1H), 10.08 (s, 1H), 12.96 (s, 1H); ${}^{13}C$ NMR (101 MHz, $CDCl_3$) δ 56.0 (CH_3), 56.6 (CH_3), 86.9 (CH), 90.8 (C), 106.4 (C), 161.4 (C), 163.4 (C), 163.8 (C), 191.8 (C); MS (ESI) *m/z* 283 (MNa^{+}. 99); HRMS (ESI) calcd for $C_9H_9{}^{79}BrNaO_4$ (MNa^{+}), 282.9576, found 282.9575.

4-Bromo-2-trifluoromethylaniline (2j)[9]

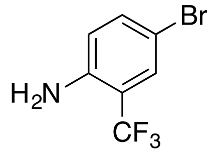

The reaction was performed as described in general procedure A using 2-trifluoromethylaniline (**1j**) (63 μL, 0.50 mmol). The reaction mixture was heated to 40 °C for 2 h. Purification by flash column chromatography (petroleum ether/ethyl acetate, 7:3) gave 4-bromo-2-trifluoromethylaniline (**2j**) (0.12 g, 99%) as a colorless oil. Spectroscopic data was consistent with the literature.[9] ${}^{1}H$ NMR (500 MHz, $CDCl_3$) δ 4.18 (br s, 2H), 6.62 (d, J = 8.7 Hz, 1H), 7.37 (dd, J = 8.7, 2.2 Hz, 1H), 7.53 (d, J = 2.2 Hz, 1H); ${}^{13}C$ NMR (126 MHz, $CDCl_3$) δ 109.1 (C), 115.3 (C, q, ${}^{2}J_{CF}$ = 30.7 Hz), 118.9 (CH), 124.1 (C, q, ${}^{1}J_{CF}$ = 272.6 Hz), 129.3 (CH, q, ${}^{3}J_{CF}$ = 5.4 Hz), 135.8 (CH), 143.6 (C); MS (EI) *m/z* 239 (M^{+}. 100), 219 (51), 192 (37), 160 (8), 140 (12), 132 (11), 113 (15), 70 (14), 63 (19).

4-Bromo-2-fluoroaniline (2k)[10]

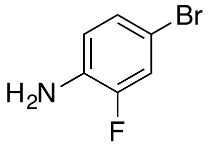

The reaction was performed as described in general procedure A using 2-fluoroaniline (**1k**) (56 mg, 0.50 mmol). The reaction mixture was stirred at 20 °C for 1.5 h. Purification by flash column chromatography (petroleum ether/ethyl acetate, 19:1) gave 4-bromo-2-fluoroaniline (**2k**) (70 mg, 73%) as a light brown oil. Spectroscopic data was consistent with the literature.[10] ^{1}H NMR (500 MHz, $CDCl_3$) δ 3.72 (br s, 2H), 6.65 (dd, J = 9.4, 8.5 Hz, 1H), 7.05 (ddd, J = 8.5, 2.2, 1.1 Hz, 1H), 7.14 (dd, J = 10.5, 2.2 Hz, 1H); ^{13}C NMR (126 MHz, $CDCl_3$) δ 108.9 (C, d, $^3J_{CF}$ = 8.8 Hz), 117.8 (CH, d, $^3J_{CF}$ = 4.3 Hz), 118.7 (CH, d, $^2J_{CF}$ = 21.9 Hz), 127.4 (CH, d, $^4J_{CF}$ = 3.6 Hz), 133.8 (C, d, $^2J_{CF}$ = 12.8 Hz), 151.4 (C, d, $^1J_{CF}$ = 243.4 Hz); MS (EI) *m/z* 189 (M^+. 100), 110 (21), 83 (27), 63 (11).

2-Bromo-4-nitroaniline (2l)[11]

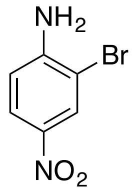

The reaction was performed as described in general procedure A using 4-nitroaniline (**1l**) (69 mg, 0.50 mmol). The reaction mixture was heated to 40 °C for 2 h. Purification by flash column chromatography (petroleum ether/ethyl acetate, 7:3) gave 2-bromo-4-nitroaniline (**2l**) (87 mg, 80%) as a yellow solid. Mp 104–106 °C (lit.[11] 103–104 °C); ^{1}H NMR (500 MHz, $CDCl_3$) δ 4.89 (br s, 2H), 6.77 (d, J = 8.9 Hz, 1H), 8.04 (dd, J = 8.9, 2.4 Hz, 1H), 8.38 (d, J = 2.4 Hz, 1H); ^{13}C NMR (126 MHz, $CDCl_3$) δ 107.1 (C), 113.6 (CH), 125.0 (CH), 129.3 (CH), 139.1 (C), 150.0 (C); MS (ESI) *m/z* 239 (MNa^+. 99), 227 (4), 200 (10).

2-Amino-3-bromo-5-chlorobenzophenone (2m)[12]

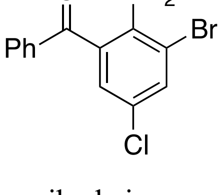

The reaction was performed as described in general procedure A using 2-amino-5-chlorobenzophenone (**1m**) (0.12 g, 0.50 mmol). The reaction mixture was heated to 70 °C for

1 h. Purification by flash column chromatography (petroleum ether/ethyl acetate, 7:3) gave 2-amino-3-bromo-5-chlorobenzophenone (**2m**) (0.14 g, 90%) as a yellow solid. Mp 101–102 °C (lit.[12] 102–103 °C); ^{1}H NMR (500 MHz, $CDCl_3$) δ 6.60 (br s, 2H), 7.41 (d, J = 2.4 Hz, 1H) 7.46–7.52 (m, 2H), 7.55–7.65 (m, 4H); ^{13}C NMR (126 MHz, $CDCl_3$) δ 111.4 (C), 119.4 (C), 119.7 (C), 128.5 (2 × CH), 129.3 (2 × CH), 132.0 (CH), 132.9 (CH), 136.6 (CH), 138.9 (C), 146.6 (C), 197.5 (C); MS (ESI) *m/z* 334 (MNa^+. 100).

4-Bromoacetanilide (2n)[13]

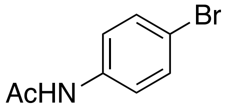

The reaction was performed as described in general procedure A using acetanilide (**1n**) (0.07 g, 0.5 mmol). The reaction mixture was heated to 40 °C for 2.5 h. Purification by flash column chromatography (petroleum ether/ethyl acetate, 7:3) gave 4-bromoacetanilide (**2n**) (0.1 g, 96%) as a white solid. Mp 162–164 °C (lit.[13] 164–166 °C); ^{1}H NMR (500 MHz, $CDCl_3$) δ 2.17 (s, 3H), 7.30 (br s, 1H), 7.37–7.44 (m, 4H); ^{13}C NMR (126 MHz, $CDCl_3$) δ 24.6 (CH_3), 116.9 (C), 121.4 (2 × CH), 132.0 (2 × CH), 136.9 (C), 168.3 (C); MS (ESI) *m/z* 236 (MNa^+. 100).

***N*-(4-Bromo-2-chlorophenyl)acetamide (2o)[14]**

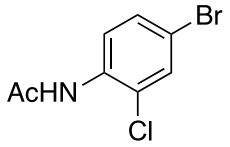

The reaction was performed as described in the general procedure A using *N*-(2-chlorophenyl)acetamide (**1o**) (0.085 g, 0.50 mmol) in a co-solvent system of [BMIM]NTf_2 (0.30 mL) and toluene (0.10 mL). The reaction mixture was heated to 40 °C for 3 h. Purification by flash column chromatography (petroleum ether/ethyl acetate, 9:1) gave *N*-(4-bromo-2-chlorophenyl)acetamide (**2o**) (0.12 g, 98%) as a white solid. Mp 148–150 °C (lit.[14] 151–152 °C); ^{1}H NMR (500 MHz, $CDCl_3$) δ 2.23 (s, 3H), 7.38 (dd, J = 8.9, 2.2 Hz, 1H), 7.51 (d, J = 2.2 Hz, 1H), 7.57 (br s, 1H), 8.28 (d, J = 8.9 Hz, 1H); ^{13}C NMR (126 MHz, $CDCl_3$) δ 24.9 (CH_3), 116.2 (C), 122.6 (CH), 123.2 (C), 130.8 (CH), 131.4 (CH), 133.8 (C), 168.2 (C); MS (ESI) *m/z* 272 (MNa^+. 100).

N-(2-Acetyl-4-bromophenyl)acetamide (2p)[15]

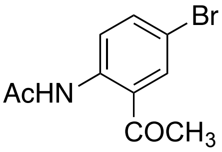

The reaction was performed as described in general procedure A using N-(2-acetylphenyl)acetamide (**1p**) (0.090 g, 0.50 mmol) in a co-solvent system of [BMIM]NTf$_2$ (0.30 mL) and toluene (0.10 mL). The reaction mixture was heated to 40 °C for 3.5 h. Purification by flash column chromatography (petroleum ether/ethyl acetate, 9:1) gave N-(2-acetyl-4-bromophenyl)acetamide (**2p**) (0.13 g, 99%) as a white solid. Mp 158–160 °C. Spectroscopic data was consistent with the literature.[15] ^{1}H NMR (500 MHz, CDCl$_3$) δ 2.21 (s, 3H), 2.64 (s, 3H), 7.61 (dd, J = 9.1, 2.3 Hz, 1H), 7.96 (d, J = 2.3 Hz, 1H), 8.66 (d, J = 9.1 Hz, 1H), 11.56 (br s, 1H); ^{13}C NMR (126 MHz, CDCl$_3$) δ 25.5 (CH$_3$), 28.6 (CH$_3$), 114.4 (C), 122.3 (CH), 123.0 (C), 134.0 (CH), 137.6 (CH), 139.9 (C), 169.4 (C), 201.6 (C); MS (ESI) m/z 278 (MNa$^+$. 97).

1-Bromo-2-naphthol (2q)[16]

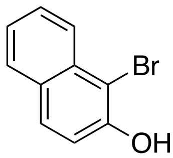

The reaction was performed as described in general procedure A using 2-naphthol (**1q**) (0.07 g, 0.5 mmol). The reaction mixture was heated to 40 °C for 1 h. Purification by flash column chromatography (petroleum ether/ethyl acetate, 19:1) gave 1-bromo-2-naphthol (**2q**) (0.1 g, 90%) as a pale green solid. Mp 72–74 °C (lit.[16] 76–78 °C); ^{1}H NMR (500 MHz, CDCl$_3$) δ 5.95 (br s, 1H), 7.28 (d, J = 8.8 Hz, 1H), 7.40 (ddd, J = 8.0, 6.9, 1.2 Hz, 1H), 7.58 (ddd, J = 8.5, 6.9, 1.2 Hz, 1H), 7.74 (d, J = 8.8 Hz, 1H), 7.79 (br d, J = 8.0 Hz, 1H), 8.04 (br d, J = 8.5 Hz, 1H); ^{13}C NMR (126 MHz, CDCl$_3$) δ 106.2 (C), 117.2 (CH), 124.2 (CH), 125.4 (CH), 127.9 (CH), 128.2 (CH), 129.4 (CH), 129.7 (C), 132.3 (C), 150.6 (C); MS (EI) m/z 222 (M$^+$. 100), 144 (12), 114 (38).

1-Bromo-4-methoxynaphthalene (2r)[17]

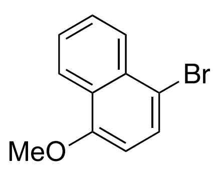

The reaction was performed as described in general procedure A using 1-methoxynaphthalene (**1r**) (0.080 g, 0.50 mmol). The reaction mixture was heated to 40 °C for 2 h. Purification by flash column chromatography (petroleum ether/ethyl acetate, 19:1) gave 1-bromo-4-methoxynaphthalene (**2r**) (0.11 g, 89%) as a colorless oil. Spectroscopic data was consistent with the literature.[17] ^{1}H NMR (500 MHz, $CDCl_3$) δ 3.98 (s, 3H), 6.66 (d, J = 8.3 Hz, 1H), 7.55 (ddd, J = 8.3, 6.9, 1.1 Hz, 1H), 7.63 (ddd, J = 8.4, 6.9, 1.2 Hz, 1H), 7.67 (d, J = 8.3 Hz, 1H), 8.21 (br d, J = 8.4 Hz, 1H), 8.31 (br d, J = 8.3 Hz, 1H); ^{13}C NMR (126 MHz, $CDCl_3$) δ 55.7 (CH_3), 104.5 (CH), 113.3 (C), 122.5 (CH), 126.0 (CH), 126.8 (C), 126.9 (CH), 127.8 (CH), 129.5 (CH), 132.5 (C), 155.3 (C); MS (EI) *m/z* 236 (M^+. 100), 221 (42), 193 (54), 114 (44), 84 (35), 63 (11).

5-Bromo-2,3-dihydrobenzofuran (2s)

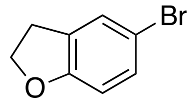

The reaction was performed as described in general procedure A using 2,3-dihydrobenzofuran (**1s**) (56 µL, 0.50 mmol). The reaction mixture was heated to 40 °C for 1 h. Purification by flash column chromatography (petroleum ether/ethyl acetate, 19:1) gave 5-bromo-2,3-dihydrobenzofuran (**2s**) (97 mg, 98%) as a white solid. Mp 48–50 °C; IR (neat) 2903, 1478, 1464, 1231, 1155, 1105, 978, 932, 812 cm^{-1}; ^{1}H NMR (500 MHz, $CDCl_3$) δ 3.20 (t, J = 8.7 Hz, 2H), 4.57 (t, J = 8.7 Hz, 2H), 6.66 (d, J = 8.4 Hz, 1H), 7.19 (dd, J = 8.4, 1.6 Hz, 1H), 7.28 (br s, 1H); ^{13}C NMR (126 MHz, $CDCl_3$) δ 29.8 (CH_2), 71.7 (CH_2), 110.9 (CH), 112.1 (C), 128.0 (CH), 129.6 (C), 130.8 (CH), 159.4 (C); MS (EI) *m/z* 198 (M^+. 100); HRMS (EI) calcd for $C_8H_7{}^{79}BrO$ (M^+), 197.9680, found 197.9680.

3,5-Dibromo-4-hydroxybenzonitrile (2t)

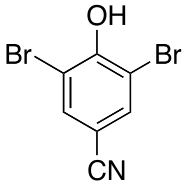

The reaction was performed as described in general procedure A using using 4-cyanophenol (**1t**) (0.060 g, 0.50 mmol) and *N*-bromosuccinimide (0.19 g, 1.1 mmol). The reaction mixture was heated to 40 °C for 2.5 h. Purification by flash column chromatography (petroleum ether/ethyl acetate, 9:1) gave 3,5-dibromo-4-hydroxybenzonitrile (**2t**) (0.13 g, 91%) as a white solid. Mp 190−192 °C; IR (neat) 3406, 2926, 2230, 1464, 1325, 1200, 1134, 1055, 893, 800, 745 cm^{-1}; ^{1}H NMR (500 MHz, $CDCl_3$) δ 6.40 (br s, 1H), 7.77 (s, 2H); ^{13}C NMR (126 MHz, $CDCl_3$) δ 106.6 (C), 110.4 (2 × C), 116.2 (C), 135.7 (2 × CH), 153.6 (C); MS (ESI) *m/z* 276 ($[M–H]^-$. 75); HRMS (ESI) calcd for $C_7H_2{}^{79}Br^{81}BrNO$ ($[M–H]^-$), 275.8488, found 275.8499.

1-(4'-Methoxyphenyl)-1*H*-indole (3a)[18]

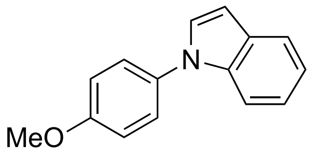

The reaction was performed as described in general procedure B using anisole (**1a**) (108 μL, 1.00 mmol) and indole (176 mg, 1.50 mmol). The bromination step was carried out at 40 °C for 4 h and the *N*-arylation step at 130 °C for 18 h. Purification by flash column chromatography (petroleum ether/ethyl acetate, 19:1) gave 1-(4'-methoxyphenyl)-1*H*-indole (**3a**) (175 mg, 78%) as a white solid. Mp 56–58 °C (lit.[18] 57–59 °C); ^{1}H NMR (500 MHz, $CDCl_3$) δ 3.95 (s, 3H), 6.79 (dd, J = 3.2, 0.8 Hz, 1H), 7.09–7.14 (m, 2H), 7.27–7.36 (m, 2H), 7.39 (d, J = 3.2 Hz, 1H), 7.47–7.52 (m, 2H), 7.59 (dd, J = 8.0, 0.8 Hz, 1H), 7.82 (br d, J = 8.0 Hz, 1H); ^{13}C NMR (126 MHz, $CDCl_3$) δ 55.7 (CH_3), 103.1 (CH), 110.5 (CH), 114.9 (2 × CH), 120.2 (CH), 121.2 (CH), 122.3 (CH), 126.1 (2 × CH), 128.4 (CH), 129.1 (C), 132.9 (C), 136.4 (C), 158.3 (C); MS (ESI) *m/z* 224 (MH^+. 100).

1-(4'-Methoxyphenyl)-1*H*-pyrazole (3b)[19]

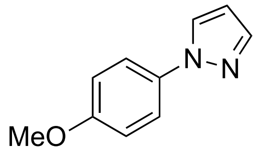

The reaction was performed as described in general procedure B using anisole (**1a**) (1.08 mL, 10.0 mmol) and pyrazole (1.02 g, 15.0 mmol). The bromination step was carried out at 40 °C for 4 h and the *N*-arylation step at 150 °C for 24 h. Purification by flash column chromatography (petroleum ether/ethyl acetate, 19:1) gave 1-(4'-methoxyphenyl)-1*H*-pyrazole (**3b**) (1.65 g, 95%) as a light brown oil. Spectroscopic data was consistent with the literature.[19] ^{1}H NMR (400 MHz, $CDCl_3$) δ 3.84 (s, 3H), 6.44 (dd, J = 2.1, 1.5 Hz, 1H), 6.94–7.00 (m, 2H), 7.56–7.62 (m, 2H), 7.69 (d, J = 1.5 Hz, 1H), 7.82 (d, J = 2.1 Hz, 1H); ^{13}C NMR (101 MHz, $CDCl_3$) δ 55.5 (CH_3), 107.2 (CH), 114.5 (2 × CH), 120.8 (2 × CH), 126.8 (CH), 134.0 (C), 140.6 (CH), 158.2 (C); MS (ESI) *m/z* 197 (MNa^+. 100).

1-(4'-Methoxyphenyl)-1*H*-imidazole (3c)[20]

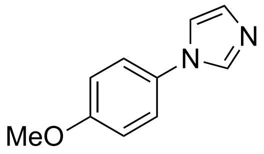

The reaction was performed as described in general procedure B using anisole (**1a**) (108 µL, 1.00 mmol) and imidazole (0.102 g, 1.50 mmol). The bromination step was carried out at 40 °C for 4 h and the *N*-arylation step at 150 °C for 24 h. Purification by flash column chromatography (dichloromethane/methanol 19:1) gave 1-(4'-methoxyphenyl)-1*H*-imidazole (**3c**) (132 mg, 76%) as a white solid. Mp 60–62 °C (lit.[20] 61–63 °C); ^{1}H NMR (500 MHz, $CDCl_3$) δ 3.79 (s, 3H), 6.90–6.95 (m, 2H), 7.10–7.20 (m, 2H), 7.21–7.27 (m, 2H), 7.72 (1H, br s, 2-H); ^{13}C NMR (126 MHz, $CDCl_3$) δ 55.6 (CH_3), 114.9 (2 × CH), 118.8 (CH), 123.1 (2 × CH), 130.1 (CH), 130.7 (C), 135.8 (CH), 158.9 (C); MS (ESI) *m/z* 175 (MH^+. 100), 160.

1-(4'-Methoxyphenyl)-1*H*-pyrrole (3d)[21]

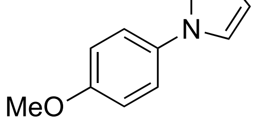

The reaction was performed as described in general procedure B using anisole (**1a**) (108 µL, 1.00 mmol) and pyrrole (104 µL, 1.50 mmol). The bromination step was carried out at 40 °C

for 4 h and the *N*-arylation step at 150 °C for 36 h. Purification by flash column chromatography (petroleum ether) gave 1-(4’-methoxyphenyl)-1*H*-pyrrole (**3d**) (88.0 mg, 51%) as a white solid. Mp 98–100 °C (lit.[21] 104–108 °C); ^{1}H NMR (500 MHz, $CDCl_3$) δ 3.85 (s, 3H), 6.35 (br s, 2H), 6.94–6.99 (m, 2H), 7.02 (br s, 2H), 7.31–7.35 (m, 2H); ^{13}C NMR (126 MHz, $CDCl_3$) δ 55.6 (CH_3), 109.9 (2 × CH), 114.6 (2 × CH), 119.7 (2 × CH), 122.2 (2 × CH), 134.5 (C), 157.7 (C); MS (ESI) *m/z* 196 (MNa^+. 100).

1-(4’-Methoxyphenyl)pyrrolidin-2-one (3e)[22]

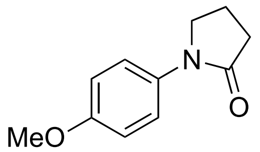

The reaction was performed as described in general procedure B using anisole (**1a**) (108 µL, 1.00 mmol) and 2-pyrrolidone (128 mg, 1.50 mmol). The bromination step was carried out at 40 °C for 4 h and the *N*-arylation step at 150 °C for 24 h. Purification by flash column chromatography (dichloromethane/methanol 49:1) gave 1-(4’-methoxyphenyl)pyrrolidin-2-one (**3e**) (111 mg, 58%) as an off-white solid. Mp 112–114 °C (lit.[22] 113–114 °C); ^{1}H NMR (500 MHz, $CDCl_3$) δ 2.14 (quin., J = 7.9 Hz, 2H), 2.58 (t, J = 7.9 Hz, 2H), 3.79 (s, 3H), 3.81 (t, J = 7.9 Hz, 2H), 6.86–6.92 (m, 2H), 7.46–7.51 (m, 2H); ^{13}C NMR (126 MHz, $CDCl_3$) δ 18.0 (CH_2), 32.5 (CH_2), 49.2 (CH_2), 55.5 (CH_3), 114.0 (2 × CH), 121.8 (2 × CH), 132.6 (C), 156.6 (C), 173.9 (C); MS (ESI) *m/z* 214 (MNa^+. 100).

***N*-(4’-Methoxyphenyl)benzamide (3f)[23]**

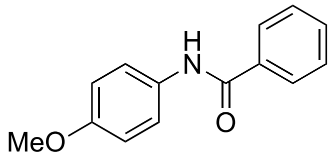

The reaction was performed as described in general procedure B using anisole (**1a**) (108 µL, 1.00 mmol) and benzamide (182 mg, 1.50 mmol). The bromination step was carried out at 40 °C for 4 h and the *N*-arylation step at 130 °C for 18 h. Purification by flash column chromatography (petroleum ether/ethyl acetate, 8:2) gave *N*-(4’-methoxyphenyl)benzamide (**3f**) (177 mg, 78%) as a white solid. Mp 152–154 °C (lit.[23] 153–155 °C); ^{1}H NMR (500 MHz, $CDCl_3$) δ 3.81 (s, 3H), 6.87–6.93 (m, 2H), 7.44–7.56 (m, 5H), 7.80 (br s, 1H), 7.83–7.88 (m, 2H); ^{13}C NMR (126 MHz, $CDCl_3$) δ 55.5 (CH_3), 114.3 (2 × CH), 122.1 (2 × CH), 127.0 (2 ×

CH), 128.7 (2 × CH), 131.0 (C), 131.7 (CH), 135.1 (C), 156.7 (C), 165.6 (C); MS (ESI) *m/z* 250 (MNa^+. 100).

***N*-(4'-Methoxyphenyl)-4-methylbenzamide (3g)**[23]

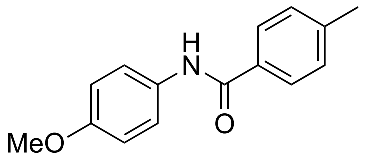

The reaction was performed as described in general procedure B using anisole (**1a**) (108 µL, 1.00 mmol) and *p*-toluamide (203 mg, 1.50 mmol). The bromination step was carried out at 40 °C for 4 h and the *N*-arylation step at 130 °C for 18 h. Purification by flash column chromatography (petroleum ether/ethyl acetate, 8:2) gave *N*-(4'-methoxyphenyl)-4-methylbenzamide (**3g**) (213 mg, 88%) as a light brown solid. Mp 156–158 °C (lit.[23] 157–159 °C); ^{1}H NMR (500 MHz, $CDCl_3$) *δ* 2.40 (s, 3H), 3.80 (s, 3H), 6.85–6.90 (m, 2H), 7.24 (d, *J* = 8.0 Hz, 2H), 7.50–7.55 (m, 2H), 7.75 (d, *J* = 8.0 Hz, 2H), 7.88 (br s, 1H); ^{13}C NMR (126 MHz, $CDCl_3$) *δ* 21.5 (CH_3), 55.5 (CH_3), 114.2 (2 × CH), 122.2 (2 × CH), 127.0 (2 × CH), 129.4 (2 × CH), 131.2 (C), 132.2 (C), 142.1 (C), 156.5 (C), 165.7 (C); MS (ESI) *m/z* 264 (MNa^+. 100).

***N*-(4'-Methoxyphenyl)benzenesulfonamide (3h)**[24]

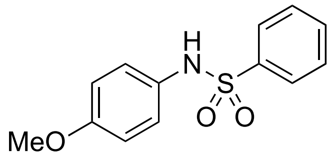

The reaction was performed as described in general procedure B using anisole (**1a**) (108 µL, 1.00 mmol) and benzenesulfonamide (236 mg, 1.50 mmol). The bromination step was carried out at 40 °C for 4 h and the *N*-arylation step at 130 °C for 18 h. Purification by flash column chromatography (petroleum ether/ethyl acetate, 9:1) gave *N*-(4'-methoxyphenyl)benzenesulfonamide (**3h**) (231 mg, 88%) as a brown solid. Mp 90–92 °C. Spectroscopic data was consistent with the literature.[24] ^{1}H NMR (400 MHz, $CDCl_3$) *δ* 3.75 (s, 3H), 6.71–6.78 (m, 3H), 6.95–7.01 (m, 2H), 7.39–7.45 (m, 2H), 7.53 (tt, *J* = 7.6, 1.2 Hz, 1H), 7.70–7.74 (m, 2H); ^{13}C NMR (101 MHz, $CDCl_3$) *δ* 55.4 (CH_3), 114.5 (2 × CH), 125.3 (2 × CH), 127.3 (2 × CH), 129.0 (2 × CH and C), 132.9 (CH), 138.9 (C), 157.9 (C); MS (ESI) *m/z* 286 (MNa^+. 100).

4-Fluoro-*N*-(4'-methoxyphenyl)benzenesulfonamide (3i)

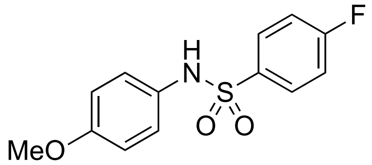

The reaction was performed as described in general procedure B using anisole (**1a**) (108 µL, 1.00 mmol) and 4-fluorobenzenesulfonamide (263 mg, 1.50 mmol). The bromination step was carried out at 40 °C for 4 h and the *N*-arylation step at 130 °C for 18 h. Purification by flash column chromatography (petroleum ether/ethyl acetate, 8:2) gave 4-fluoro-*N*-(4'-methoxyphenyl)benzenesulfonamide (**3i**) (231 mg, 82%) as a light brown solid. Mp 102–104 °C; IR (neat) 3262, 2937, 1592, 1508, 1495, 1247, 1241, 1165, 1153, 1090, 837, 754 cm^{-1}; ^{1}H NMR (500 MHz, $CDCl_3$) δ 3.76 (s, 3H), 6.74–6.79 (m, 3H), 6.95–7.00 (m, 2H), 7.06–7.12 (m, 2H), 7.69–7.74 (m, 2H); ^{13}C NMR (126 MHz, $CDCl_3$) δ 55.4 (CH_3), 114.5 (2 × CH), 116.2 (2 × CH, d, $^{2}J_{CF}$ = 22.6 Hz), 125.7 (2 × CH), 128.5 (C), 130.1 (2 × CH, d, $^{3}J_{CF}$ = 9.4 Hz), 134.9 (C, d, $^{4}J_{CF}$ = 3.2 Hz), 158.2 (C), 165.2 (C, d, $^{1}J_{CF}$ = 255.1 Hz); MS (ESI) *m/z* 304 (MNa^{+}. 100); HRMS (ESI) calcd for $C_{13}H_{12}FNNaO_3S$ (MNa^{+}), 304.0414, found 304.0405.

1-(4'-Methoxy-3'-methylphenyl)-1*H*-pyrazole (4a)

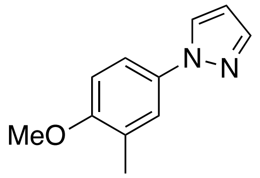

The reaction was performed as described in general procedure B using 2-methylanisole (**1f**) (124 µL, 1.00 mmol) and pyrazole (102 mg, 1.50 mmol). The bromination step was carried out at 40 °C for 4 h and the *N*-arylation step at 150 °C for 20 h. Purification by flash column chromatography (petroleum ether/ethyl acetate, 19:1) gave 1-(4'-methoxy-3'-methylphenyl)-1*H*-pyrazole (**4a**) (152 mg, 81%) as a colorless oil. IR (neat) 2928, 1519, 1504, 1239, 1046, 909, 730 cm^{-1}; ^{1}H NMR (500 MHz, $CDCl_3$) δ 2.28 (s, 3H), 3.86 (s, 3H), 6.42 (t, J = 2.0 Hz, 1H), 6.86 (d, J = 8.7 Hz, 1H), 7.42 (dd, J = 8.7, 2.6 Hz, 1H), 7.48 (d, J = 2.6 Hz, 1H), 7.68 (d, J = 2.0 Hz, 1H), 7.81 (d, J = 2.0 Hz, 1H); ^{13}C NMR (126 MHz, $CDCl_3$) δ 16.4 (CH_3), 55.6 (CH_3), 107.0 (CH), 110.2 (CH), 117.8 (CH), 122.3 (CH), 126.8 (CH), 127.8 (C), 133.5 (C), 140.5 (CH), 156.4 (C); MS (ESI) *m/z* 211 (MNa^{+}, 100); HRMS (ESI) calcd for $C_{11}H_{12}N_2NaO$ (MNa^{+}), 211.0842, found 211.0838.

4’-(1*H*-Pyrazol-1-yl)phenol (4b)[25]

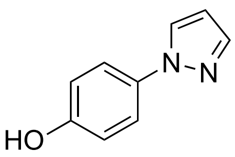

The reaction was performed as described in general procedure B using phenol (**1g**) (94.0 mg, 1.00 mmol) and pyrazole (102 mg, 1.50 mmol). The bromination step was carried out at 20 °C for 1 h and the *N*-arylation step at 150 °C for 20 h. Purification by flash column chromatography (dichloromethane/methanol 49:1) gave 4’-(1*H*-pyrazol-1-yl)phenol (**4b**) (106 mg, 66%) as a light brown oil. Spectroscopic data was consistent with the literature.[25] ^{1}H NMR (500 MHz, $CDCl_3$) δ 6.45 (t, J = 2.0 Hz, 1H), 6.80–6.84 (m, 2H), 7.40–7.44 (m, 2H), 7.72 (d, J = 2.0 Hz, 1H), 7.78 (d, J = 2.0 Hz, 1H); ^{13}C NMR (126 MHz, $CDCl_3$) δ 107.2 (CH), 116.3 (2 × CH), 122.0 (2 × CH), 128.1 (CH), 132.8 (C), 140.4 (CH), 156.1 (C); MS (ESI) *m/z* 159 ([M–H]$^{-}$. 100).

2’-Fluoro-4’-(1*H*-pyrazol-1-yl)phenol (4c)

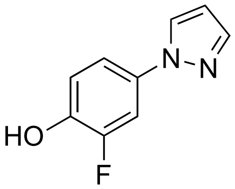

The reaction was performed as described in general procedure B using 2-fluorophenol (**1h**) (89.0 μL, 1.00 mmol) and pyrazole (102 mg, 1.50 mmol). The bromination step was carried out at 40 °C for 3 h and the *N*-arylation step at 130 °C for 20 h. Purification by flash column chromatography (petroleum ether/ethyl acetate, 9:1) gave 2’-fluoro-4’-(1*H*-pyrazol-1-yl)phenol (**4c**) (104 mg, 64%) as a yellow oil. IR (neat) 3123, 2970, 1526, 1516, 1401, 1288, 1248, 1186, 1039, 753 cm^{-1}; ^{1}H NMR (500 MHz, $CDCl_3$) δ 6.45 (t, J = 2.0 Hz, 1H), 6.49 (s, 1H), 7.02 (t, J = 8.9 Hz, 1H), 7.28 (ddd, J = 8.9, 2.5, 1.4 Hz, 1H), 7.44 (dd, J = 11.4, 2.5 Hz, 1H), 7.71 (d, J = 2.0 Hz, 1H), 7.80 (d, J = 2.0 Hz, 1H); ^{13}C NMR (126 MHz, $CDCl_3$) δ 107.6 (CH), 108.9 (CH, d, $^{2}J_{CF}$ = 22.6 Hz), 116.2 (CH, d, $^{3}J_{CF}$ = 3.3 Hz), 118.1 (CH, d, $^{4}J_{CF}$ = 3.1 Hz), 127.7 (CH), 133.0 (C, d, $^{3}J_{CF}$ = 8.6 Hz), 140.9 (CH), 143.2 (C, d, $^{2}J_{CF}$ = 13.7 Hz), 151.3 (C, d, $^{1}J_{CF}$ = 241.2 Hz); MS (ESI) *m/z* 177 ([M–H]$^{-}$. 100); HRMS (ESI) calcd for $C_9H_6FN_2O$ ([M–H]$^{-}$), 177.0470, found 177.0465. ([M–H]$^{-}$.

4'-(1*H*-Pyrazol-1-yl)-2'-(trifluoromethyl)aniline (4d)

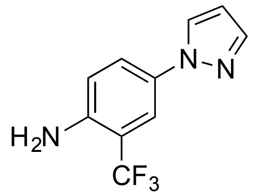

The reaction was performed as described in general procedure B using 2-trifluoromethylaniline (**1j**) (126 μL, 1.00 mmol) and pyrazole (102 mg, 1.50 mmol). The bromination step was carried out at 40 °C for 7 h and the *N*-arylation step at 150 °C for 20 h. Purification by flash column chromatography (petroleum ether/ethyl acetate, 9:1) gave 4'-(1*H*-pyrazol-1-yl)-2'-(trifluoromethyl)aniline (**4d**) (148 mg, 65%) as a brown oil. IR (neat) 3365, 2935, 1641, 1521, 1506, 1299, 1142, 1106, 1037, 749 cm^{-1}; ^{1}H NMR (500 MHz, $CDCl_3$) δ 4.24 (br s, 2H), 6.44 (t, J = 2.0 Hz, 1H), 6.81 (d, J = 8.7 Hz, 1H), 7.61 (dd, J = 8.7, 2.4 Hz, 1H), 7.69 (d, J = 2.0 Hz, 1H), 7.74 (d, J = 2.4 Hz, 1H), 7.80 (d, J = 2.0 Hz, 1H); ^{13}C NMR (126 MHz, $CDCl_3$) δ 107.4 (CH), 113.9 (C, q, $^{2}J_{CF}$ = 30.7 Hz), 118.0 (CH), 118.2 (CH, q, $^{3}J_{CF}$ = 5.5 Hz), 124.4 (C, q, $^{1}J_{CF}$ = 272.5 Hz), 124.5 (CH), 126.8 (CH), 131.4 (C), 140.7 (CH), 143.2 (C); MS (ESI) *m/z* 250 (MNa^{+}. 100); HRMS (ESI) calcd for $C_{10}H_8F_3N_3Na$ (MNa^{+}), 250.0563, found 250.0556.

***N*-[4'-(1*H*-Pyrazol-1-yl)phenyl]acetamide (4e)**

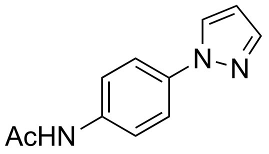

The reaction was performed as described in general procedure B using acetanilide (**1n**) (136 mg, 1.00 mmol) and pyrazole (102 mg, 1.50 mmol). The bromination step was carried out at 40 °C for 6 h and the *N*-arylation step at 150 °C for 20 h. Purification by flash column chromatography (petroleum ether/ethyl acetate, 7:3) gave *N*-(4'-(1*H*-pyrazol-1-yl)phenyl)acetamide (**4e**) (152 mg, 76%) as a brown solid. Mp 134–136 °C; IR (neat) 3304, 3062, 1667, 1614, 1555, 1525, 1398, 1330, 1054, 1036, 940, 826 cm^{-1}; ^{1}H NMR (500 MHz, $CDCl_3$) δ 2.16 (s, 3H), 6.45 (t, J = 2.1 Hz, 1H), 7.55–7.61 (m, 4H), 7.70 (d, J = 2.1 Hz, 1H), 7.80 (br s, 1H), 7.86 (d, J = 2.1 Hz, 1H); ^{13}C NMR (126 MHz, $CDCl_3$) δ 24.4 (CH_3), 107.6 (CH), 119.9 (2 × CH), 120.9 (2 × CH), 127.0 (CH), 136.4 (C), 136.6 (C), 141.0 (CH), 169.0 (C); MS (ESI) *m/z* 224 (MNa^{+}, 100); HRMS (ESI) calcd for $C_{11}H_{11}N_3NaO$ (MNa^{+}), 224.0794, found 224.0791.

N-[2'-Chloro-4'-(1H-pyrazol-1'-yl)phenyl]acetamide (4f)

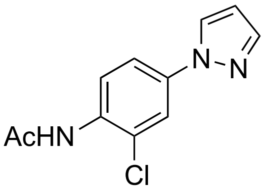

The reaction was performed as described in general procedure B using *N*-(2-chlorophenyl)acetamide (**1o**) (170 mg, 1.00 mmol) and pyrazole (102 mg, 1.50 mmol). The bromination step was carried out at 40 °C for 7 h and the *N*-arylation step at 150 °C for 20 h. Purification by flash column chromatography (petroleum ether/ethyl acetate, 9:1) gave *N*-[2'-chloro-4'-(1*H*-pyrazol-1'-yl)phenyl]acetamide (**4f**) (149 mg, 63%) as a brown solid. Mp 120–122 °C; IR (neat) 3264, 3011, 1671, 1522, 1396, 1302, 1053, 944, 757 cm^{-1}; ^{1}H NMR (500 MHz, $CDCl_3$) δ 2.26 (s, 3H), 6.47 (t, J = 2.1 Hz, 1H), 7.53 (dd, J = 9.0, 2.5 Hz, 1H), 7.62 (br s, 1H), 7.71 (d, J = 2.1 Hz, 1H), 7.83 (d, J = 2.5 Hz, 1H), 7.87 (d, J = 2.1 Hz, 1H), 8.47 (d, J = 9.0 Hz, 1H); ^{13}C NMR (126 MHz, $CDCl_3$) δ 24.8 (CH_3), 108.0 (CH), 117.8 (CH), 119.9 (CH), 122.3 (CH), 123.6 (C), 126.7 (CH), 132.9 (C), 136.4 (C), 141.4 (CH), 168.4 (C); MS (ESI) *m/z* 258 (MNa^+, 100); HRMS (ESI) calcd for $C_{11}H_{10}N_3NaO^{35}Cl$ (MNa^+), 258.0405, found 258.0401.

N-[2'-Acetyl-4'-(1H-pyrazol-1-yl)phenyl]acetamide (4g)

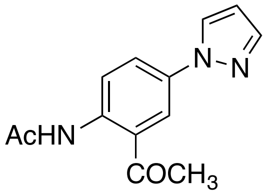

The reaction was performed as described in general procedure B using *N*-(2-acetylphenyl)acetamide (**1p**) (178 mg, 1.00 mmol) and pyrazole (102 mg, 1.50 mmol). The bromination step was carried out at 40 °C for 7 h and the *N*-arylation step at 130 °C for 20 h. Purification by flash column chromatography (petroleum ether/ethyl acetate, 8:2) gave *N*-[2'-acetyl-4'-(1*H*-pyrazol-1-yl)phenyl]acetamide (**4g**) (129 mg, 53%) as a yellow solid. Mp 146–148 °C; IR (neat) 3241, 3001, 1693, 1655, 1594, 1527, 1509, 1361, 1224, 1041, 946, 755 cm^{-1}; ^{1}H NMR (500 MHz, $CDCl_3$) δ 2.22 (s, 3H), 2.71 (s, 3H), 6.47 (t, J = 2.1 Hz, 1H), 7.68–7.73 (m, 2H), 7.90 (d, J = 2.1 Hz, 1H), 8.29 (d, J = 2.6 Hz, 1H), 8.82 (d, J = 9.1 Hz, 1H), 11.59 (br s, 1H); ^{13}C NMR (126 MHz, $CDCl_3$) δ 25.5 (CH_3), 28.8 (CH_3), 108.0 (CH), 121.6 (CH), 122.3 (C), 122.3 (CH), 124.8 (CH), 126.7 (CH), 134.6 (C), 139.2 (C), 141.3 (CH),

169.5 (C), 202.4 (C); MS (ESI) *m/z* 266 (MNa^+, 100); HRMS (ESI) calcd for $C_{13}H_{13}N_3NaO_2$ (MNa^+), 266.0900, found 266.0904.

1-(2',3'-Dihydrobenzofuran-5'-yl)-1*H*-pyrazole (4h)

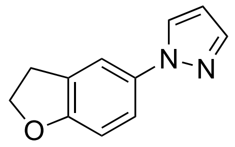

The reaction was performed as described in general procedure B using 2,3-dihydrobenzofuran (**1s**) (113 µL, 1.00 mmol) and pyrazole (102 mg, 1.50 mmol). The bromination step was carried out at 40 °C for 1.5 h and the *N*-arylation step at 150 °C for 20 h. Purification by flash column chromatography (petroleum ether/ethyl acetate, 19:1) gave 1-(2',3'-dihydrobenzofuran-5'-yl)-1*H*-pyrazole (**4h**) (157 mg, 84%) as a brown oil. IR (neat) 2974, 1516, 1492, 1394, 1228, 1029, 983, 942, 746 cm^{-1}; ^1H NMR (400 MHz, $CDCl_3$) δ 3.25 (t, J = 8.7 Hz, 2H), 4.62 (t, J = 8.7 Hz, 2H), 6.41 (t, J = 2.1 Hz, 1H), 6.81 (d, J = 8.5 Hz, 1H), 7.35 (dd, J = 8.5, 2.4 Hz, 1H), 7.51–7.54 (m, 1H), 7.67 (d, J = 2.1 Hz, 1H), 7.78 (d, J = 2.1 Hz, 1H); ^{13}C NMR (101 MHz, $CDCl_3$) δ 29.7 (CH_2), 71.7 (CH_2), 107.0 (CH), 109.3 (CH), 117.2 (CH), 119.7 (CH), 126.9 (CH), 128.3 (C), 134.1 (C), 140.4 (CH), 158.8 (C); MS (ESI) *m/z* 209 (MNa^+, 100); HRMS (ESI) calcd for $C_{11}H_{10}N_2NaO$ (MNa^+), 209.0685, found 209.0682.

***N*-(4'-Methoxy-3'-methylphenyl)benzamide (4i)[26]**

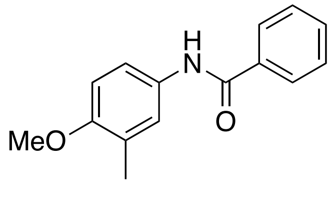

The reaction was performed as described in general procedure B using 2-methylanisole (**1f**) (124 µL, 1.00 mmol) and benzamide (182 mg, 1.50 mmol). The bromination step was carried out at 40 °C for 4 h and the *N*-arylation step at 130 °C for 20 h. Purification by flash column chromatography (petroleum ether/ethyl acetate, 8:2) gave *N*-(4'-methoxy-3'-methylphenyl)benzamide (**4i**) (156 mg, 65%) as a light purple solid. Mp 148–150 °C. Spectroscopic data was consistent with the literature.[26] ^1H NMR (400 MHz, $CDCl_3$) δ 2.22 (s, 3H), 3.83 (s, 3H), 6.80 (d, J = 8.7 Hz, 1H), 7.36 (d, J = 1.8 Hz, 1H), 7.42–7.49 (m, 3H), 7.53 (tt, J = 7.4, 1.2 Hz, 1H), 7.76 (br s, 1H), 7.83–7.88 (m, 2H); ^{13}C NMR (101 MHz, $CDCl_3$) δ 16.3 (CH_3), 55.6 (CH_3), 110.2 (CH), 119.2 (CH), 123.5 (CH), 127.0 (2 × CH), 127.3 (C),

128.7 (2 × CH), 130.5 (C), 131.6 (CH), 135.1 (C), 154.9 (C), 165.6 (C); MS (ESI) *m/z* 264 (MNa^+. 100).

_N_-[4’-Amino-3’-(trifluoromethyl)phenyl]benzamide (4j)

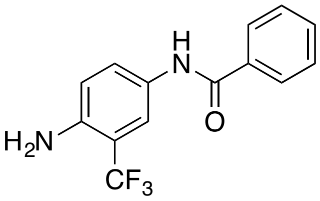

The reaction was performed as described in general procedure B using 2-trifluoromethylaniline (**1j**) (126 µL, 1.00 mmol) and benzamide (133 mg, 1.10 mmol). The bromination step was carried out at 40 °C for 7 h and the *N*-arylation step at 130 °C for 20 h. Purification by flash column chromatography (petroleum ether/ethyl acetate, 8:2) gave *N*-[4’-amino-3’-(trifluoromethyl)phenyl]benzamide (**4j**) (198 mg, 71%) as a light brown solid. Mp 118–120 °C; IR (neat) 3282, 2927, 1643, 1508, 1432, 1310, 1225, 1104, 1051, 694 cm^{-1}; ^1H NMR (500 MHz, $CDCl_3$) δ 4.13 (s, 2H), 6.77 (d, J = 8.7 Hz, 1H), 7.46−7.51 (m, 2H), 7.53−7.58 (m, 1H), 7.59−7.67 (m, 2H), 7.70 (s, 1H), 7.83−7.87 (m, 2H); ^{13}C NMR (126 MHz, $CDCl_3$) δ 113.8 (C, q, $^2J_{CF}$ = 30.3 Hz), 117.7 (CH), 119.5 (CH, q, $^3J_{CF}$ = 5.1 Hz), 124.5 (C, q, $^1J_{CF}$ = 272.2 Hz), 126.4 (CH), 127.0 (2 × CH), 128.4 (C), 128.7 (2 × CH), 131.8 (CH), 134.6 (C), 141.6 (C), 166.0 (C); MS (ESI) *m/z* 303 (MNa^+, 100); HRMS (ESI) calcd for $C_{14}H_{11}F_3N_2NaO$ (MNa^+), 303.0716, found 303.0711.

_N_-(4’-Acetamidophenyl)benzamide (4k)[27]

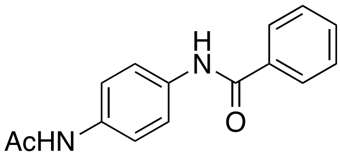

The reaction was performed as described in general procedure B using 2-trifluoromethylaniline (**1n**) (136 mg, 1.00 mmol) and benzamide (133 mg, 1.10 mmol). The bromination step was carried out at 40 °C for 6 h and the *N*-arylation step at 130 °C for 20 h. Purification by flash column chromatography (dichloromethane/methanol, 98:2) gave *N*-(4’-acetamidophenyl)benzamide (**4k**) (176 mg, 77%) as a brown solid. Mp 146–148 °C. Spectroscopic data was consistent with the literature.[27] ^1H NMR (500 MHz, DMSO-d_6) δ 2.03 (s, 3H), 7.49−7.60 (m, 5H), 7.66−7.71 (m, 2H), 7.92−7.96 (m, 2H), 9.91 (s, 1H), 10.19 (s, 1H); ^{13}C NMR (126 MHz, DMSO-d_6) δ 24.4 (CH_3), 119.7 (2 × CH), 121.3 (2 × CH), 128.0 (2

× CH), 128.8 (2 × CH), 131.9 (CH), 134.8 (C), 135.5 (C), 135.7 (C), 165.7 (C), 168.5 (C); *m/z* (EI) 254 (M^+. 100), 212 (20), 105 (89), 77 (32).

_N_-(2’,3’-Dihydrobenzofuran-5’-yl)benzamide (4l)

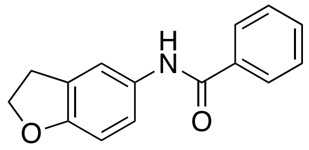

The reaction was performed as described in general procedure B using 2,3-dihydrobenzofuran (**1s**) (113 µL, 1.00 mmol) and benzamide (182 mg, 1.50 mmol). The bromination step was carried out at 40 °C for 1.5 h and the *N*-arylation step at 130 °C for 20 h. Purification by flash column chromatography (petroleum ether/ethyl acetate, 8:2) gave *N*-(2’,3’-dihydrobenzofuran-5’-yl)benzamide (**4l**) (162 mg, 68%) as a brown solid. Mp 156–158 °C; IR (neat) 3268, 2895, 1641, 1529, 1491, 1220, 983, 692 cm^{-1}; ^{1}H NMR (500 MHz, $CDCl_3$) δ 3.21 (t, J = 8.7 Hz, 2H), 4.58 (t, J = 8.7 Hz, 2H), 6.74 (d, J = 8.4 Hz, 1H), 7.14 (dd, J = 8.4, 1.5 Hz, 1H), 7.42–7.56 (m, 3H), 7.63 (br s, 1H), 7.78–7.88 (m, 3H); ^{13}C NMR (126 MHz, $CDCl_3$) δ 29.9 (CH_2), 71.5 (CH_2), 109.1 (CH), 118.6 (CH), 120.8 (CH), 127.0 (2 × CH), 127.8 (C), 128.7 (2 × CH), 130.8 (C), 131.7 (CH), 135.1 (C), 157.2 (C), 165.7 (C); MS (ESI) *m/z* 262 (MNa^+, 100); HRMS (ESI) calcd for $C_{15}H_{13}NNaO_2$ (MNa^+), 262.0838, found 262.0836.

1-(5'-Chloro-2'-methoxyphenyl)-1_H_-pyrazole (4m)

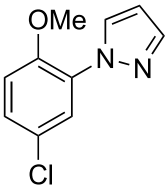

The reaction was performed as described in general procedure B using 4-chloroanisole (**1c**) (123 µL, 1.00 mmol), iron(III) trichloride (0.05 mmol), [BMIM]NTf_2 (0.15 mmol) and pyrazole (102 mg, 1.50 mmol). The bromination step was carried out at 70 °C for 5 h and the *N*-arylation step at 150 °C for 20 h. Purification by flash column chromatography (petroleum ether/ethyl acetate, 9:1) gave 1-(5'-chloro-2'-methoxyphenyl)-1*H*-pyrazole (**4m**) (110 mg, 53%) as a colorless oil. IR (neat) 2943, 1521, 1495, 1244, 1024, 750, 704 cm^{-1}; ^{1}H NMR (500 MHz, $CDCl_3$) δ 3.88 (s, 3H), 6.43 (dd, J = 2.4, 2.0 Hz, 1H), 6.96 (d, J = 8.8 Hz, 1H), 7.23 (dd, J = 8.8, 2.6 Hz, 1H), 7.70 (d, J = 2.0 Hz, 1H), 7.81 (d, J = 2.6 Hz, 1H), 8.09 (d, J = 2.4 Hz, 1H); ^{13}C NMR (126 MHz, $CDCl_3$) δ 56.2 (CH_3), 106.7 (CH), 113.4 (CH), 124.7 (CH), 126.1

(C), 127.2 (CH), 130.3 (C), 131.6 (CH), 140.4 (CH), 149.5 (C); MS (ESI) *m/z* 231 (MNa^+, 100); HRMS (ESI) calcd for $C_{10}H_9{}^{35}ClN_2NaO$ (MNa^+), 231.0296, found 231.0291.

5. References

1. J. Pan, X. Wang, Y. Zhang and S. Buchwald, *Org. Lett.*, **2011**, *13*, 4974–4976.
2. K. Ohsawa, M. Yoshida, and T. Doi, *J. Org. Chem.*, **2013**, *78*, 3438–3444.
3. M. Scheepstra, L. Nieto, A. K. H. Hirsch, S. Fuchs, S. Leysen, C. V. Lam, L. in het Panhuis, C. A. A. van Boeckel, H. Wienk, R. Boelens, C. Ottmann, L-G. Milroy and L. Brunsveld, *Angew. Chem. Int. Ed.*, **2014**, *53*, 6443–6448; *Angew. Chem.* **2014**, *126*, 6561–6566.
4. S. D. Wyrick, F. T. Smith, W. E. Kemp and A. A. Grippo, *J. Med. Chem.*, **1987**, *30*, 1798–1806.
5. Z. Wang, Z. Liu, W. Lee, S-N. Kim, G. Yoon and S. H. Cheon, *Bioorg. Med. Chem. Lett.*, **2014**, *24*, 3337–3340.
6. M. Li and G. A. O'Doherty, *Org. Lett.*, **2006**, *8*, 3987–3990.
7. E. J. Rayment, N. Summerhill and E. A. Anderson, *J. Org. Chem.*, **2012**, *77*, 7052–7060.
8. J. Li, D. Smith, J. X. Qiao, S. Huang, S. Krishnananthan, H. S. Wong, M. E. Salvati, B. N. Balasubramanian and B.-C. Chen, *Synlett*, **2009**, 633–637.
9. J. Xie, X. Yuan, A. Abdukader, C. Zhu and J. Ma, *Org. Lett.*, **2014**, *16*, 1768–1771.
10. J-M Chrétien, F. Zammattio, E. L. Grognec, M. Paris, B. Cahingt, G. Montavon and J-P. Quintard, *J. Org. Chem.*, **2005**, *70*, 2870–2873.
11. A. Podgoršek, S. Stavber, M. Zupan and J. Iskra, *Tetrahedron*, **2009**, *65*, 4429–4439.
12. E. Giovannini and B. F. S. E. De Sousa, *Helv. Chim. Acta*, **1979**, *62*, 198–204.
13. U. P. Saikia, F. L. Hussain, M. Suri and P. Pahari , *Tetrahedron Lett.,* **2016**, *57*, 1158–1160.
14. T. Mahajan, L. Kumar, K. Dwivedi, and D. D. Agarwal, *Synth. Commun.* **2012**, *42*, 3655–3663.
15. F. Han, S. Lin, P. Liu, X. Liu, J. Tao, X. Deng, C. Yi and H. Xu, *ACS Med. Chem. Lett.* **2015**, *6*, 434–438.
16. N. J. Bunce, *J. Chem. Soc., Perkin Trans. 1*, **1974**, 942–944.
17. A. Bose and P. Mal, *Tetrahedron Lett.*, **2014**, *55*, 2154–2156.
18. R. K. Rao, A. B. Naidu, E. A. Jaseer and G. Sekar, *Tetrahedron*, **2009**, *65*, 4619–4624.
19. C-T. Yang, Y. Fu, Y-B. Huang, J. Yi, Q-X. Guo and L. Liu, *Angew. Chem. Int. Ed.* **2009**, *48*, 7398–7401; *Angew. Chem.* **2009**, *121*, 7534–7537.

20. S. Jammi, S. Sakthivel, L. Rout, T. Mukherjee, S. Mandal, R. Mitra, P. Saha and T. Punniyamurthy, *J. Org. Chem.*, **2009**, *74*, 1971–1976.
21. C. K. Lee, J. H. Jun and J. S. Yu, *J. Heterocyclic Chem.,* **2000**, *37*, 15–24.
22. C. P. A. T. Lawson, A. M. Z. Slawin and N. J. Westwood, *Chem. Commun.,* **2011**, *47*, 1057–1059.
23. N. Sharma and G. Sekar, *Adv. Synth. Catal*. **2016**, *358*, 314–320.
24. K. Yang, M. Ke, Y. Lin and Q. Song, *Green Chem*., **2015**, *17*, 1395–1399.
25. J-H. Lee, H. Kim, T. Kim, J. H. Song, W-S. Kim and J. Ham, *Bull. Korean Chem. Soc*. **2013**, *34*, 42–48.
26. S.-K. Xiang, J.-M. Li, H. Huang, C. Feng, H.-L. Ni, X.-Z. Chen, B.-Q. Wang, K.-Q. Zhao, P. Hu and C. Redshaw, *Adv. Synth. Catal*., **2015**, *357*, 3435–3440.
27. E. Martini, M. Norcini, C. Ghelardini, D. Manetti, S. Dei, L. Guandalini, M. Melchiorre, S. Pagella, S. Scapecchi, E. Teodori and M. N. Romanelli, *Bioorg. Med. Chem*., **2008**, *16*, 10034–10042.

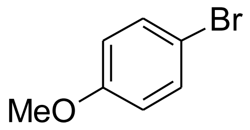
Br
MeO

2a

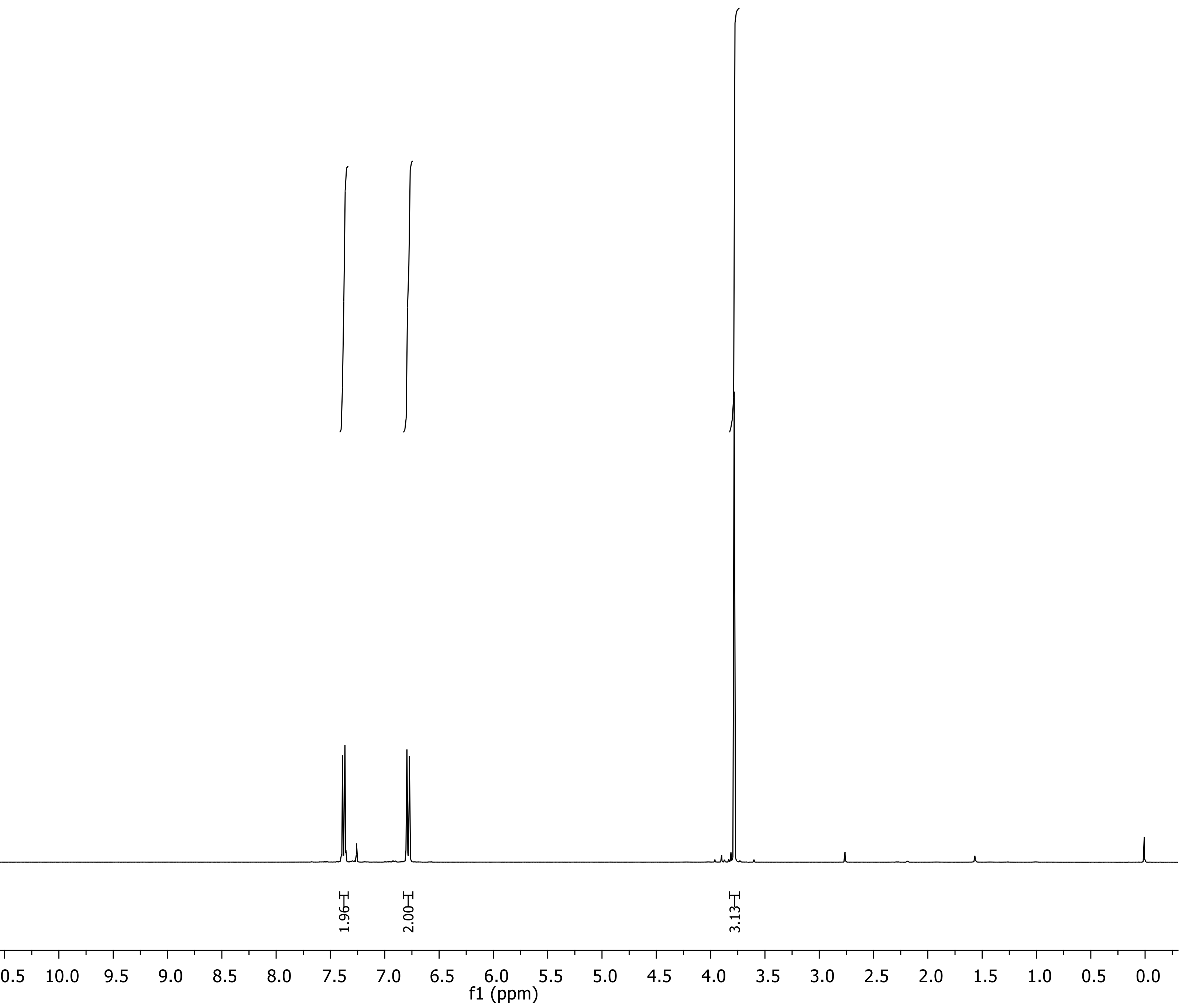
1.96
2.00
3.13
12.0
11.5
11.0
10.5
10.0
9.5
9.0
8.5
8.0
7.5
7.0
6.5
6.0
5.5
5.0
4.5
4.0
3.5
3.0
2.5
2.0
1.5
1.0
0.5
0.0
f1 (ppm)

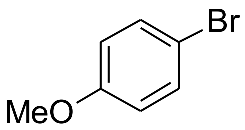

Br
MeO

2a

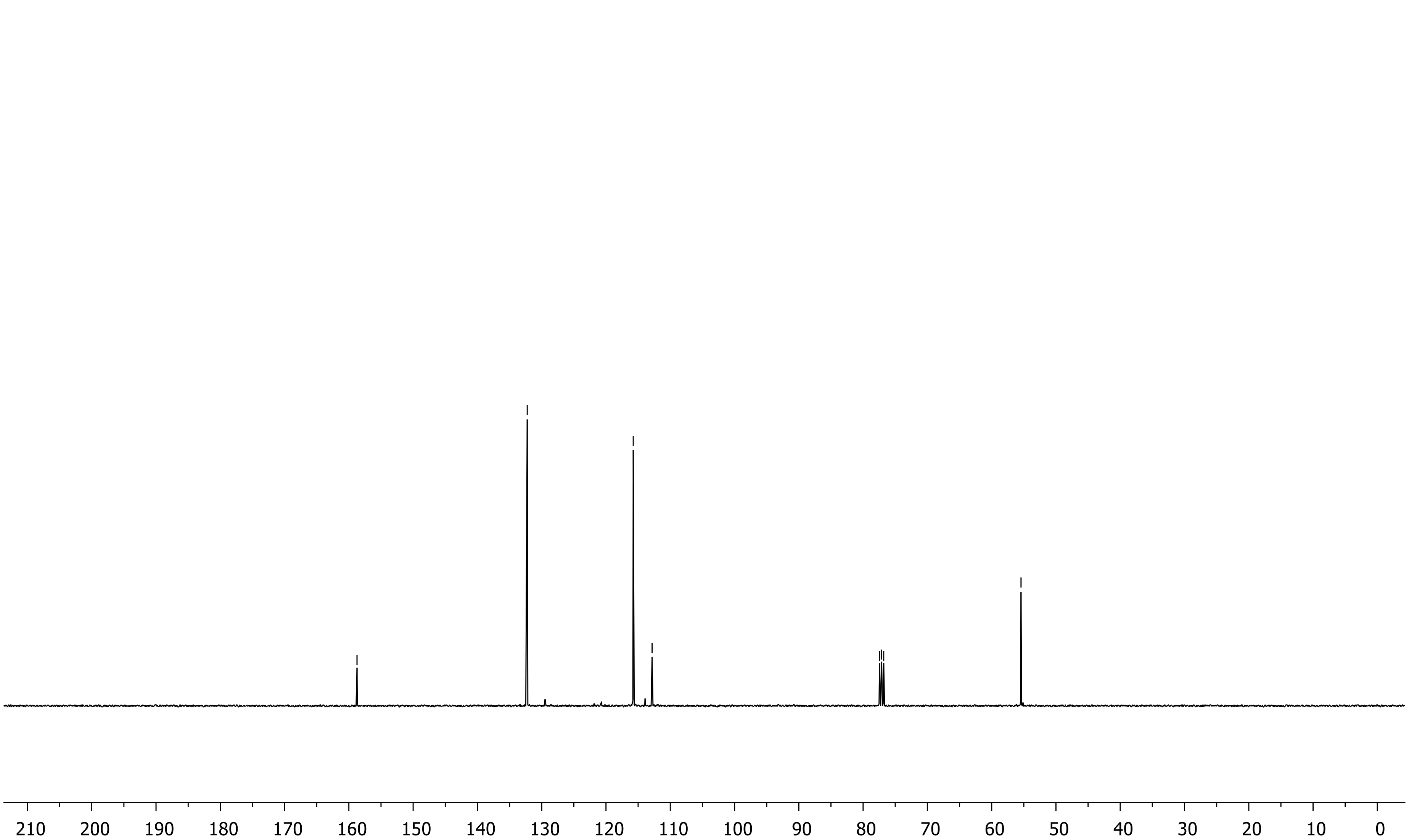

158.73
132.25
115.76
112.83
77.43
77.12
76.80
55.43
210
200
190
180
170
160
150
140
130
120
110
100
90
80
70
60
50
40
30
20
10
0

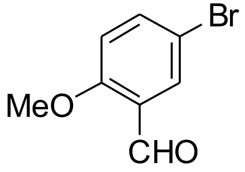
Br
MeO
CHO

2b

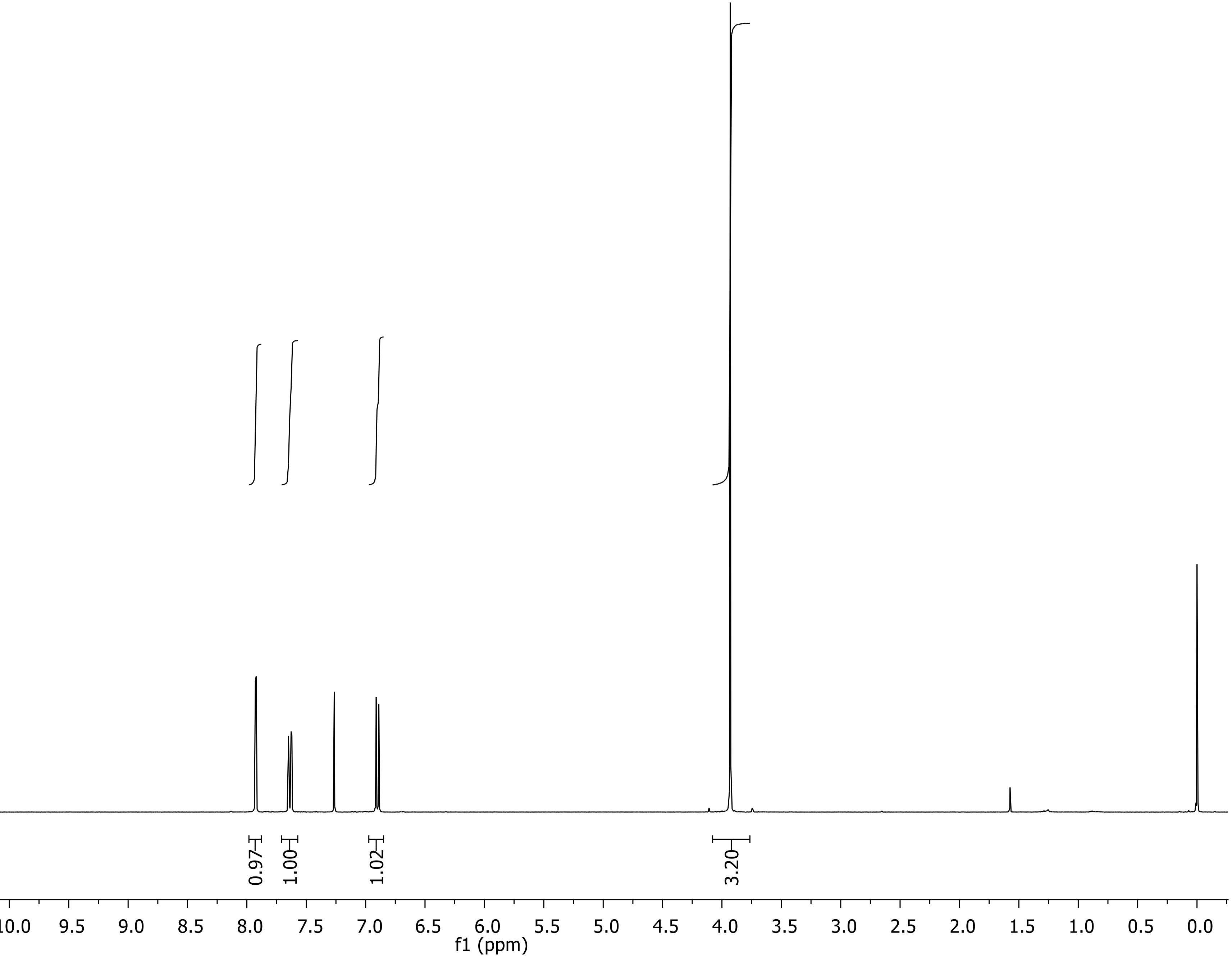
0.98
0.97
1.00
1.02
3.20
12.0 11.5 11.0 10.5 10.0 9.5 9.0 8.5 8.0 7.5 7.0 6.5 6.0 5.5 5.0 4.5 4.0 3.5 3.0 2.5 2.0 1.5 1.0 0.5 0.0
f1 (ppm)

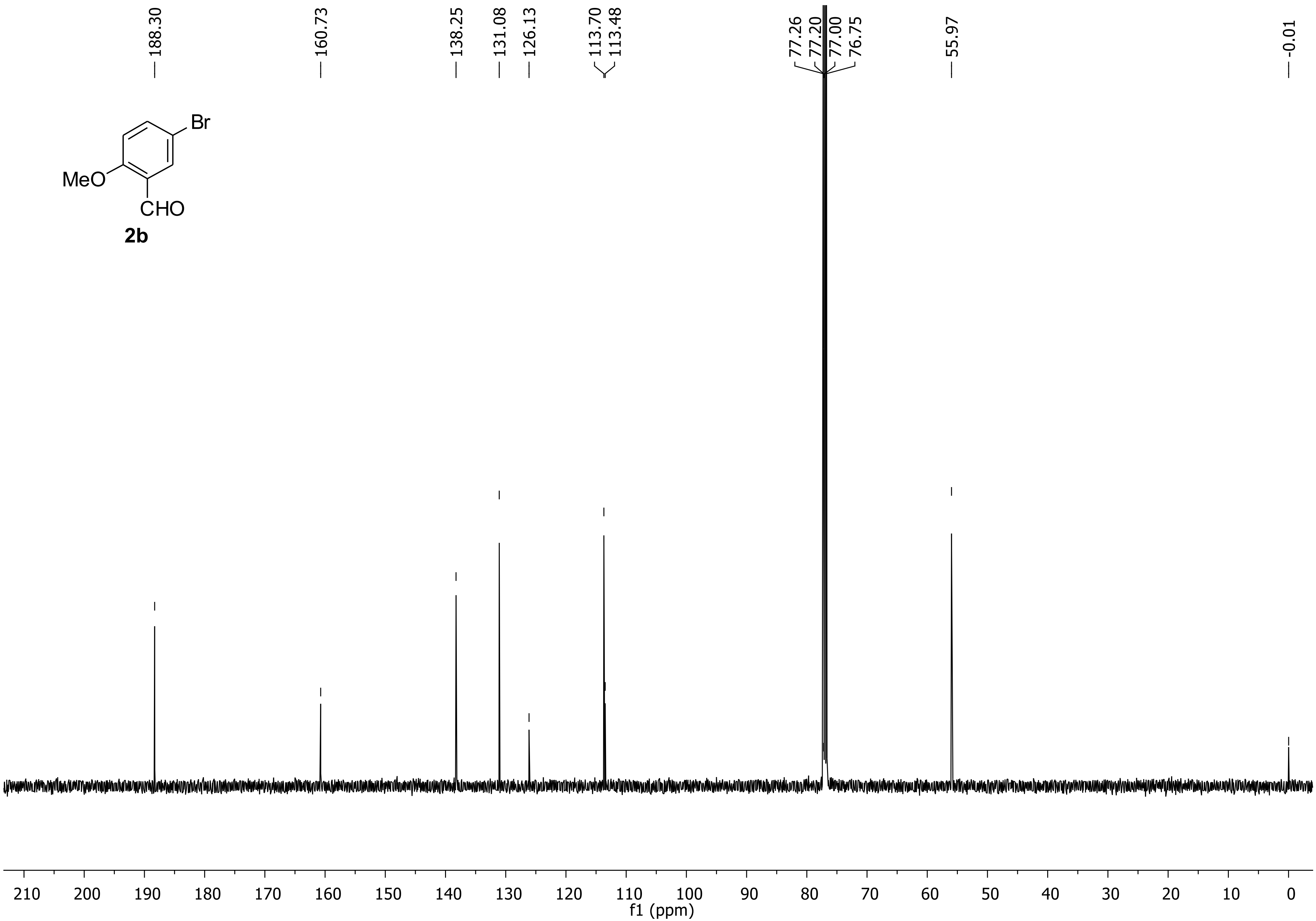
188.30
160.73
138.25
131.08
126.13
113.70
113.48
77.26
77.20
77.00
76.75
55.97
-0.01
Br
MeO
CHO
2b
f1 (ppm)

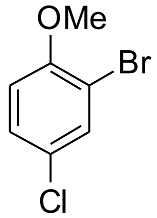

2c

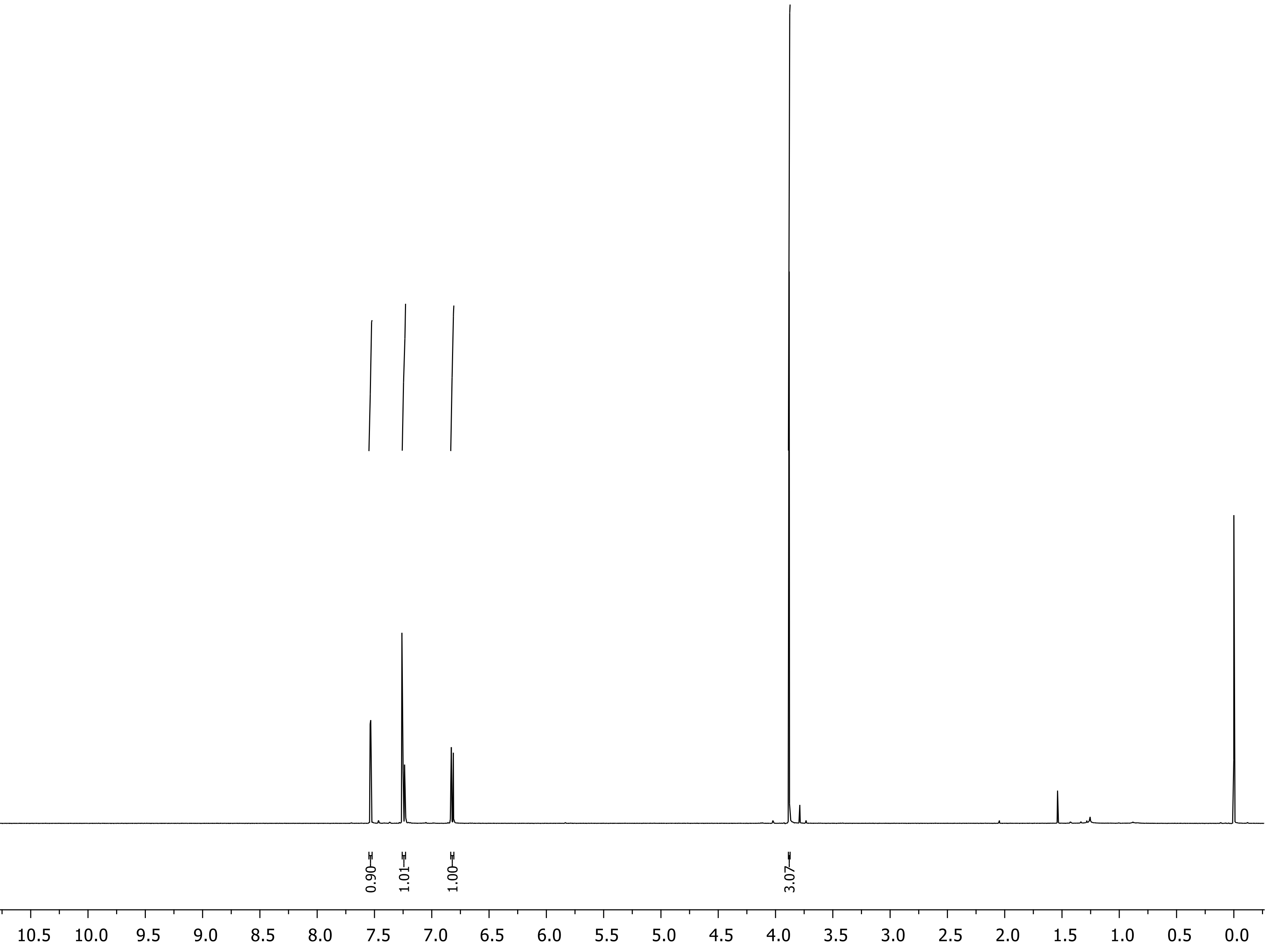

0.90
1.01
1.00
3.07
12.0
11.5
11.0
10.5
10.0
9.5
9.0
8.5
8.0
7.5
7.0
6.5
6.0
5.5
5.0
4.5
4.0
3.5
3.0
2.5
2.0
1.5
1.0
0.5
0.0

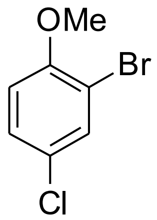
OMe
Br
Cl

2c

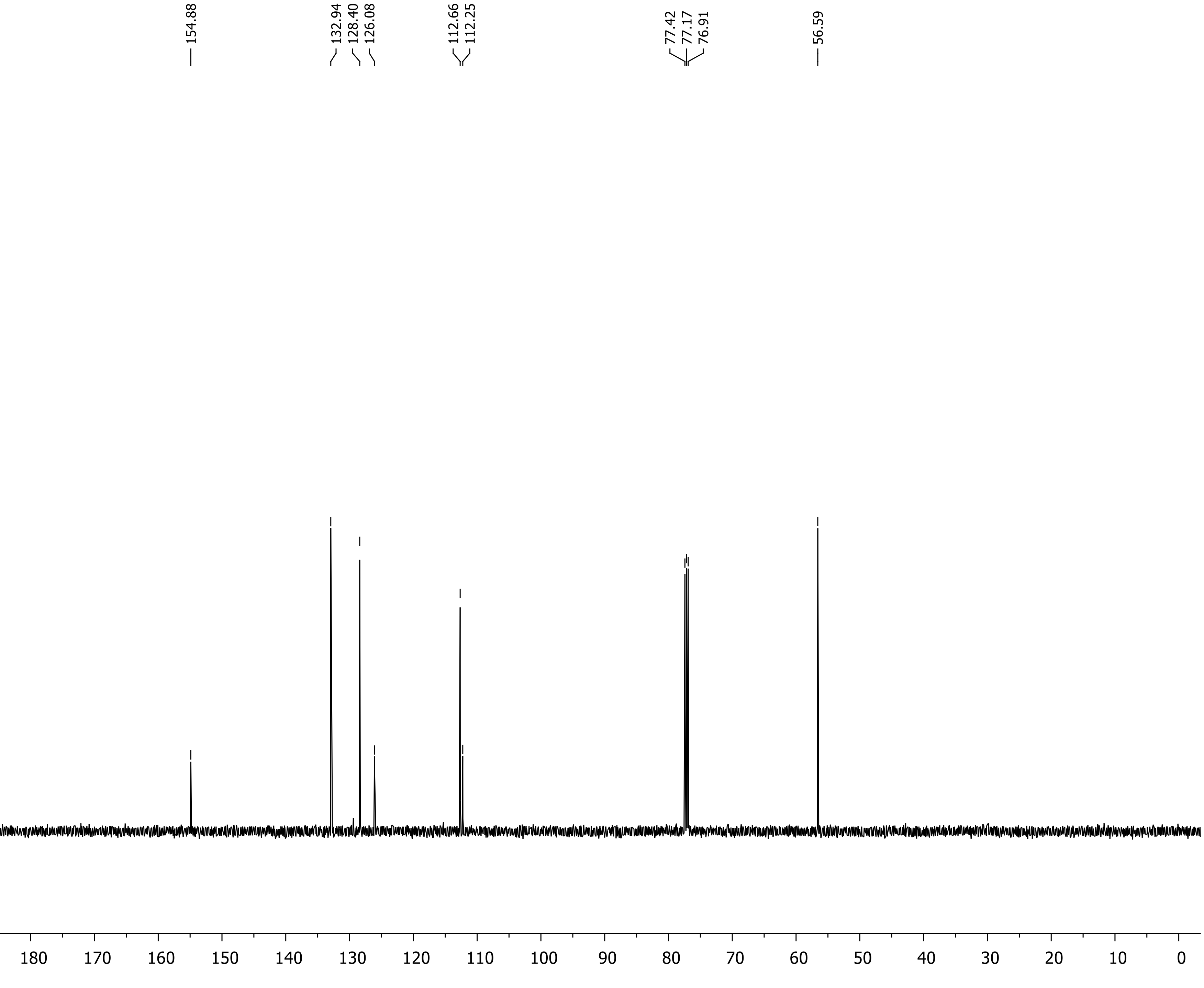
154.88
132.94
128.40
126.08
112.66
112.25
77.42
77.17
76.91
56.59
210 200 190 180 170 160 150 140 130 120 110 100 90 80 70 60 50 40 30 20 10 0

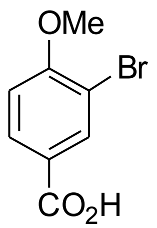
OMe
Br
CO_2H

2d

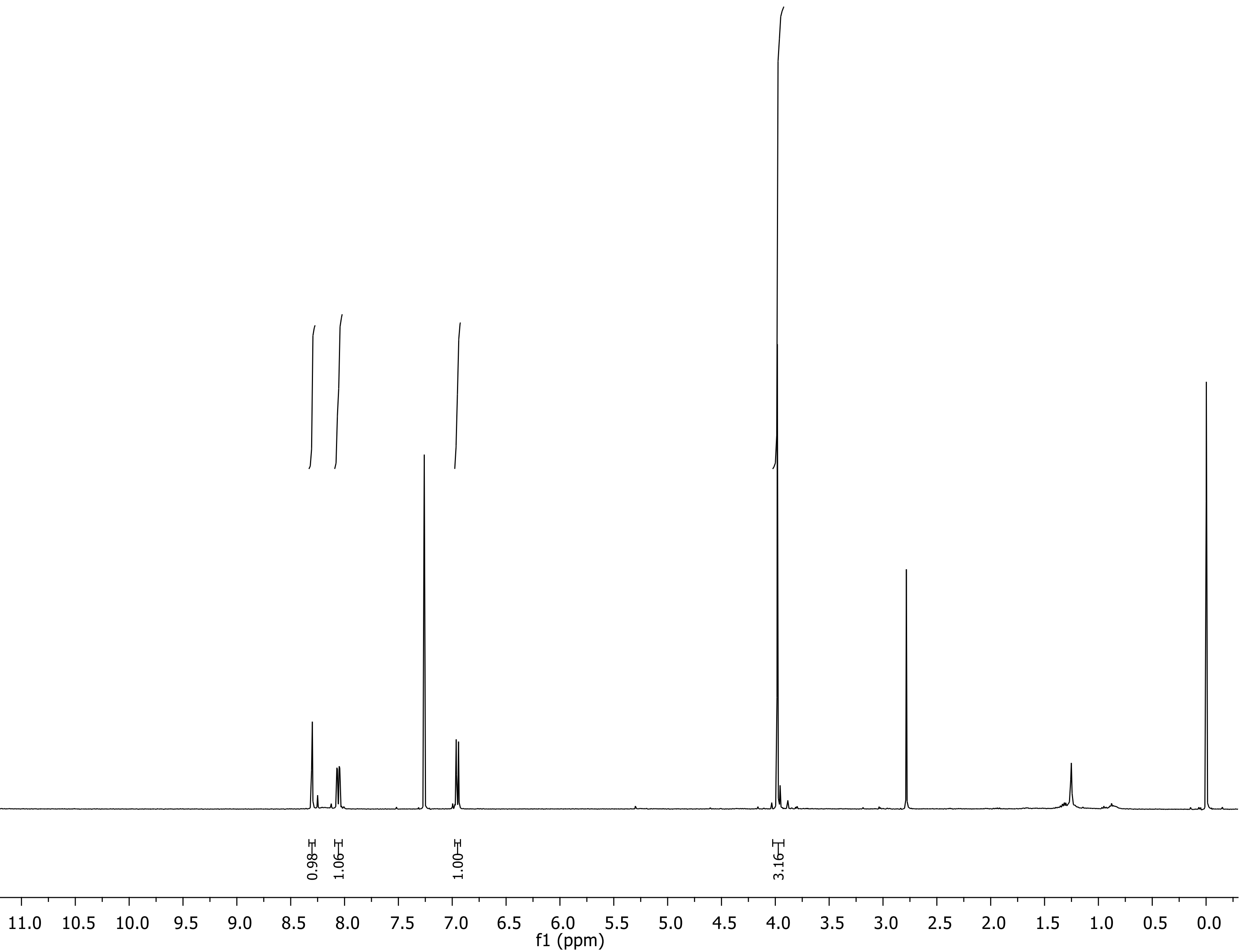
0.98
1.06
1.00
3.16
12.0 11.5 11.0 10.5 10.0 9.5 9.0 8.5 8.0 7.5 7.0 6.5 6.0 5.5 5.0 4.5 4.0 3.5 3.0 2.5 2.0 1.5 1.0 0.5 0.0
f1 (ppm)

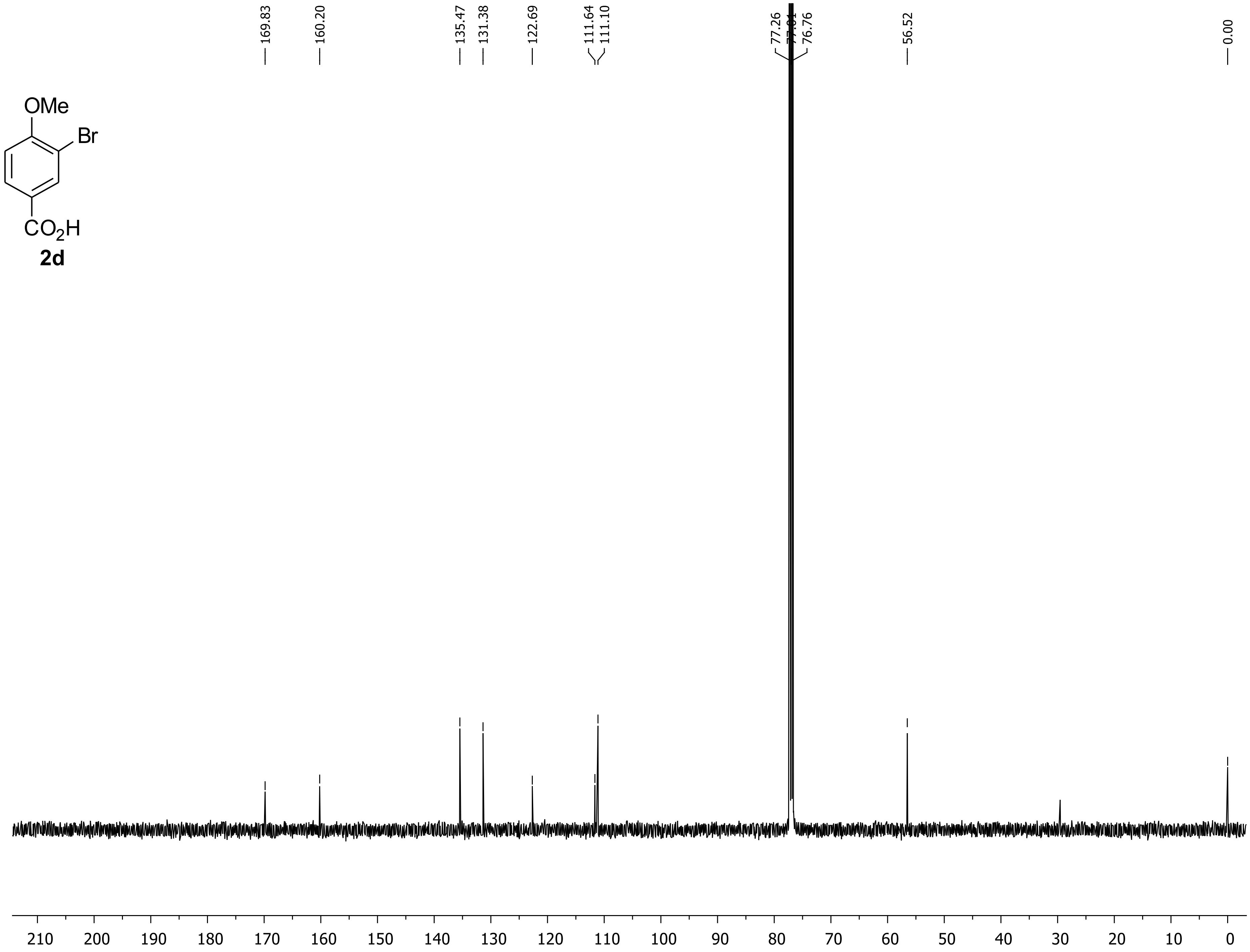

169.83
160.20
135.47
131.38
122.69
111.64
111.10
77.26
76.76
56.52
0.00
OMe
Br
CO2H
210
200
190
180
170
160
150
140
130
120
110
100
90
80
70
60
50
40
30
20
10
0

2d

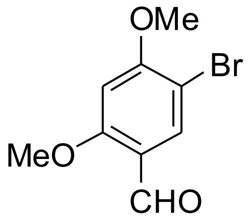
OMe
Br
MeO
CHO

2e

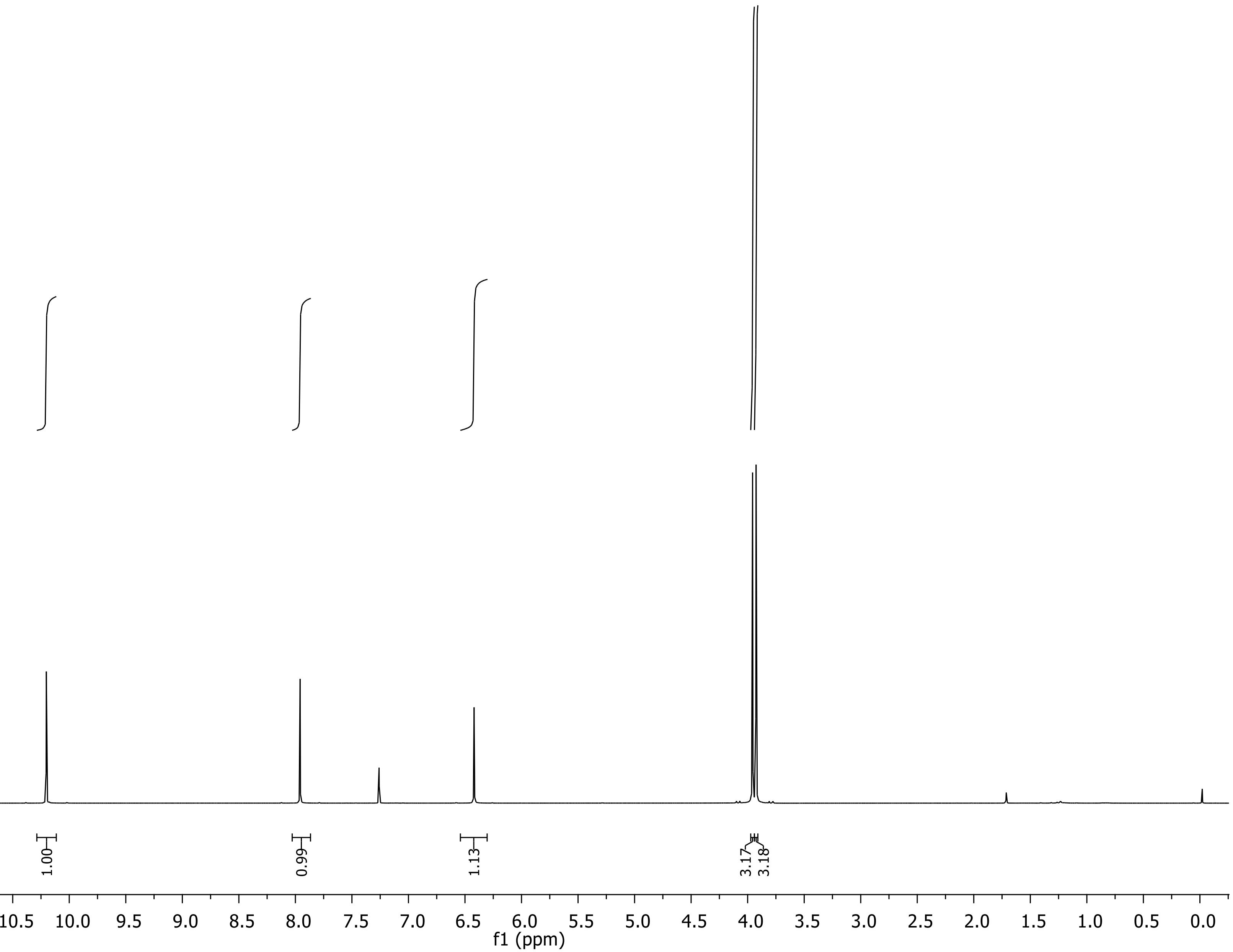
1.00
0.99
1.13
3.17
3.18
12.0 11.5 11.0 10.5 10.0 9.5 9.0 8.5 8.0 7.5 7.0 6.5 6.0 5.5 5.0 4.5 4.0 3.5 3.0 2.5 2.0 1.5 1.0 0.5 0.0
f1 (ppm)

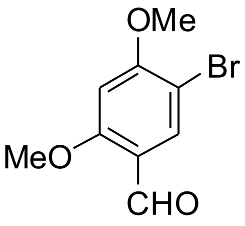

2e

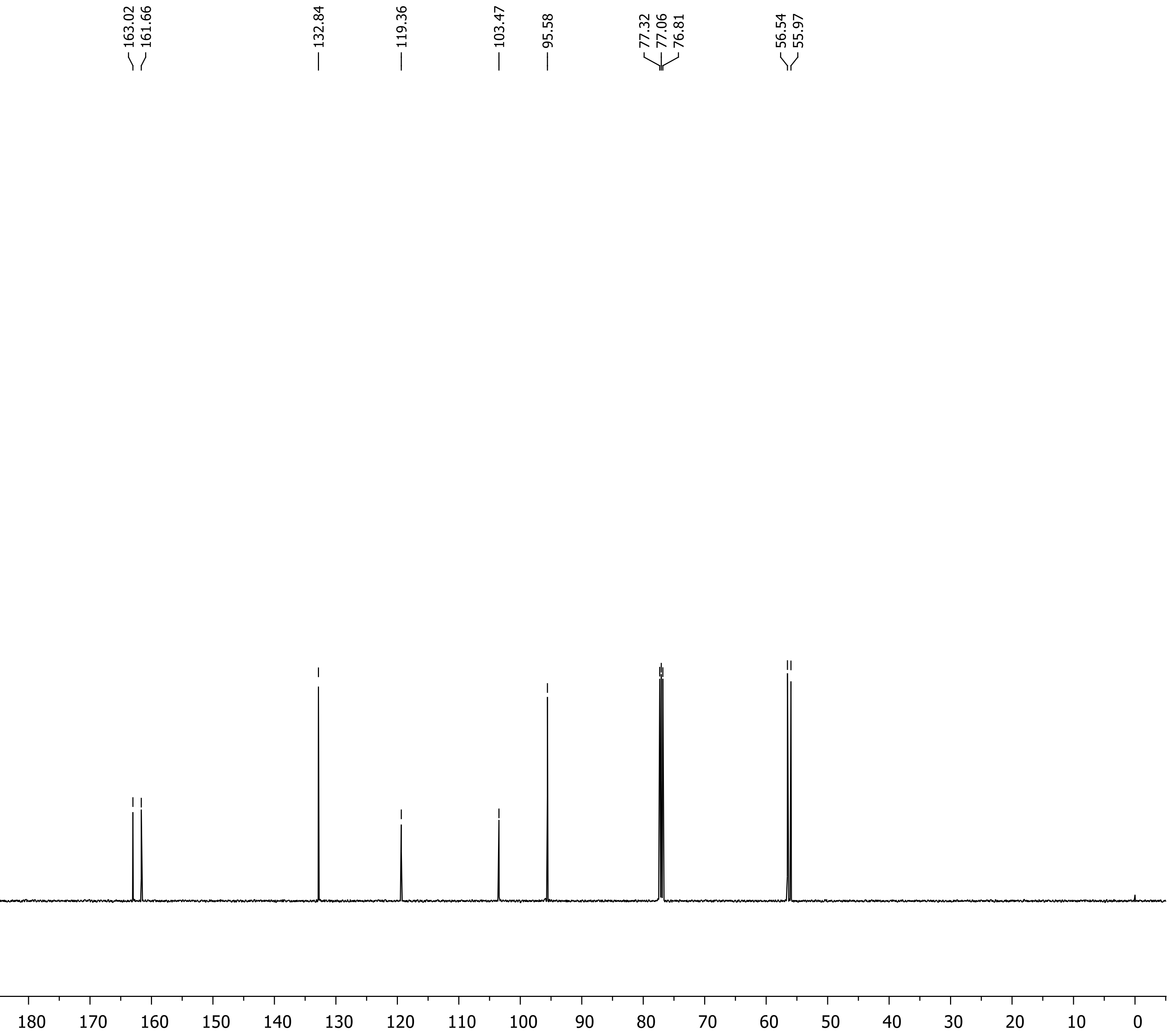
187.06
163.02
161.66
132.84
119.36
103.47
95.58
77.32
77.06
76.81
56.54
55.97
210 200 190 180 170 160 150 140 130 120 110 100 90 80 70 60 50 40 30 20 10 0

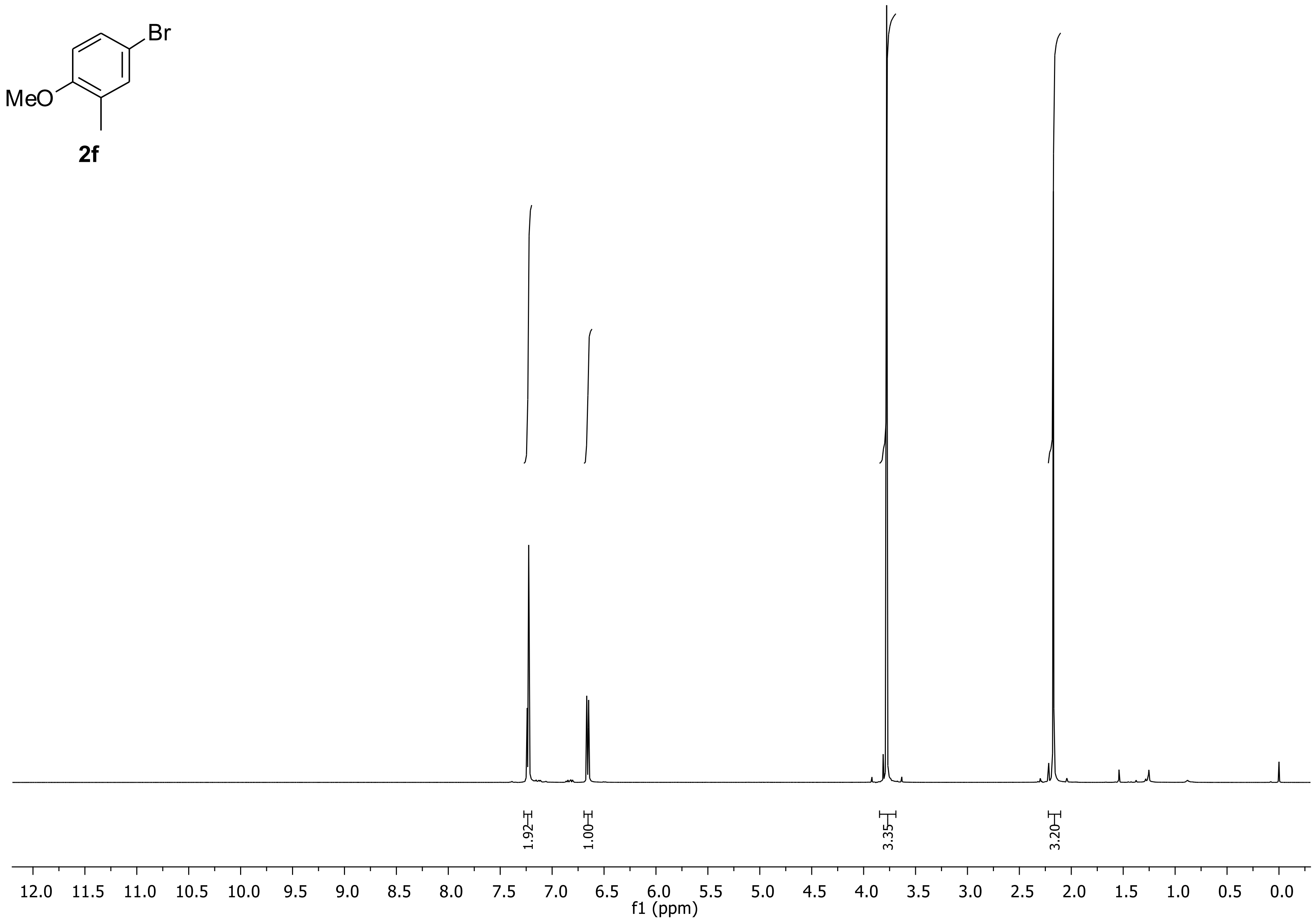

Br
MeO
2f
1.92
1.00
3.35
3.20
12.0 11.5 11.0 10.5 10.0 9.5 9.0 8.5 8.0 7.5 7.0 6.5 6.0 5.5 5.0 4.5 4.0 3.5 3.0 2.5 2.0 1.5 1.0 0.5 0.0
f1 (ppm)

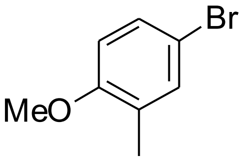

2f

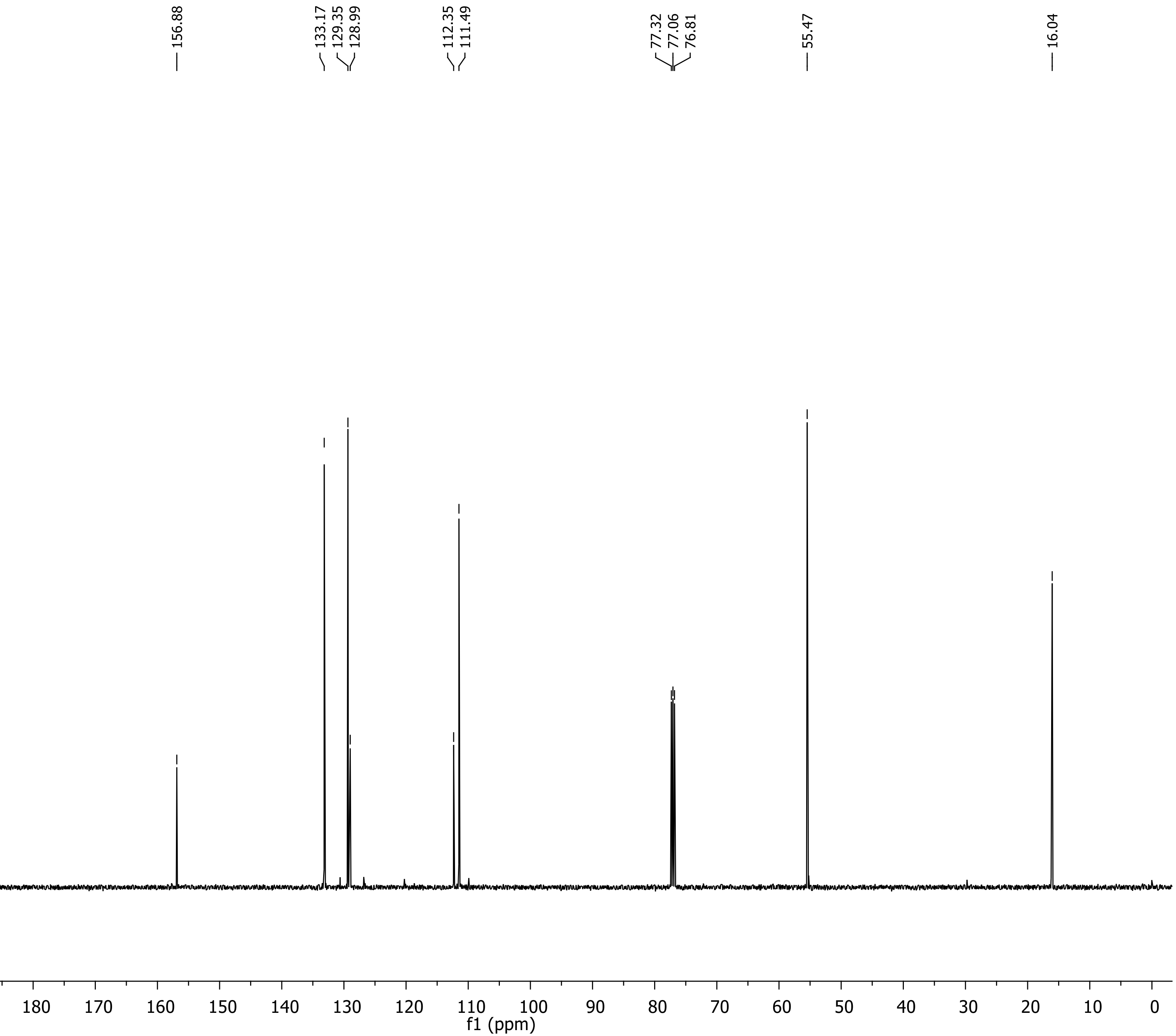

156.88
133.17
129.35
128.99
112.35
111.49
77.32
77.06
76.81
55.47
16.04
210
200
190
180
170
160
150
140
130
120
110
100
90
80
70
60
50
40
30
20
10
0
f1 (ppm)

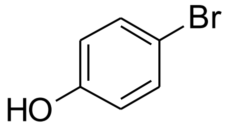

2g

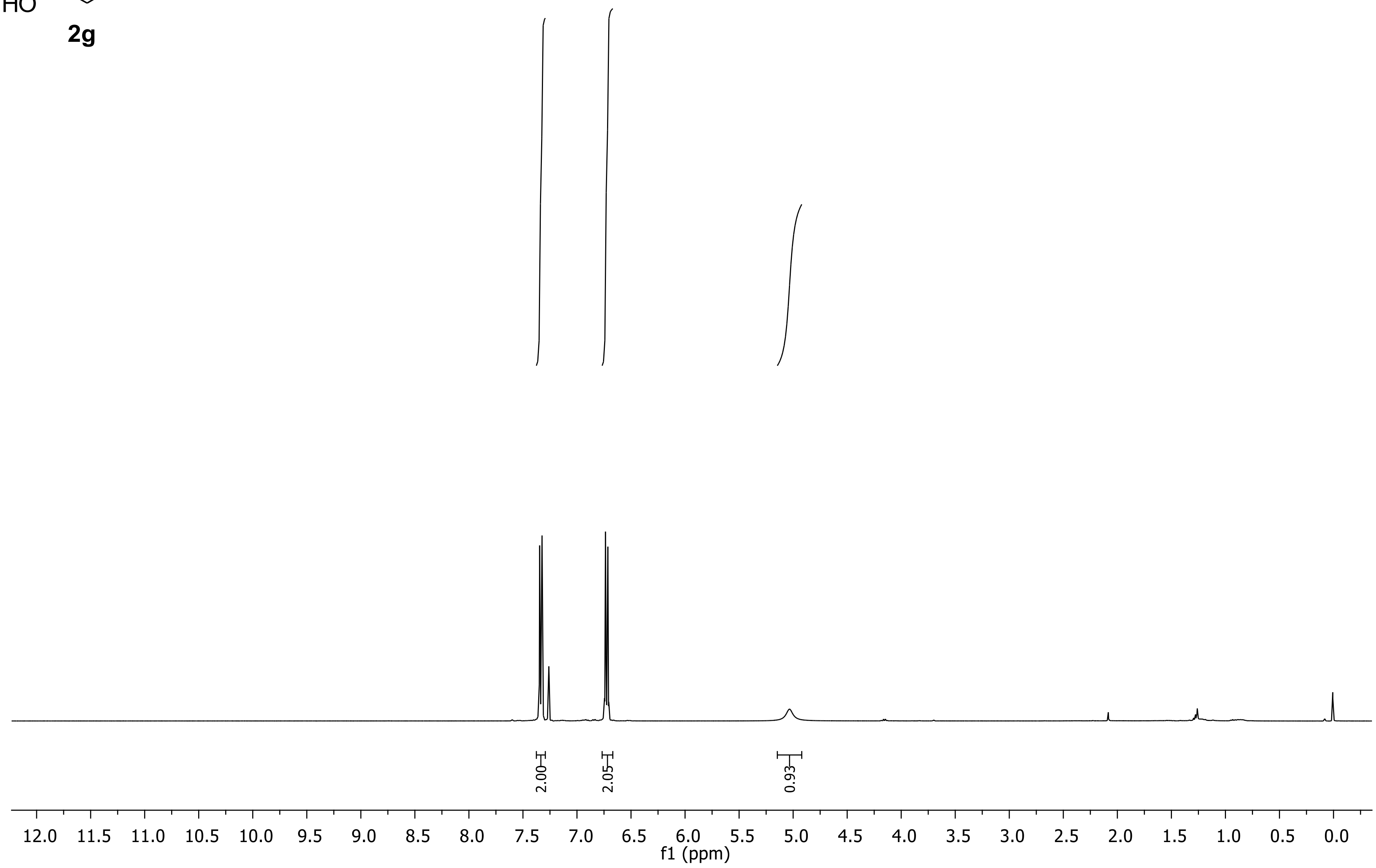
2.00
2.05
0.93
12.0 11.5 11.0 10.5 10.0 9.5 9.0 8.5 8.0 7.5 7.0 6.5 6.0 5.5 5.0 4.5 4.0 3.5 3.0 2.5 2.0 1.5 1.0 0.5 0.0
f1 (ppm)

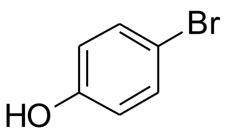

2g

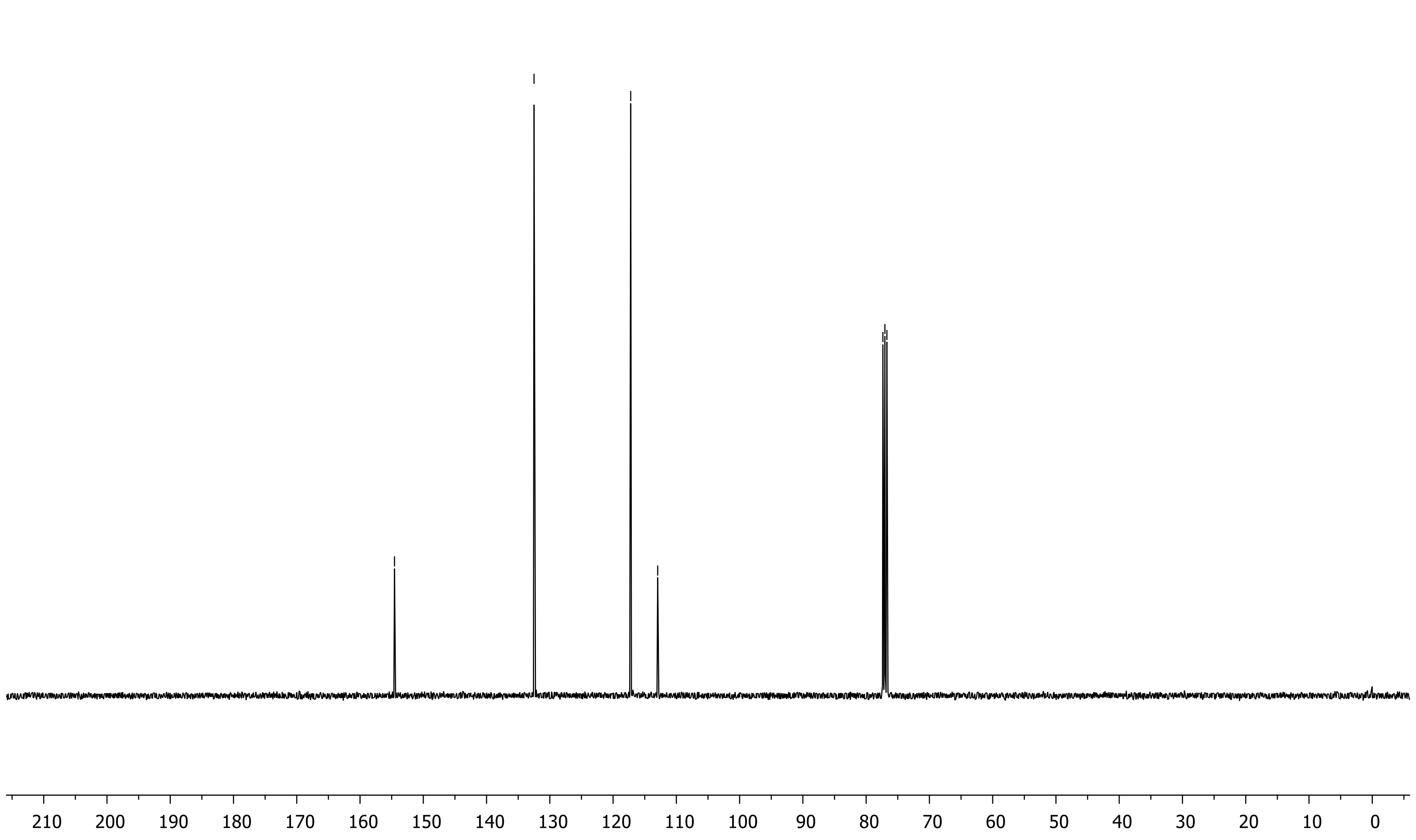

154.55
132.50
117.22
112.95
77.35
77.04
76.72
210 200 190 180 170 160 150 140 130 120 110 100 90 80 70 60 50 40 30 20 10 0

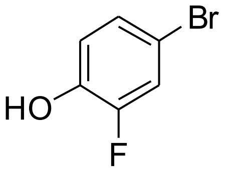

Br
HO
F

2h

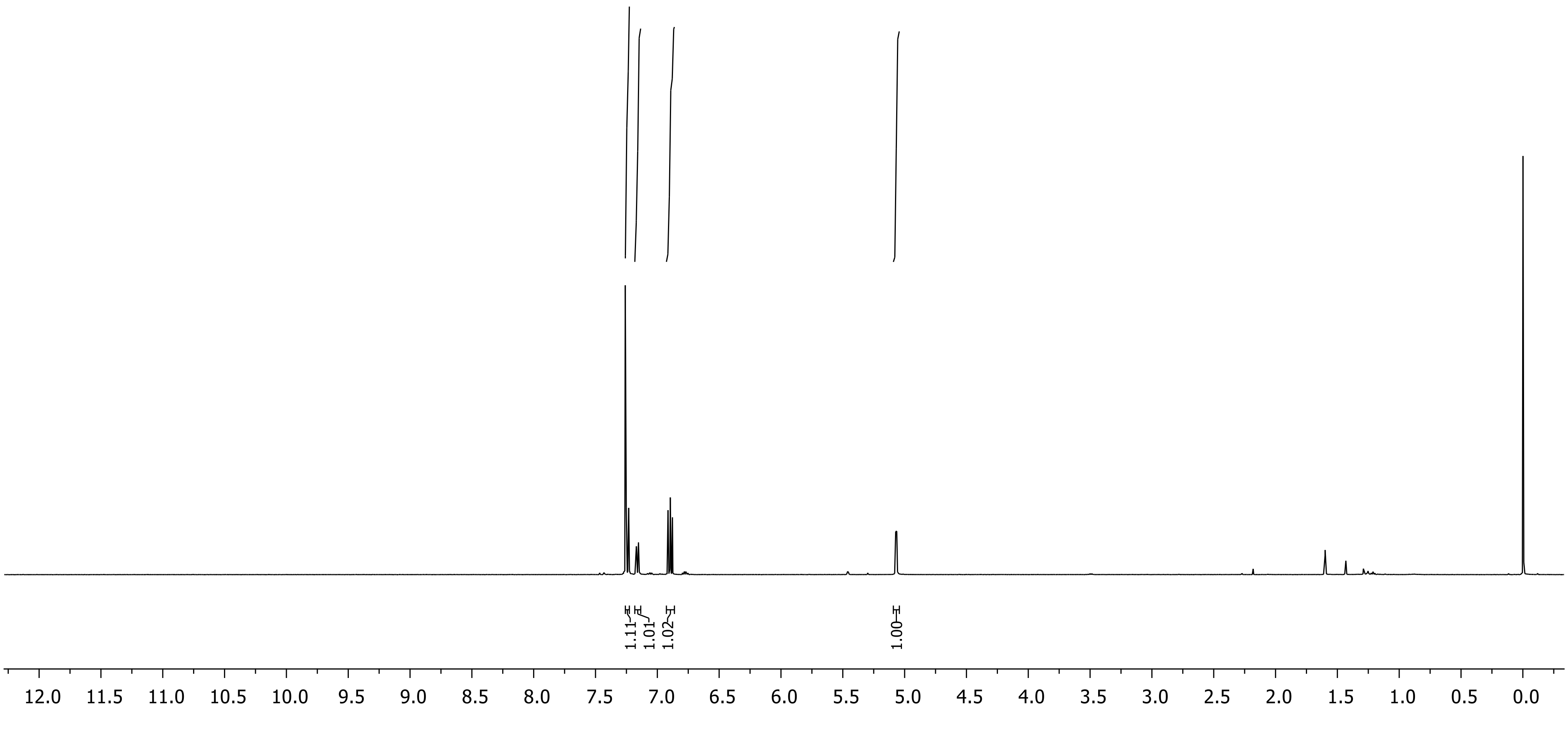

1.11
1.01
1.02
1.00
12.0 11.5 11.0 10.5 10.0 9.5 9.0 8.5 8.0 7.5 7.0 6.5 6.0 5.5 5.0 4.5 4.0 3.5 3.0 2.5 2.0 1.5 1.0 0.5 0.0

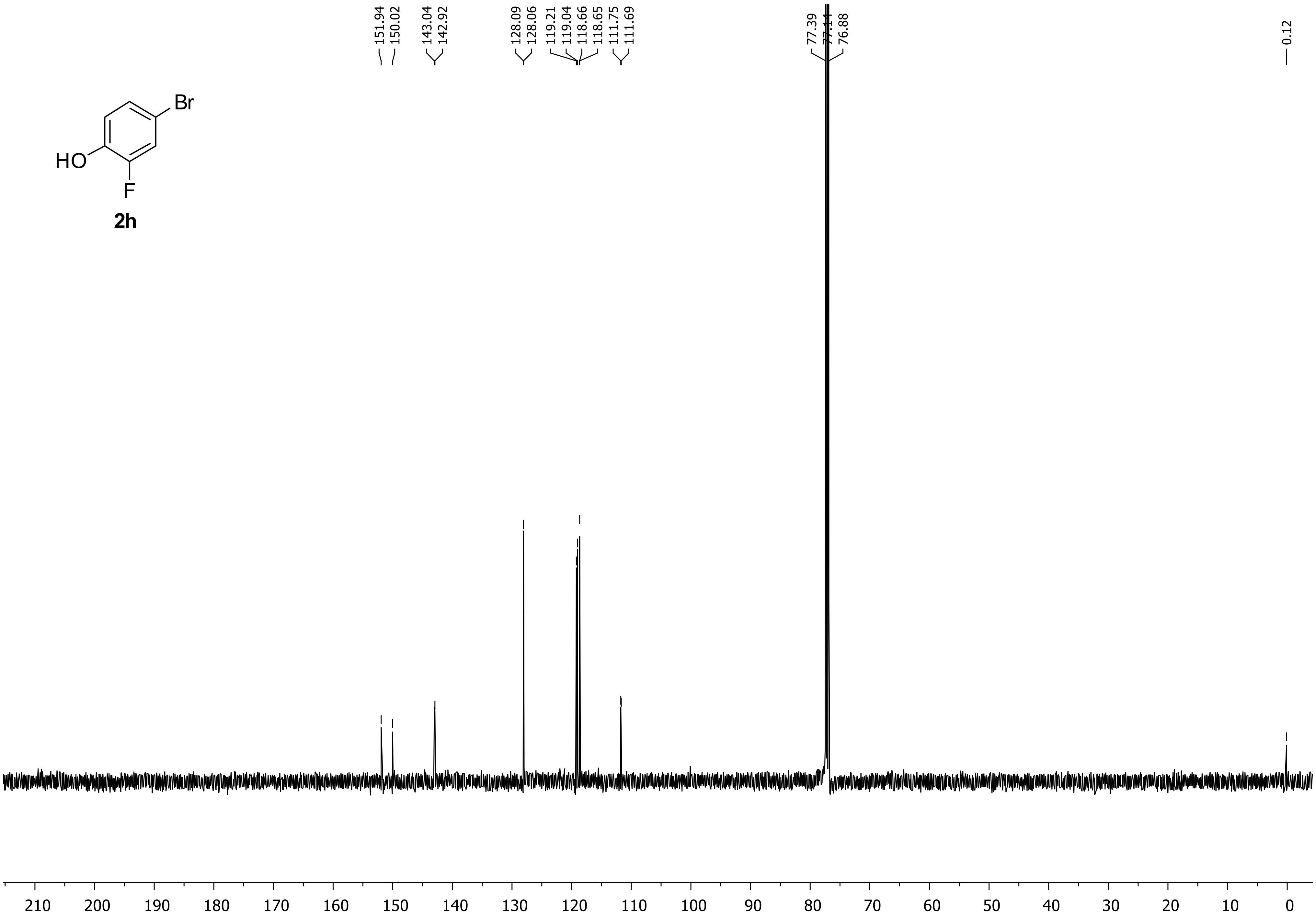

151.94
150.02
143.04
142.92
128.09
128.06
119.21
119.04
118.66
118.65
111.75
111.69
77.39
76.88
0.12
Br
HO
F
2h
210
200
190
180
170
160
150
140
130
120
110
100
90
80
70
60
50
40
30
20
10
0

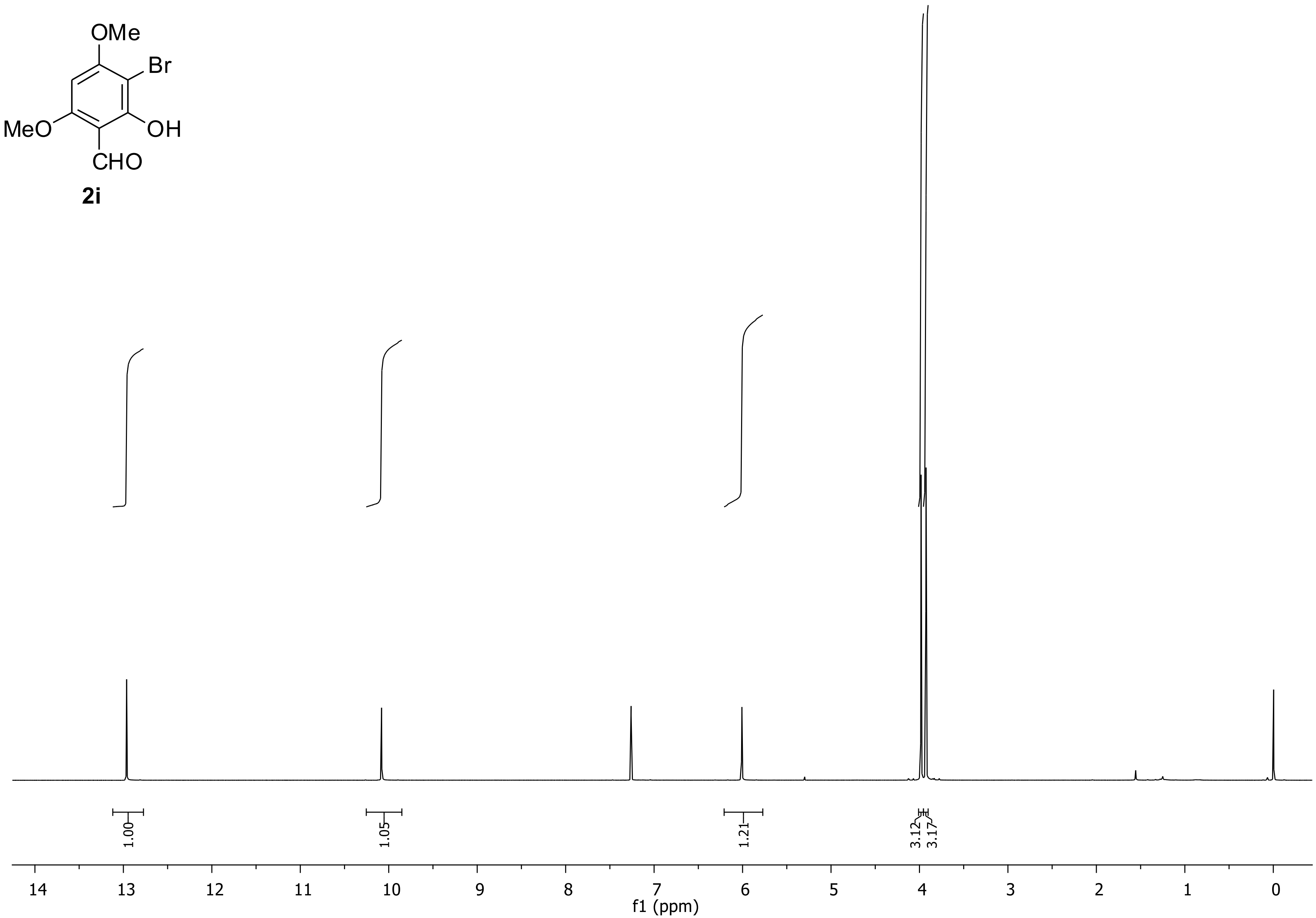
OMe
Br
MeO
OH
CHO
2i
1.00
1.05
1.21
3.12
3.17
14 13 12 11 10 9 8 7 6 5 4 3 2 1 0
f1 (ppm)

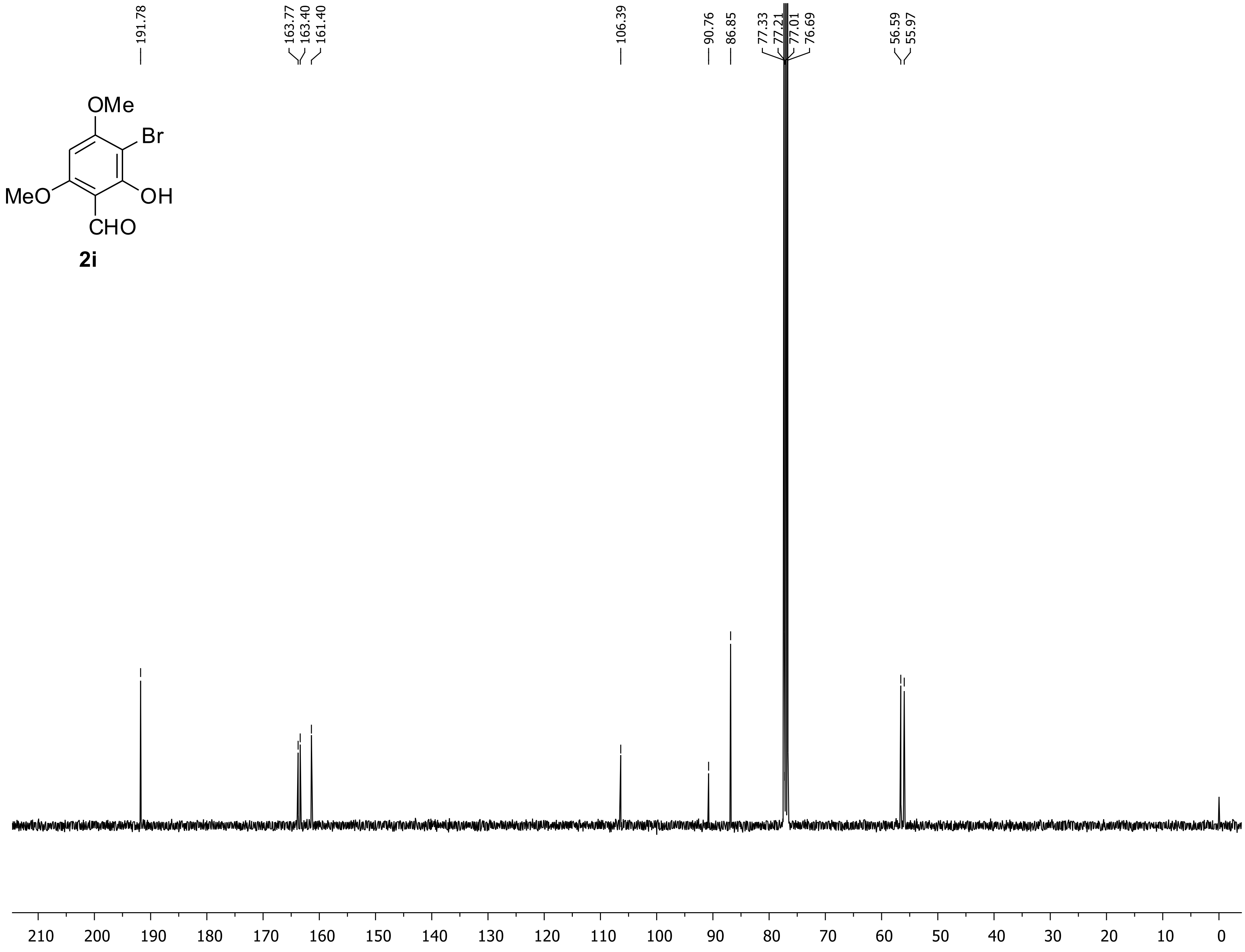
191.78
163.77
163.40
161.40
106.39
90.76
86.85
77.33
77.21
77.01
76.69
56.59
55.97
OMe
Br
MeO
OH
CHO
2i
210 200 190 180 170 160 150 140 130 120 110 100 90 80 70 60 50 40 30 20 10 0

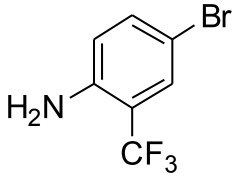
Br
H2N
CF3

2j

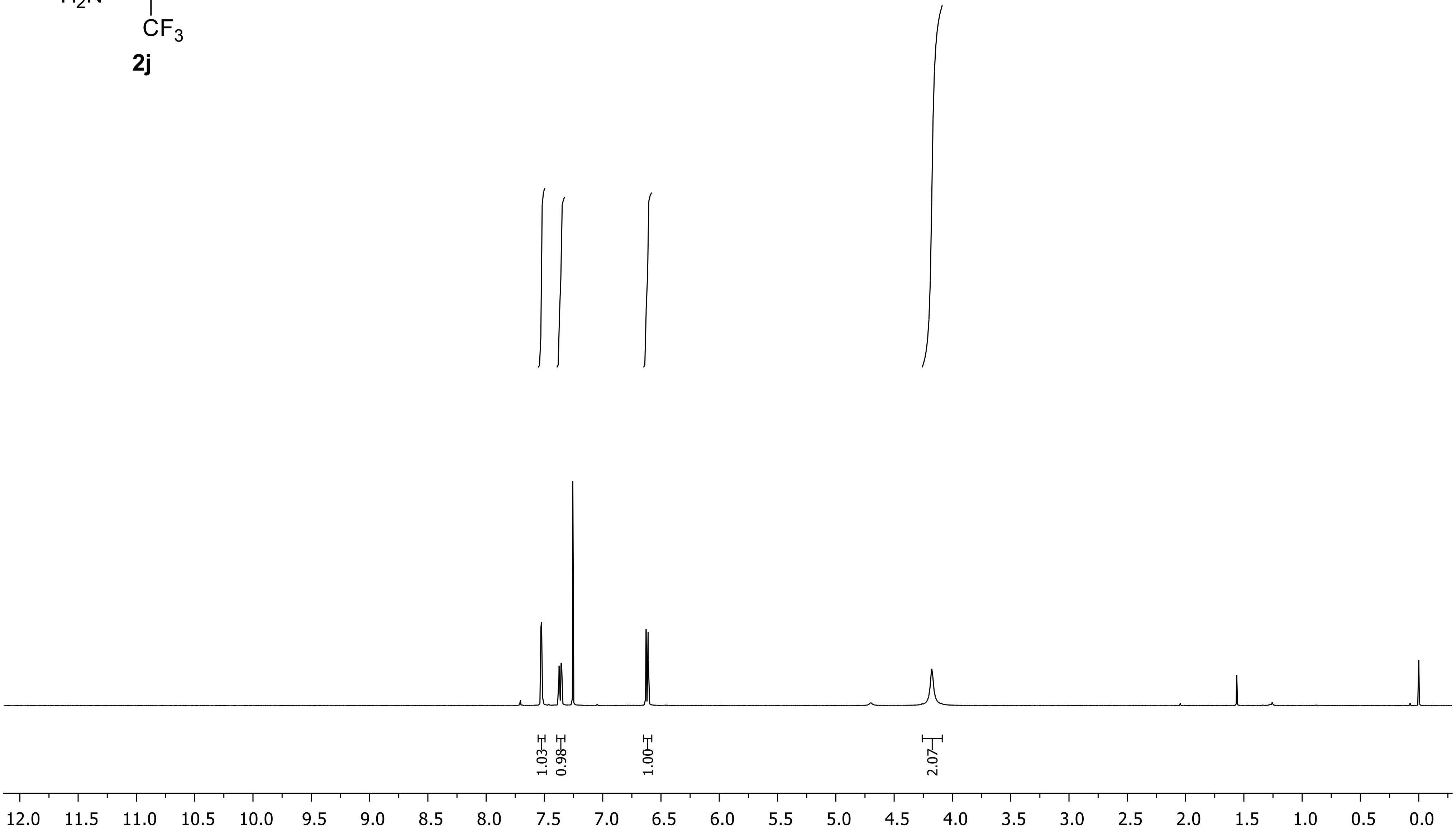
1.03
0.98
1.00
2.07
12.0 11.5 11.0 10.5 10.0 9.5 9.0 8.5 8.0 7.5 7.0 6.5 6.0 5.5 5.0 4.5 4.0 3.5 3.0 2.5 2.0 1.5 1.0 0.5 0.0

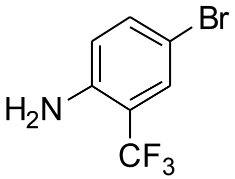
Br
H2N
CF3

2j

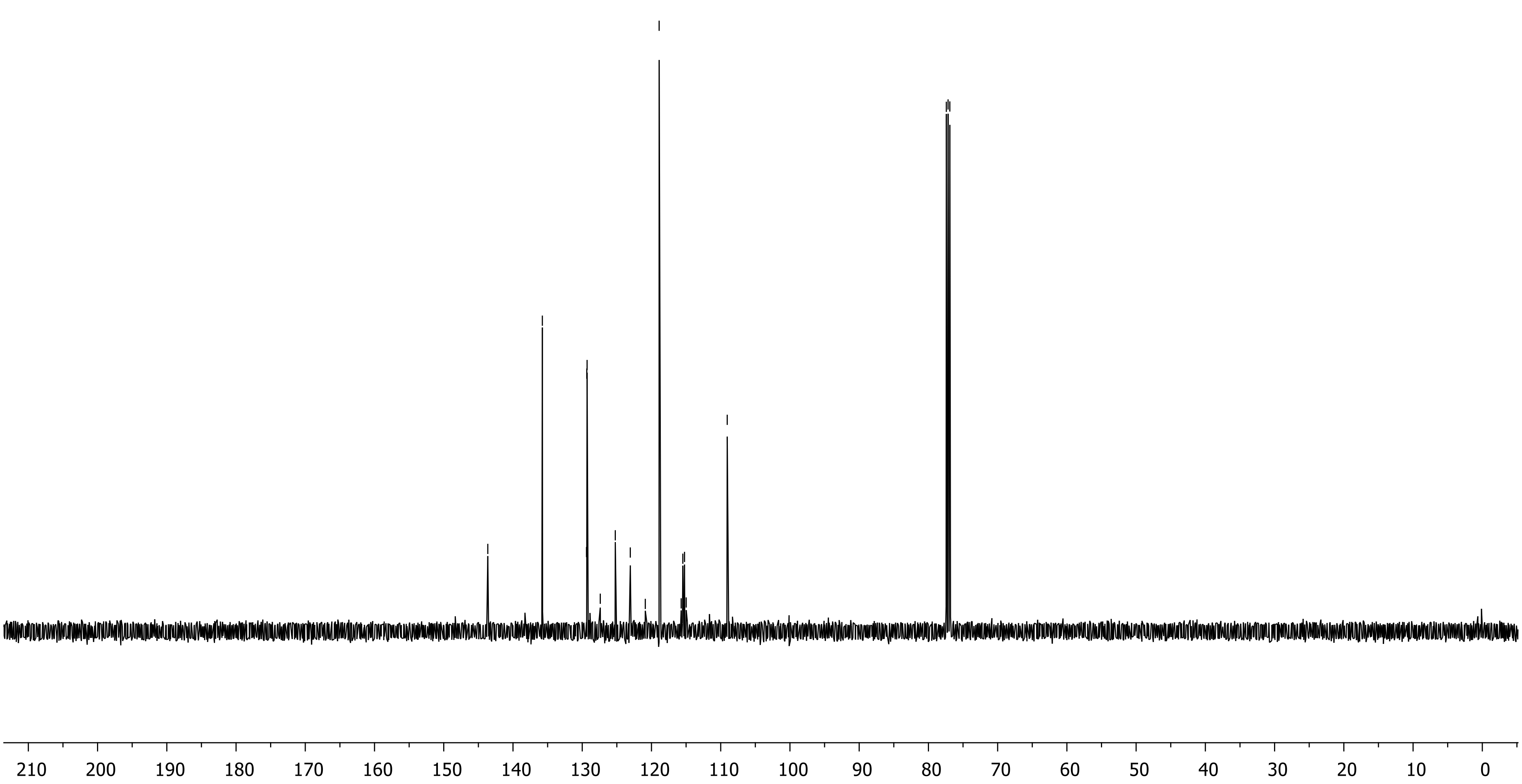
143.64
135.76
129.39
129.34
129.30
129.26
125.23
123.06
118.89
115.47
115.23
109.06
77.41
77.16
76.90
210
200
190
180
170
160
150
140
130
120
110
100
90
80
70
60
50
40
30
20
10
0

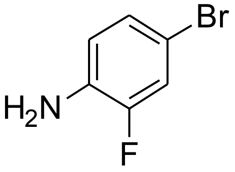

Br
H2N
F

2k

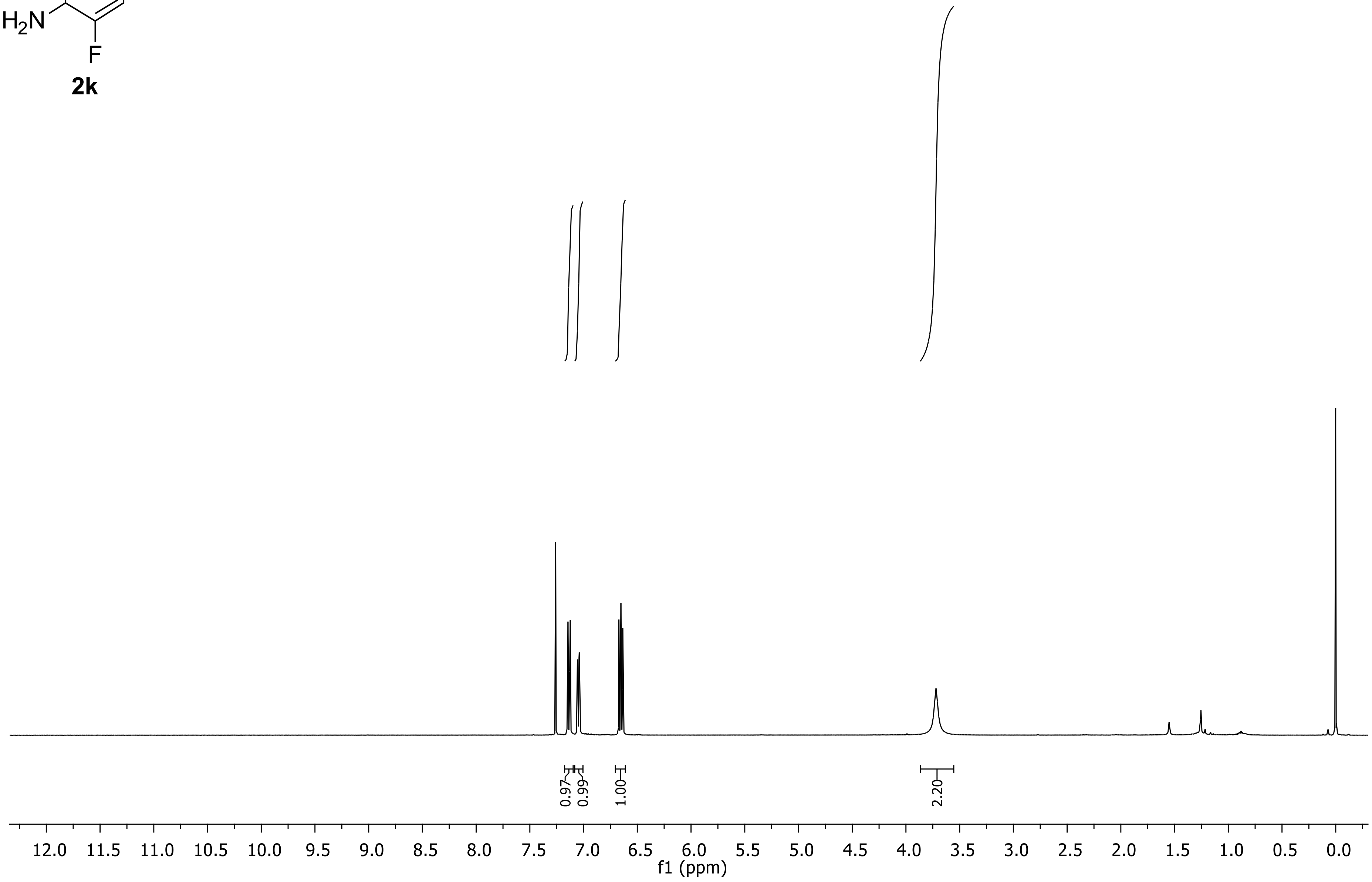

0.97
0.99
1.00
2.20
12.0
11.5
11.0
10.5
10.0
9.5
9.0
8.5
8.0
7.5
7.0
6.5
6.0
5.5
5.0
4.5
4.0
3.5
3.0
2.5
2.0
1.5
1.0
0.5
0.0
f1 (ppm)

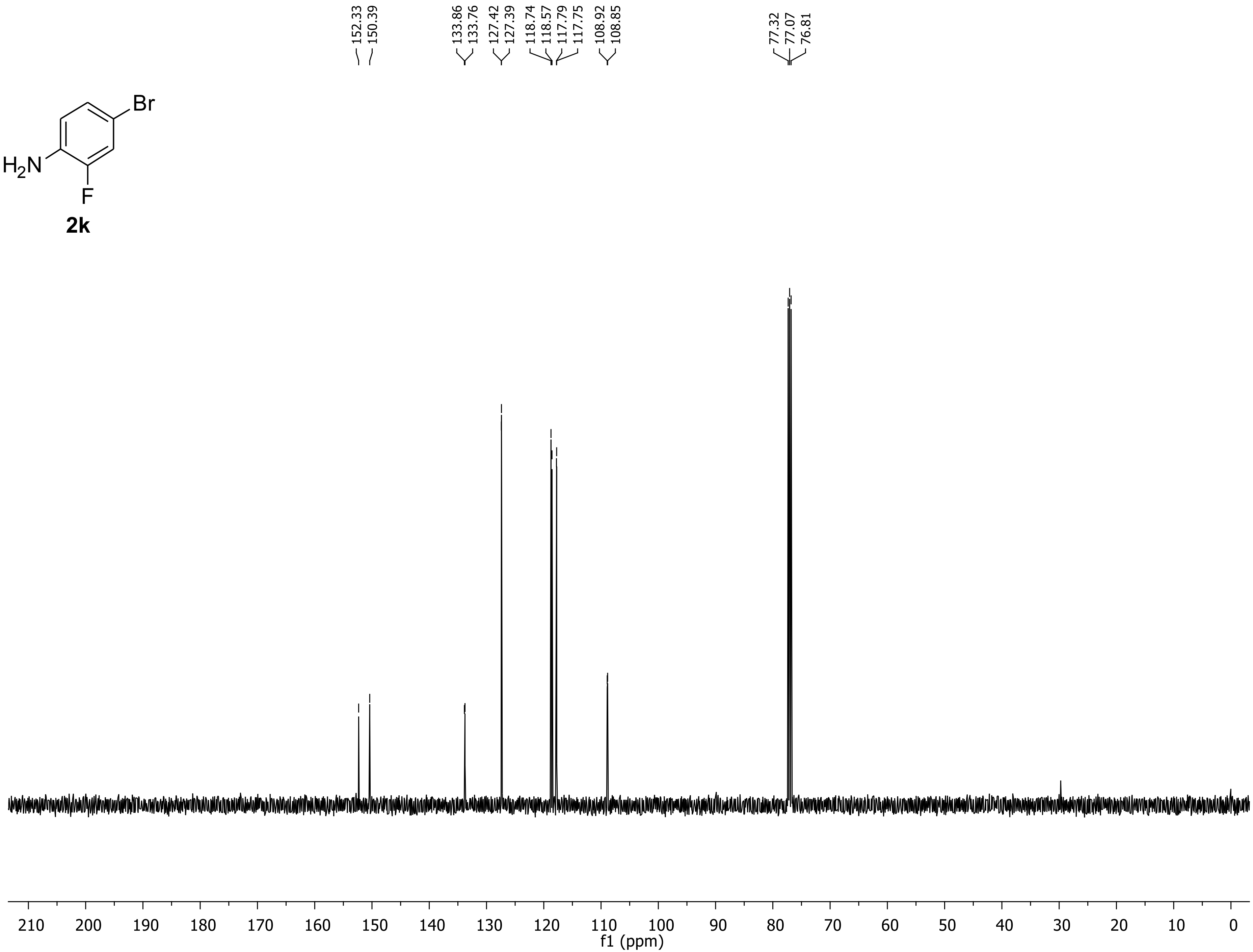
152.33
150.39
133.86
133.76
127.42
127.39
118.74
118.57
117.79
117.75
108.92
108.85
77.32
77.07
76.81
Br
H2N
F
2k
210 200 190 180 170 160 150 140 130 120 110 100 90 80 70 60 50 40 30 20 10 0
f1 (ppm)

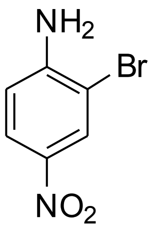
NH2
Br
NO2

2l

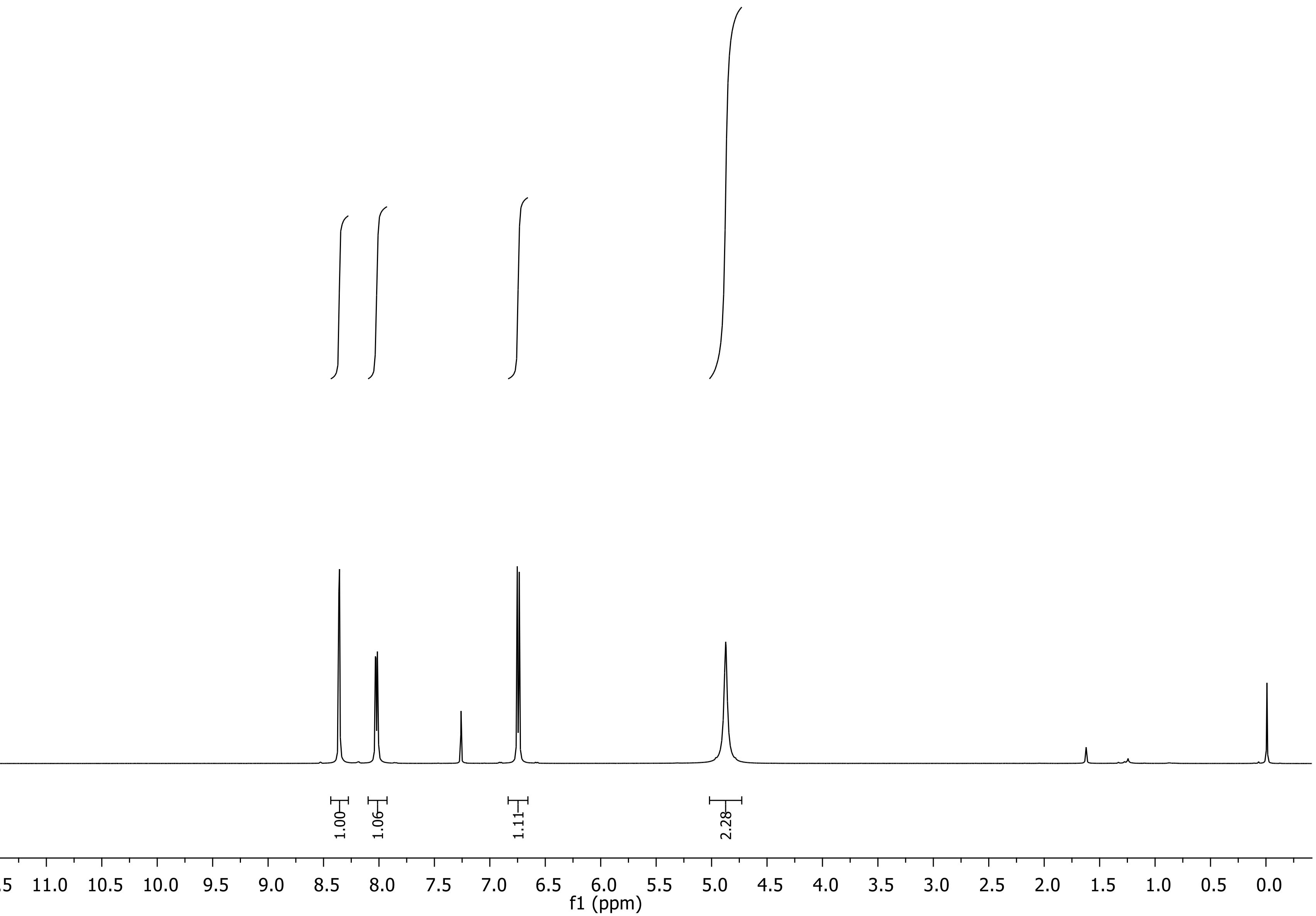
1.00
1.06
1.11
2.28
12.0 11.5 11.0 10.5 10.0 9.5 9.0 8.5 8.0 7.5 7.0 6.5 6.0 5.5 5.0 4.5 4.0 3.5 3.0 2.5 2.0 1.5 1.0 0.5 0.0
f1 (ppm)

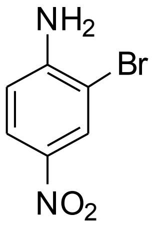

NH2
Br
NO2

2l

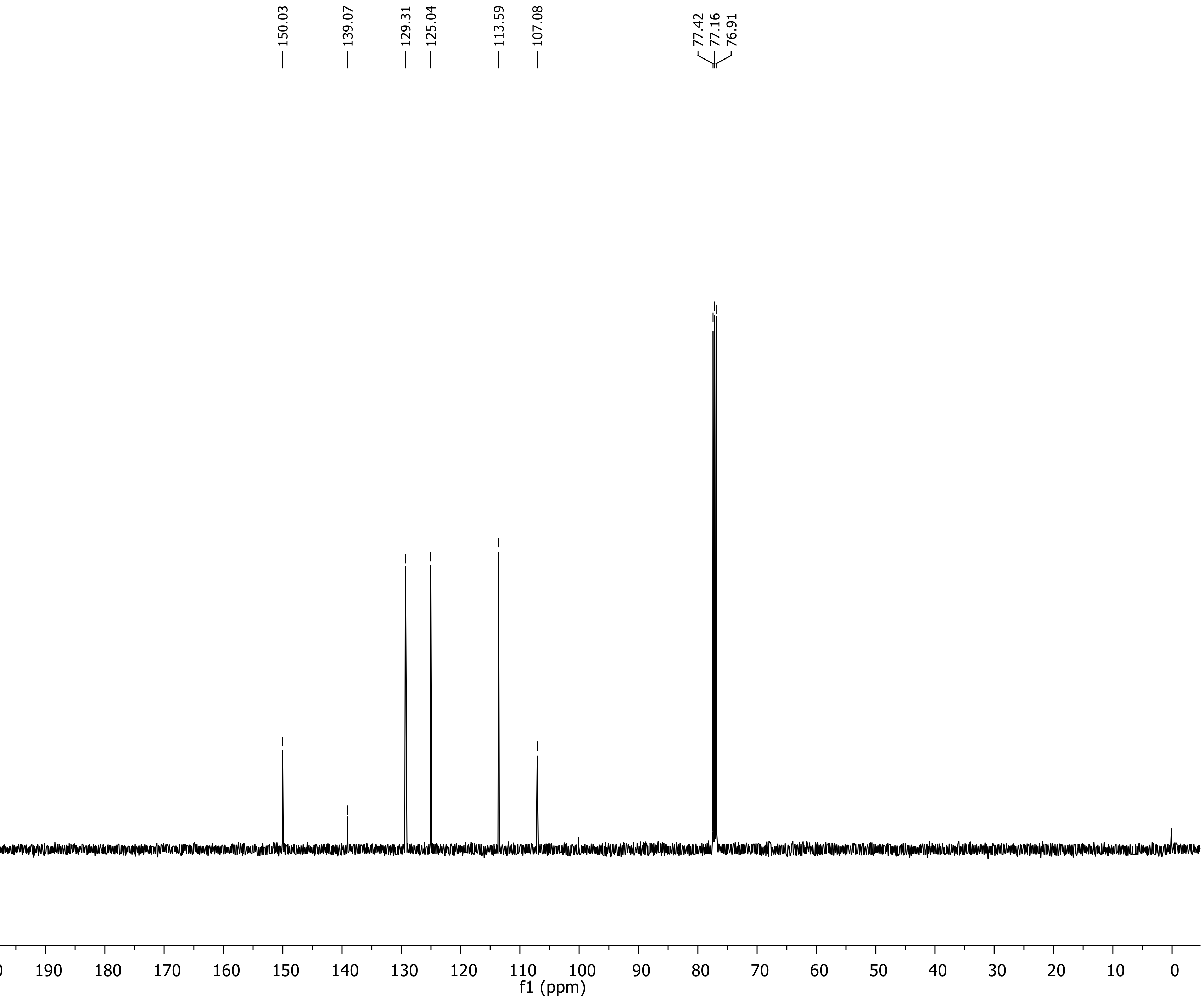

150.03
139.07
129.31
125.04
113.59
107.08
77.42
77.16
76.91
210 200 190 180 170 160 150 140 130 120 110 100 90 80 70 60 50 40 30 20 10 0
f1 (ppm)

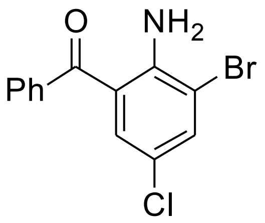
O
NH2
Br
Ph
Cl

2m

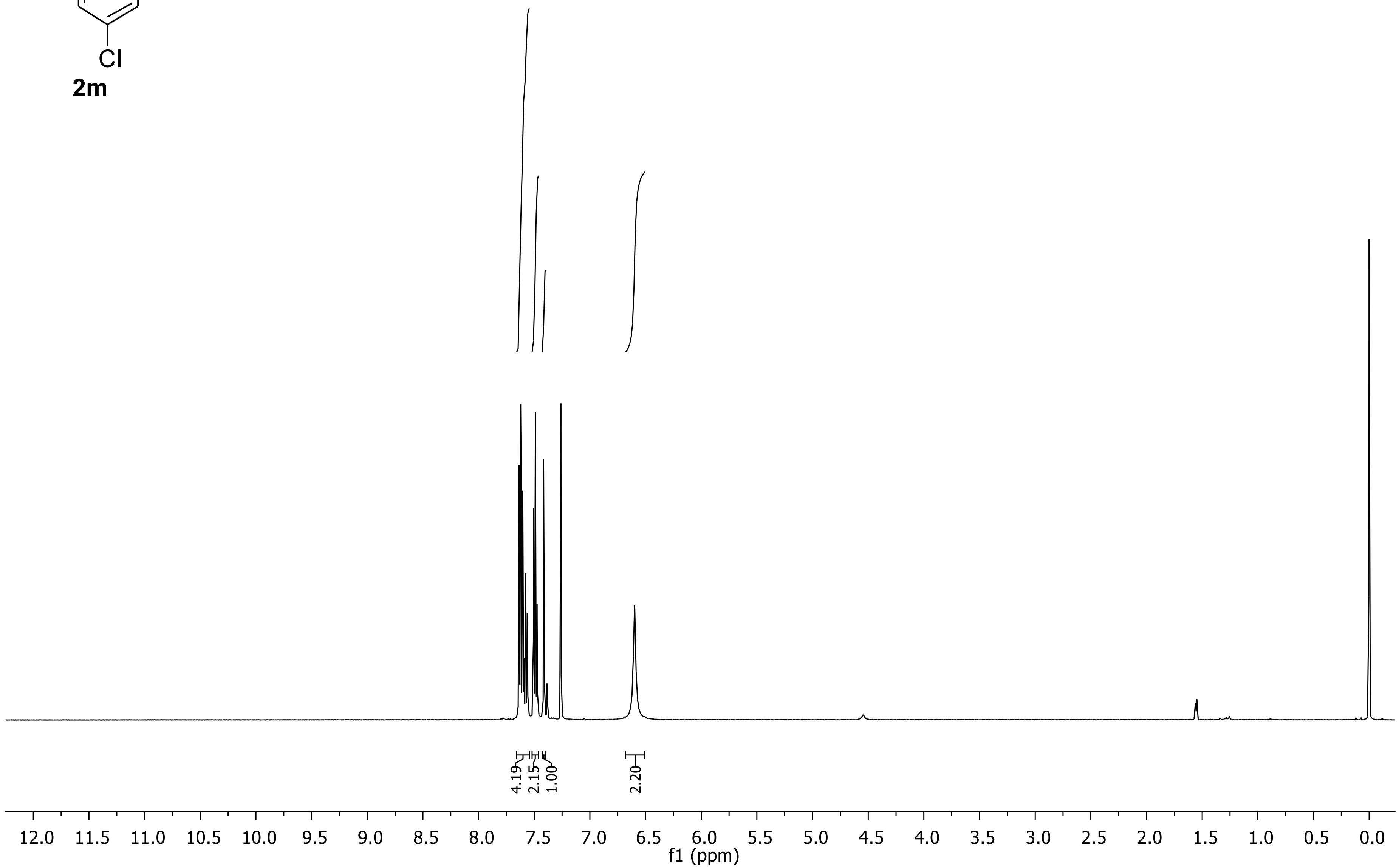
4.19
2.15
1.00
2.20
12.0 11.5 11.0 10.5 10.0 9.5 9.0 8.5 8.0 7.5 7.0 6.5 6.0 5.5 5.0 4.5 4.0 3.5 3.0 2.5 2.0 1.5 1.0 0.5 0.0
f1 (ppm)

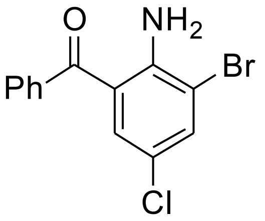

2m

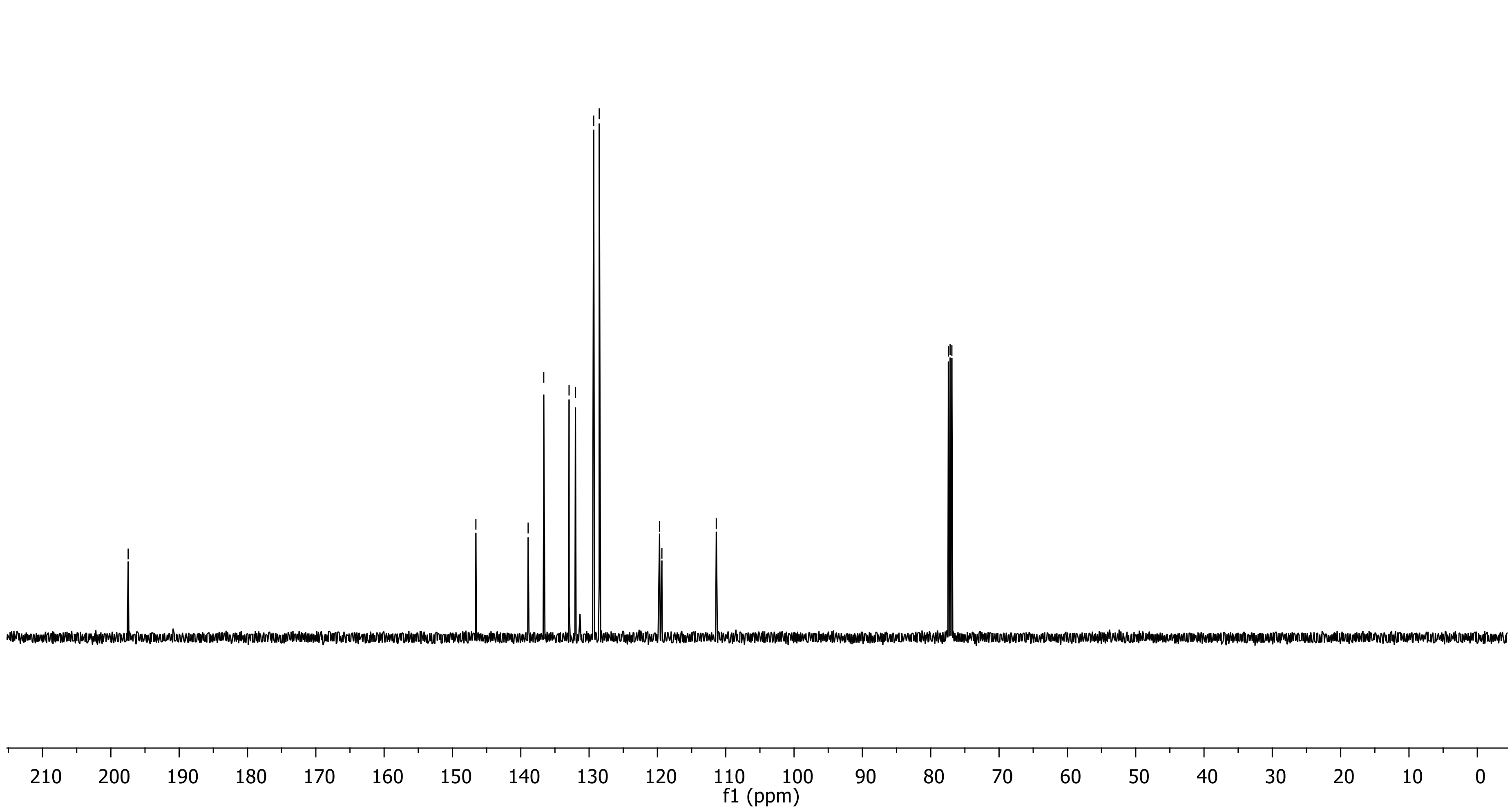

197.46
146.57
138.92
136.64
132.93
132.00
129.33
128.52
119.69
119.35
111.38
77.41
77.16
76.91
210 200 190 180 170 160 150 140 130 120 110 100 90 80 70 60 50 40 30 20 10 0
f1 (ppm)

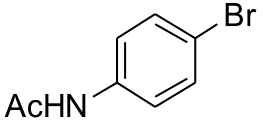
Br
AcHN

2n

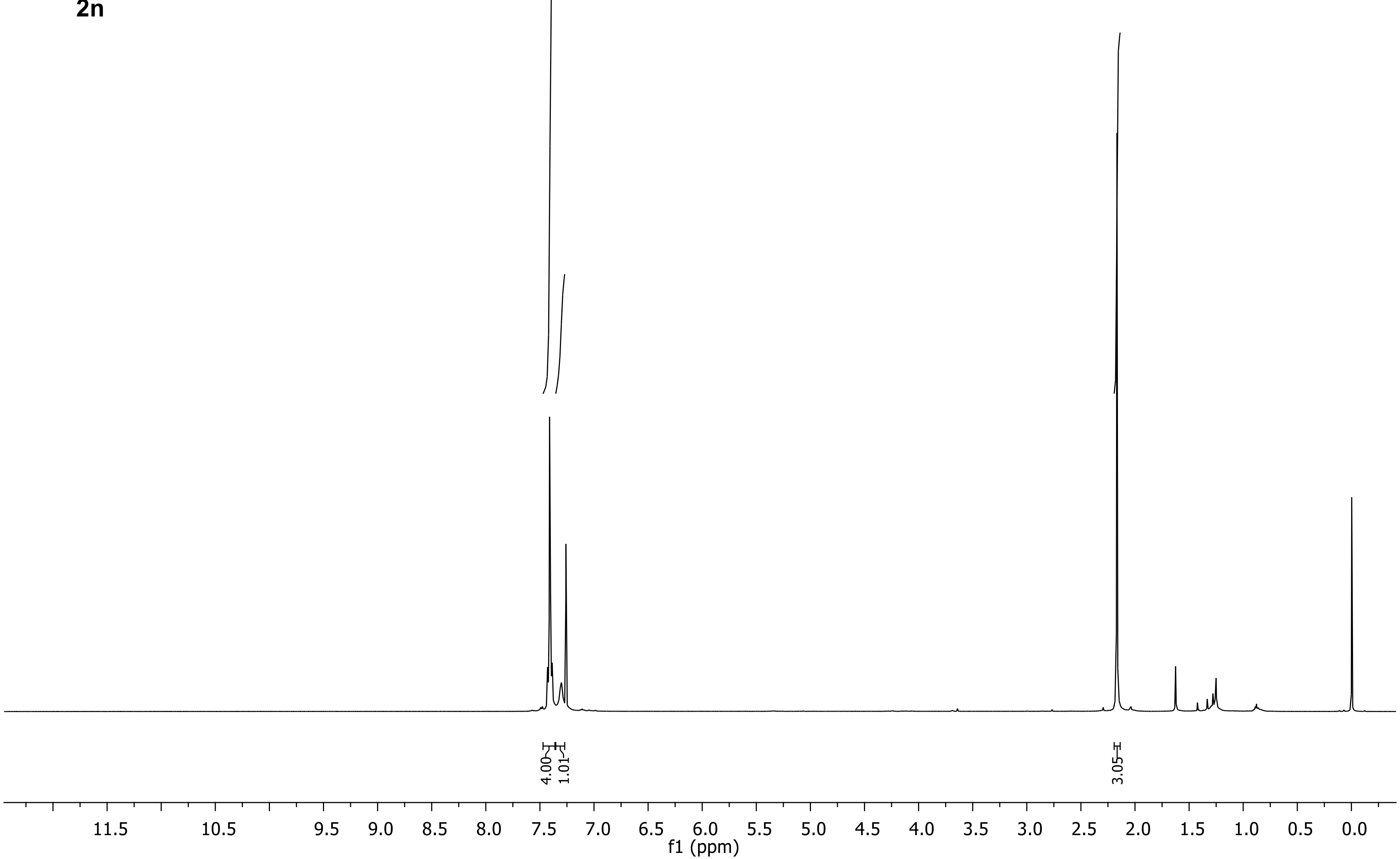
4.00
1.01
3.05
11.5
10.5
9.5
9.0
8.5
8.0
7.5
7.0
6.5
6.0
5.5
5.0
4.5
4.0
3.5
3.0
2.5
2.0
1.5
1.0
0.5
0.0
f1 (ppm)

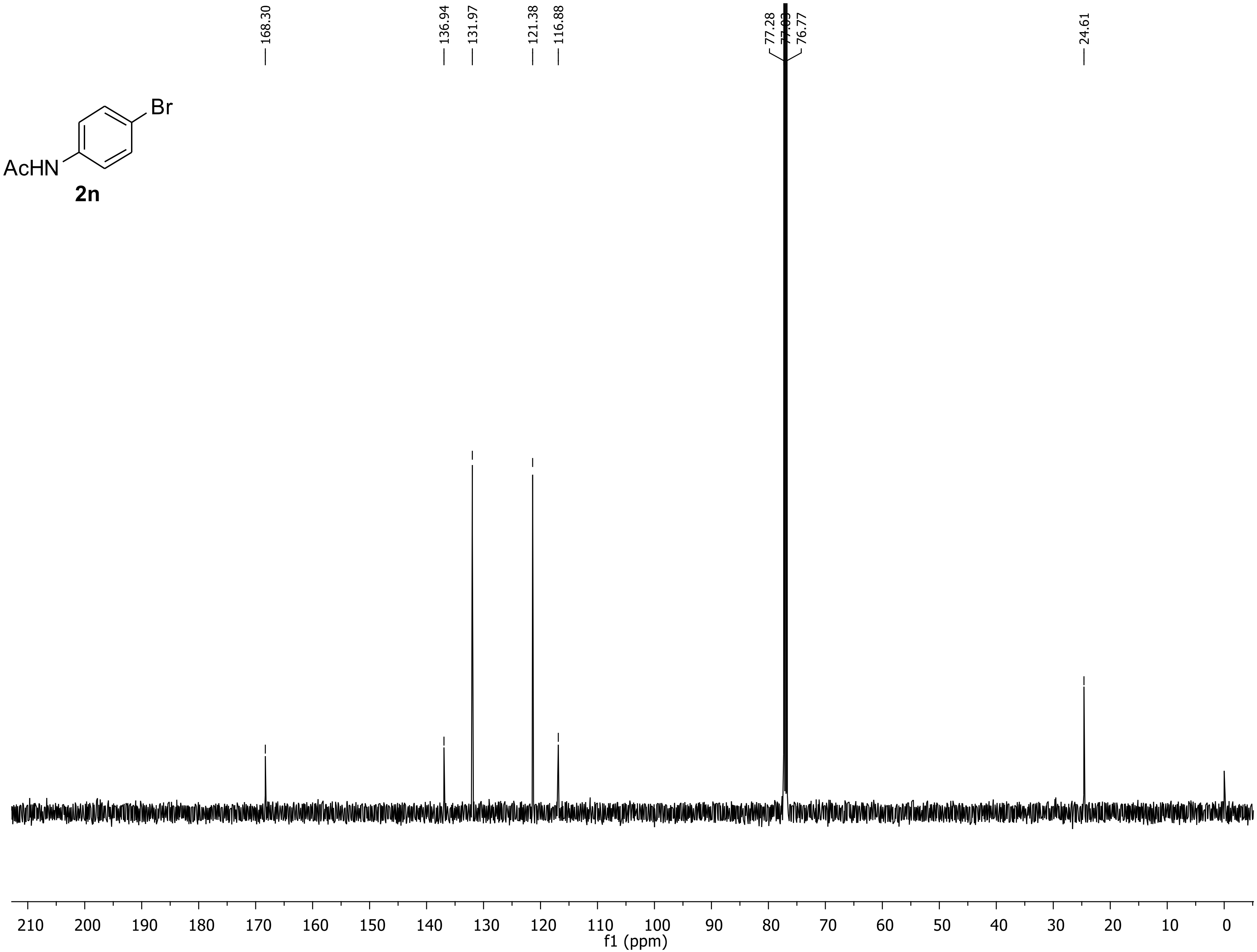
Br
AcHN
2n
168.30
136.94
131.97
121.38
116.88
77.28
77.03
76.77
24.61
210 200 190 180 170 160 150 140 130 120 110 100 90 80 70 60 50 40 30 20 10 0
f1 (ppm)

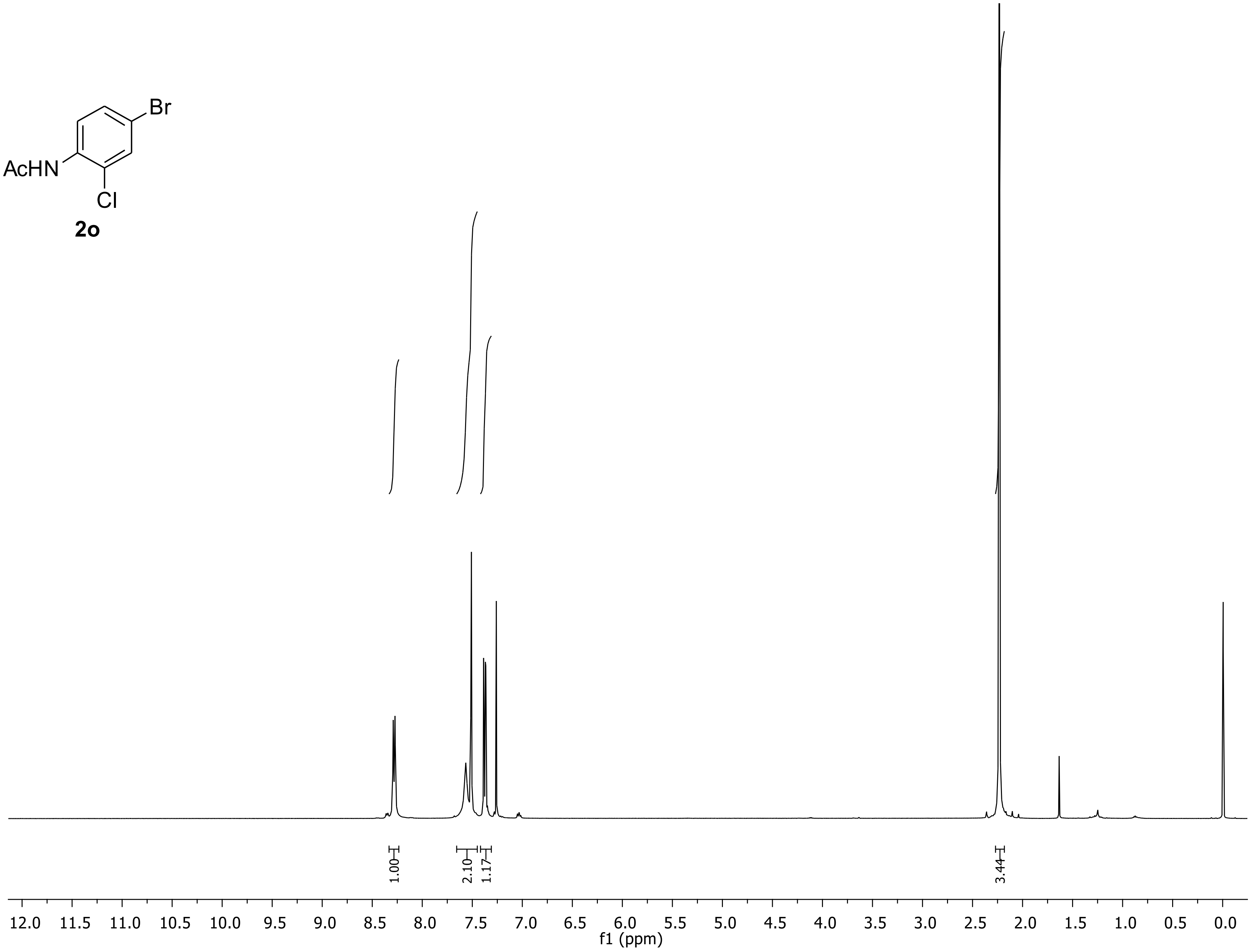
Br
AcHN
Cl
2o
1.00
2.10
1.17
3.44
12.0 11.5 11.0 10.5 10.0 9.5 9.0 8.5 8.0 7.5 7.0 6.5 6.0 5.5 5.0 4.5 4.0 3.5 3.0 2.5 2.0 1.5 1.0 0.5 0.0
f1 (ppm)

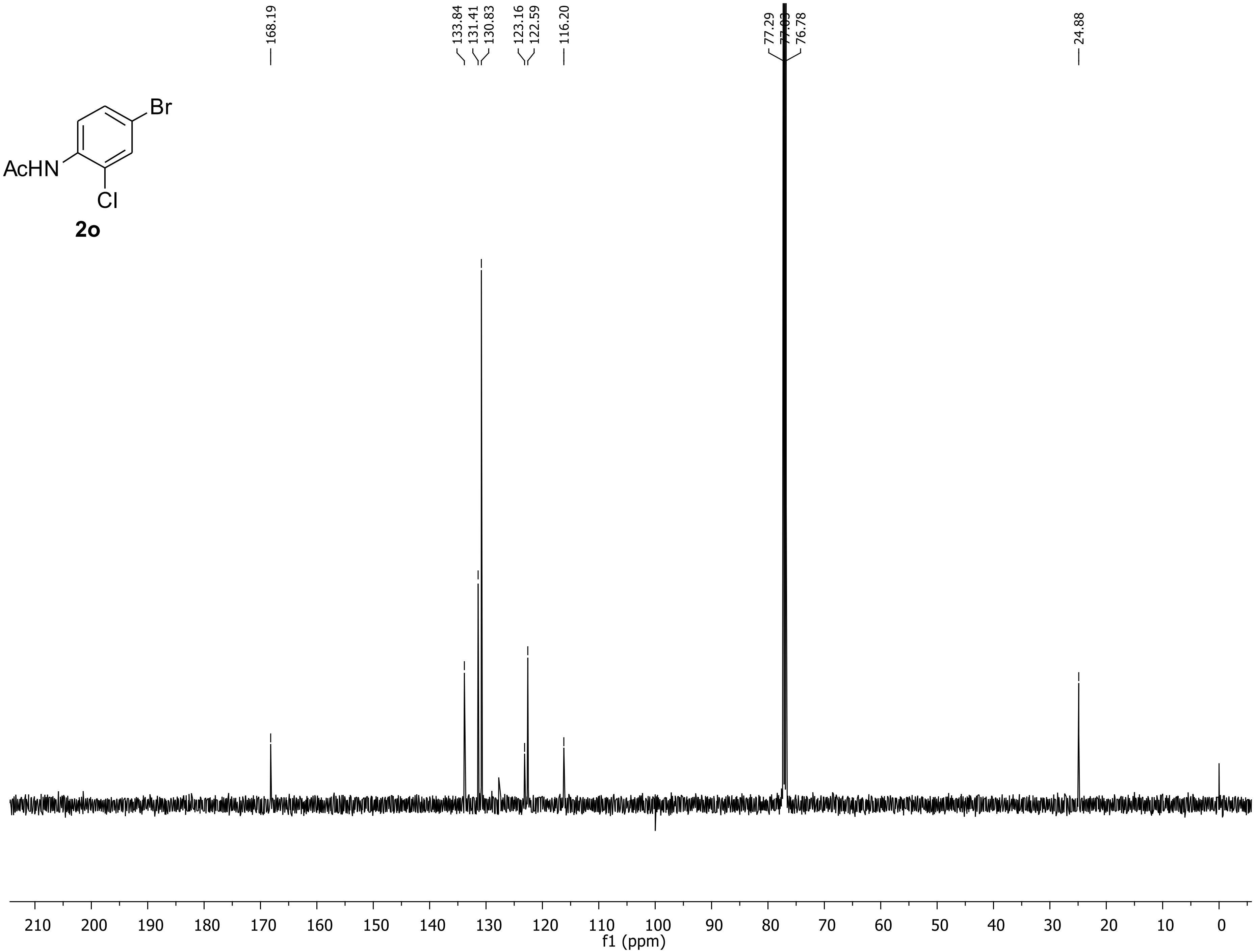
168.19
133.84
131.41
130.83
123.16
122.59
116.20
77.29
77.03
76.78
24.88
Br
AcHN
Cl
2o
210 200 190 180 170 160 150 140 130 120 110 100 90 80 70 60 50 40 30 20 10 0
f1 (ppm)

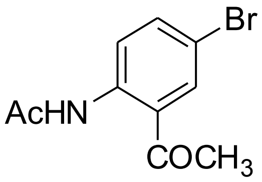
Br
AcHN
COCH3

2p

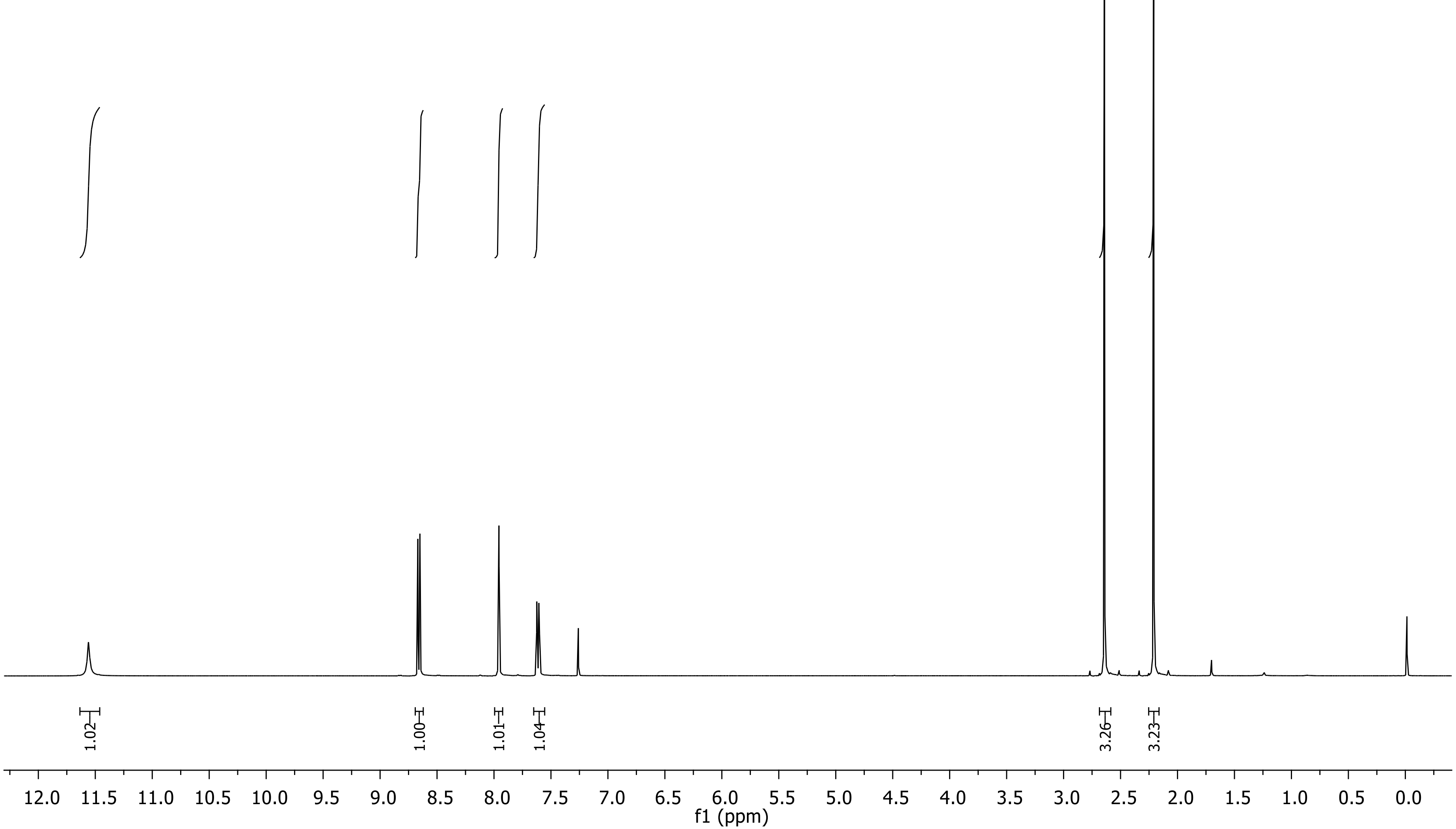
1.02
1.00
1.01
1.04
3.26
3.23
12.0 11.5 11.0 10.5 10.0 9.5 9.0 8.5 8.0 7.5 7.0 6.5 6.0 5.5 5.0 4.5 4.0 3.5 3.0 2.5 2.0 1.5 1.0 0.5 0.0
f1 (ppm)

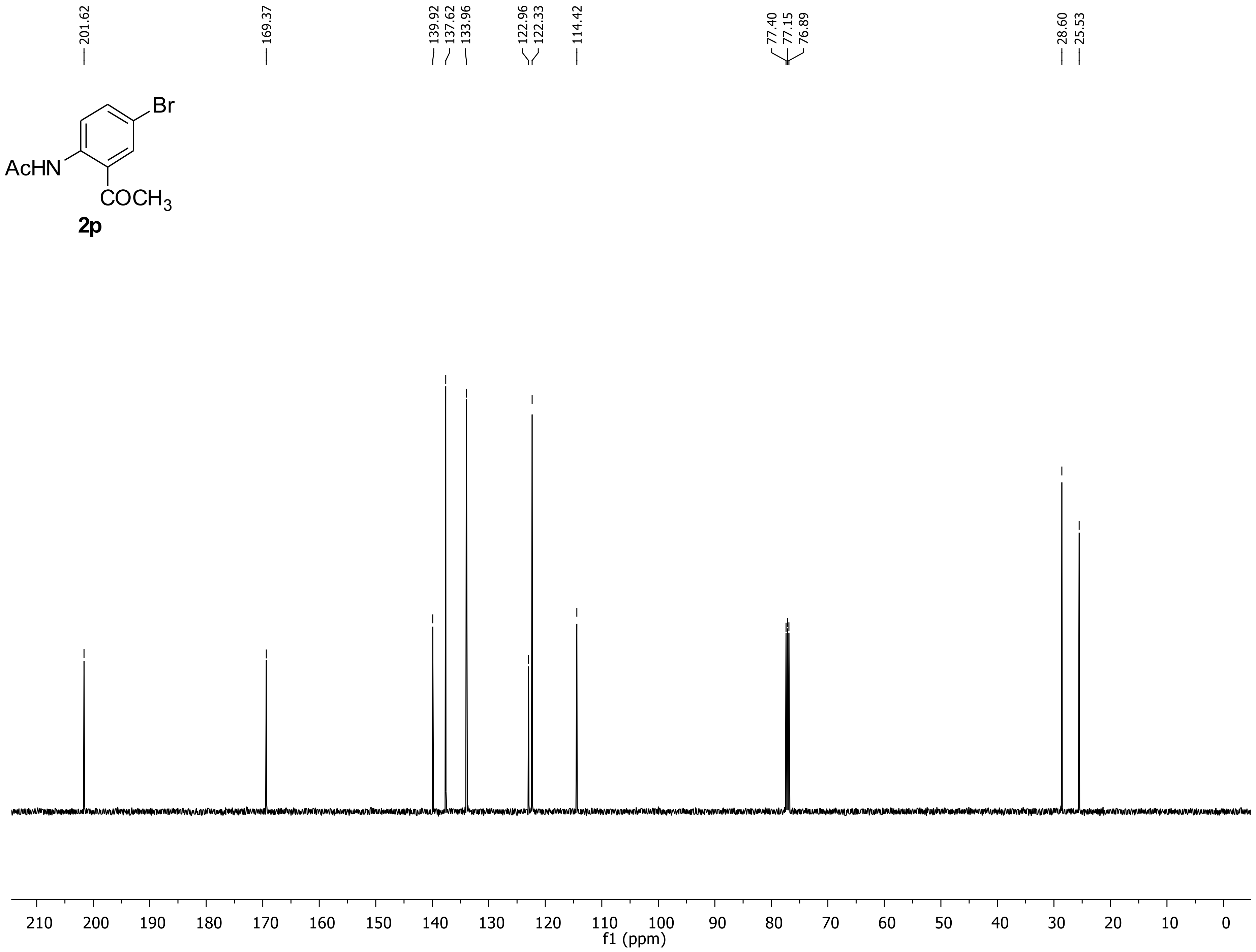

201.62
169.37
139.92
137.62
133.96
122.96
122.33
114.42
77.40
77.15
76.89
28.60
25.53
Br
AcHN
COCH3
2p
210
200
190
180
170
160
150
140
130
120
110
100
90
80
70
60
50
40
30
20
10
0
f1 (ppm)

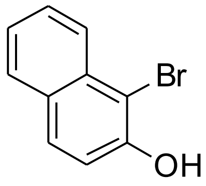
Br
OH

2q

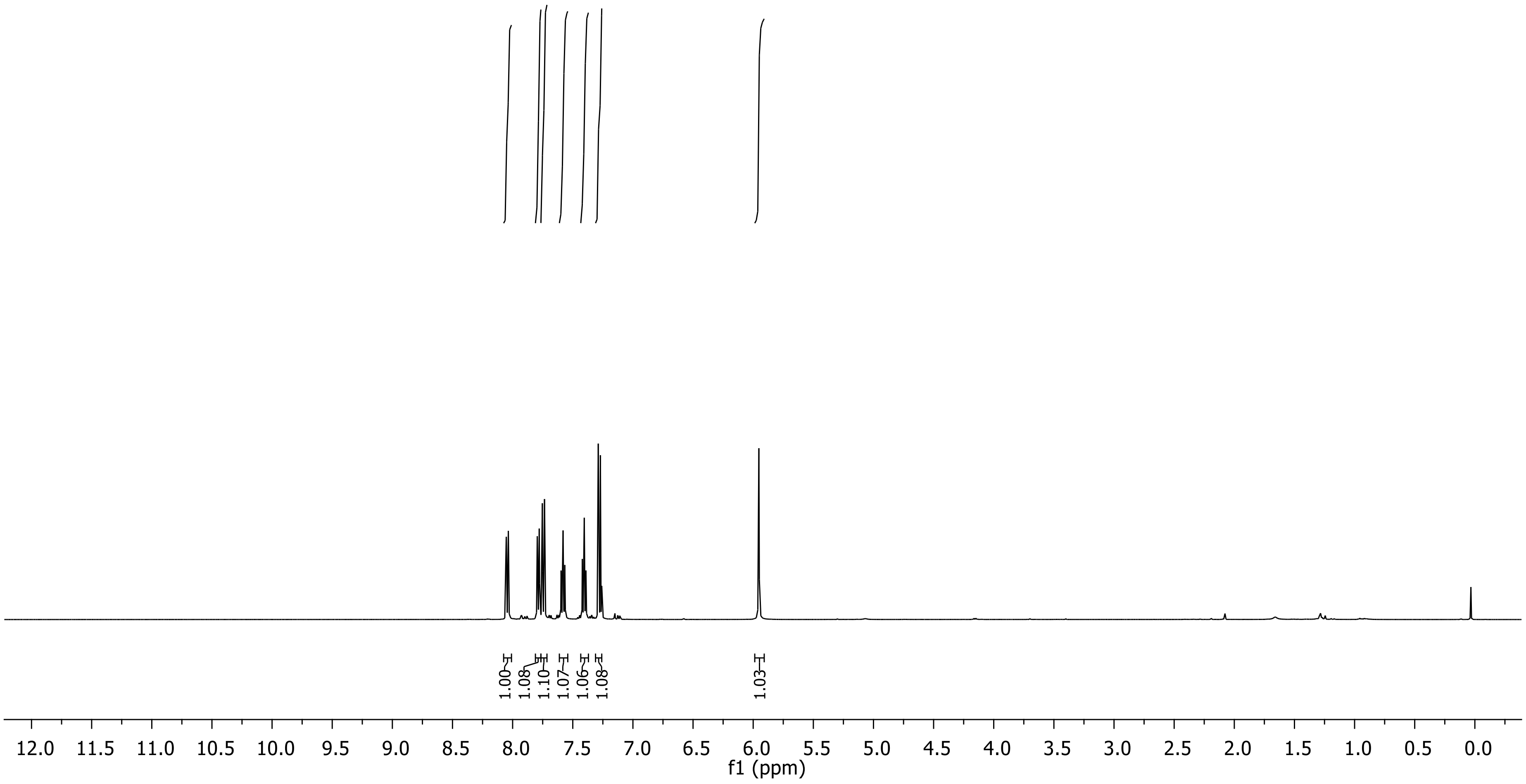
1.00
1.08
1.10
1.07
1.06
1.08
1.03
12.0 11.5 11.0 10.5 10.0 9.5 9.0 8.5 8.0 7.5 7.0 6.5 6.0 5.5 5.0 4.5 4.0 3.5 3.0 2.5 2.0 1.5 1.0 0.5 0.0
f1 (ppm)

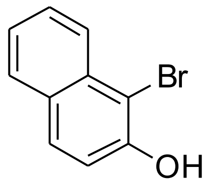
Br
OH

2q

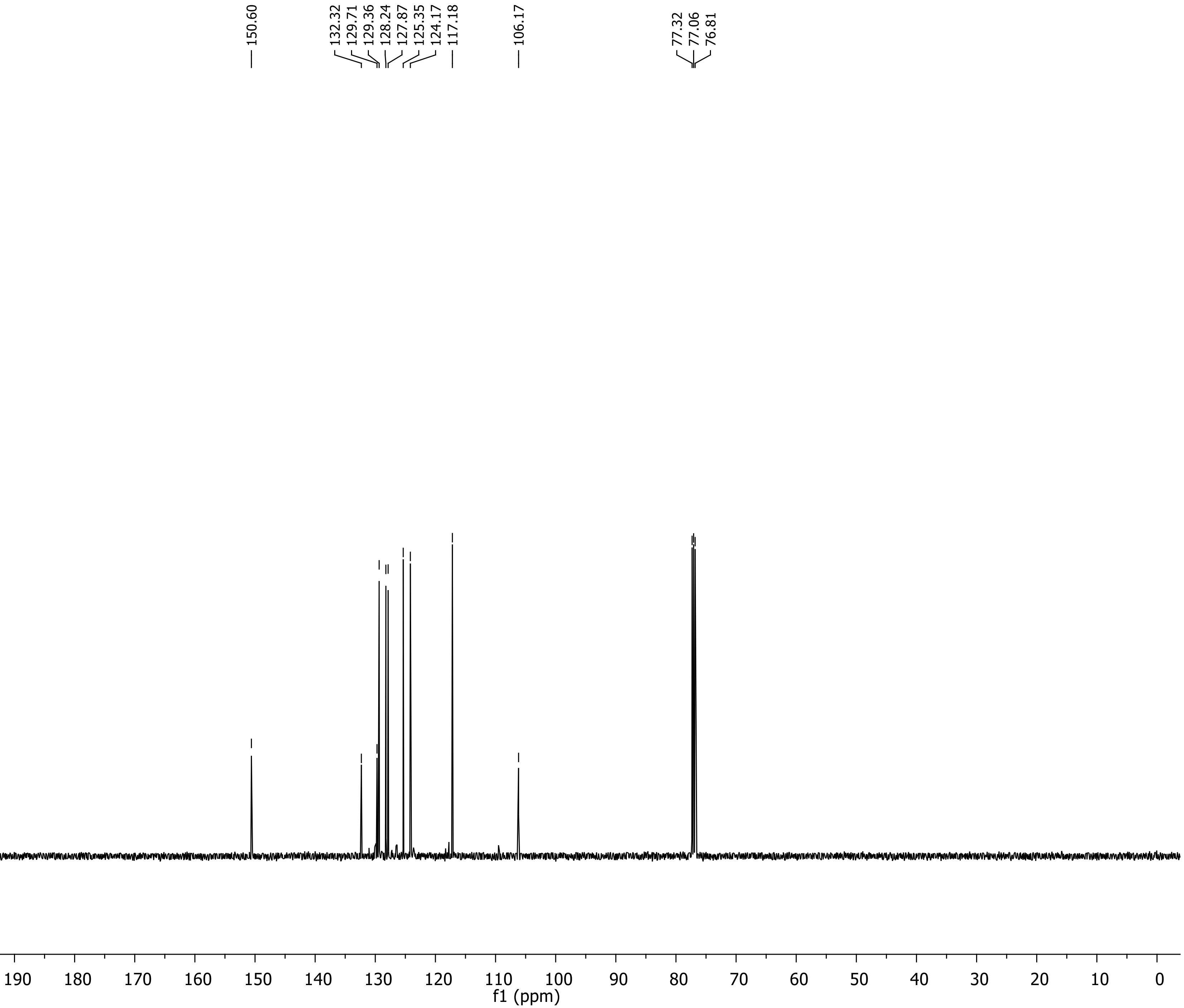
150.60
132.32
129.71
129.36
128.24
127.87
125.35
124.17
117.18
106.17
77.32
77.06
76.81
210 200 190 180 170 160 150 140 130 120 110 100 90 80 70 60 50 40 30 20 10 0
f1 (ppm)

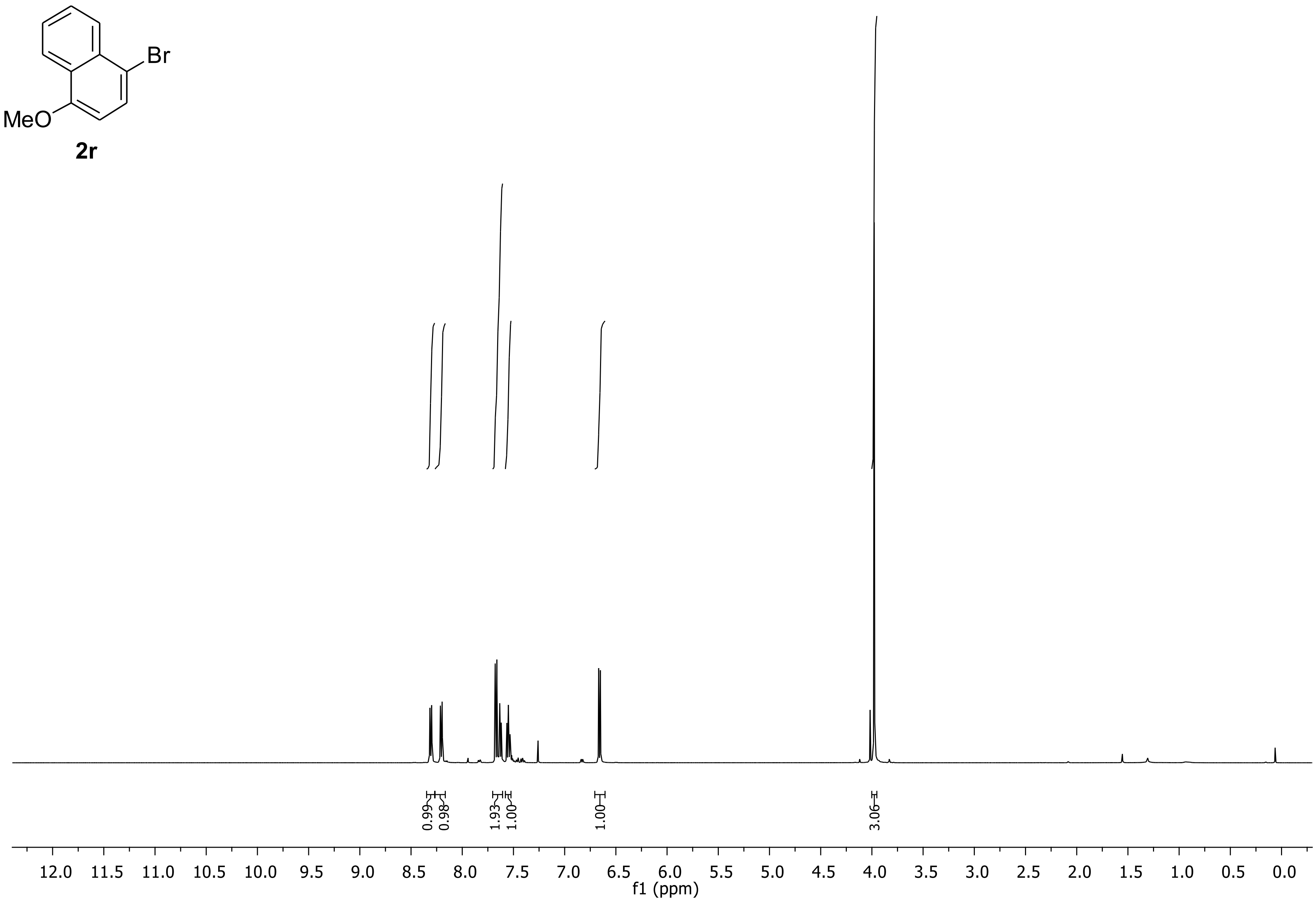
Br
MeO
0.99
0.98
1.93
1.00
1.00
3.06
12.0 11.5 11.0 10.5 10.0 9.5 9.0 8.5 8.0 7.5 7.0 6.5 6.0 5.5 5.0 4.5 4.0 3.5 3.0 2.5 2.0 1.5 1.0 0.5 0.0
f1 (ppm)

2r

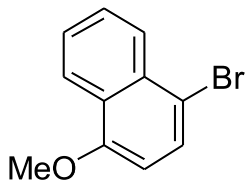

2r

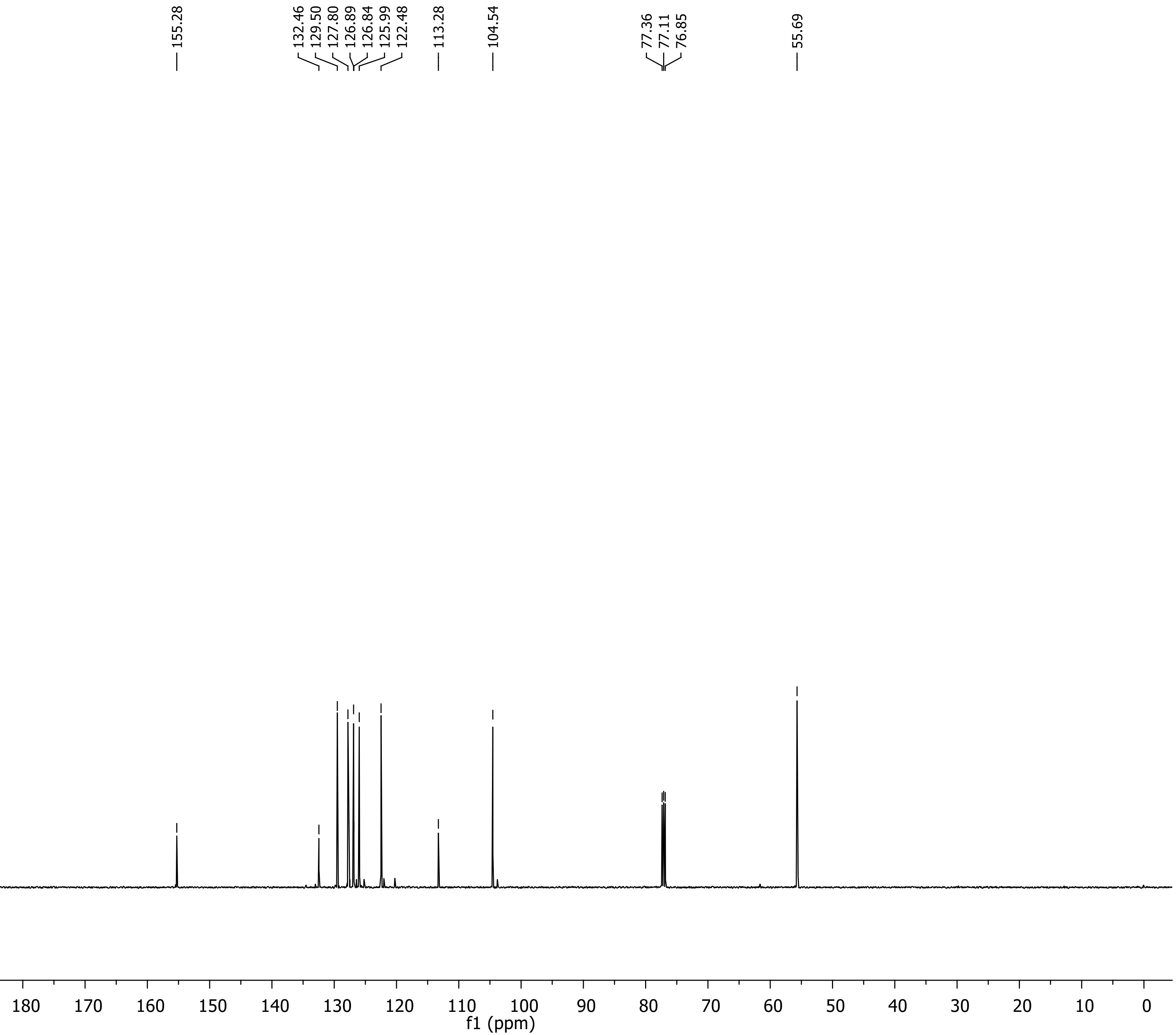
155.28
132.46
129.50
127.80
126.89
126.84
125.99
122.48
113.28
104.54
77.36
77.11
76.85
55.69
210
200
190
180
170
160
150
140
130
120
110
100
90
80
70
60
50
40
30
20
10
0
f1 (ppm)

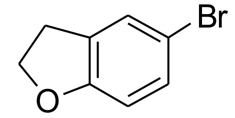

2s

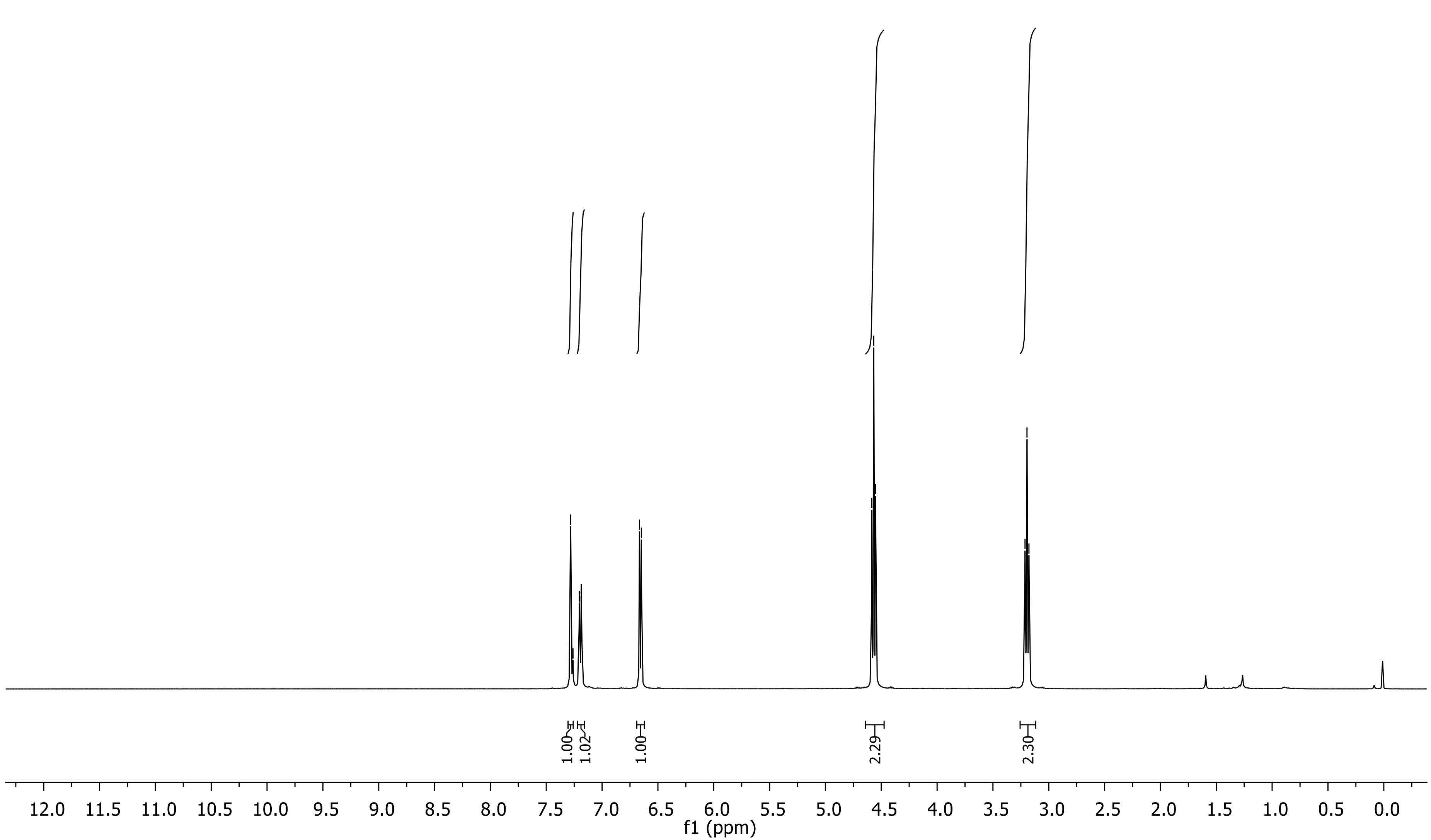

7.28
7.26
7.20
7.20
7.19
7.18
6.66
6.65
4.58
4.57
4.55
3.21
3.20
3.18
1.00
1.02
1.00
2.29
2.30
12.0 11.5 11.0 10.5 10.0 9.5 9.0 8.5 8.0 7.5 7.0 6.5 6.0 5.5 5.0 4.5 4.0 3.5 3.0 2.5 2.0 1.5 1.0 0.5 0.0
f1 (ppm)

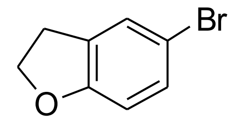
Br
O

2s

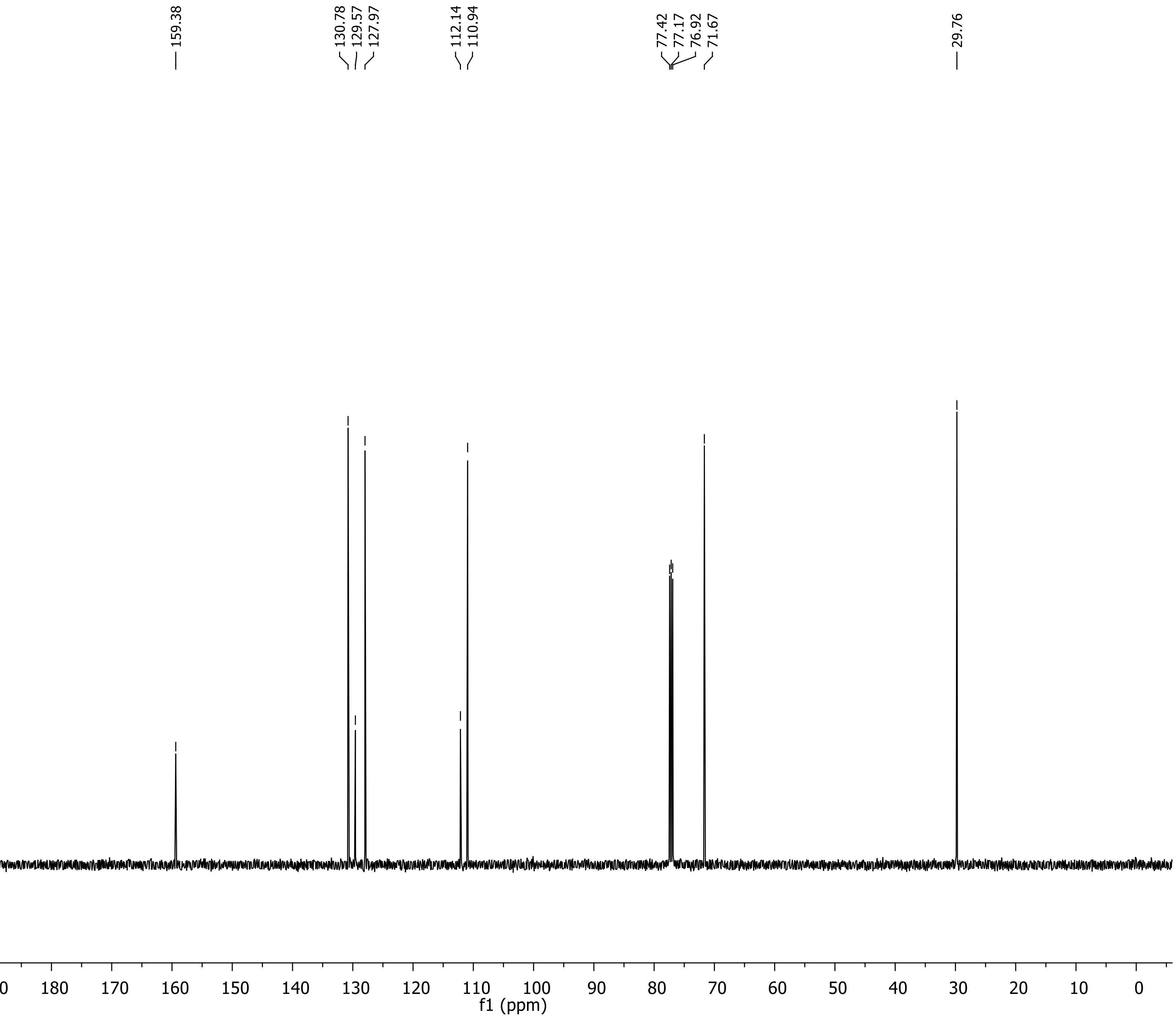
159.38
130.78
129.57
127.97
112.14
110.94
77.42
77.17
76.92
71.67
29.76
210 200 190 180 170 160 150 140 130 120 110 100 90 80 70 60 50 40 30 20 10 0
f1 (ppm)

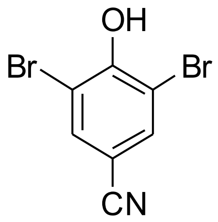
OH
Br
Br
CN

2t

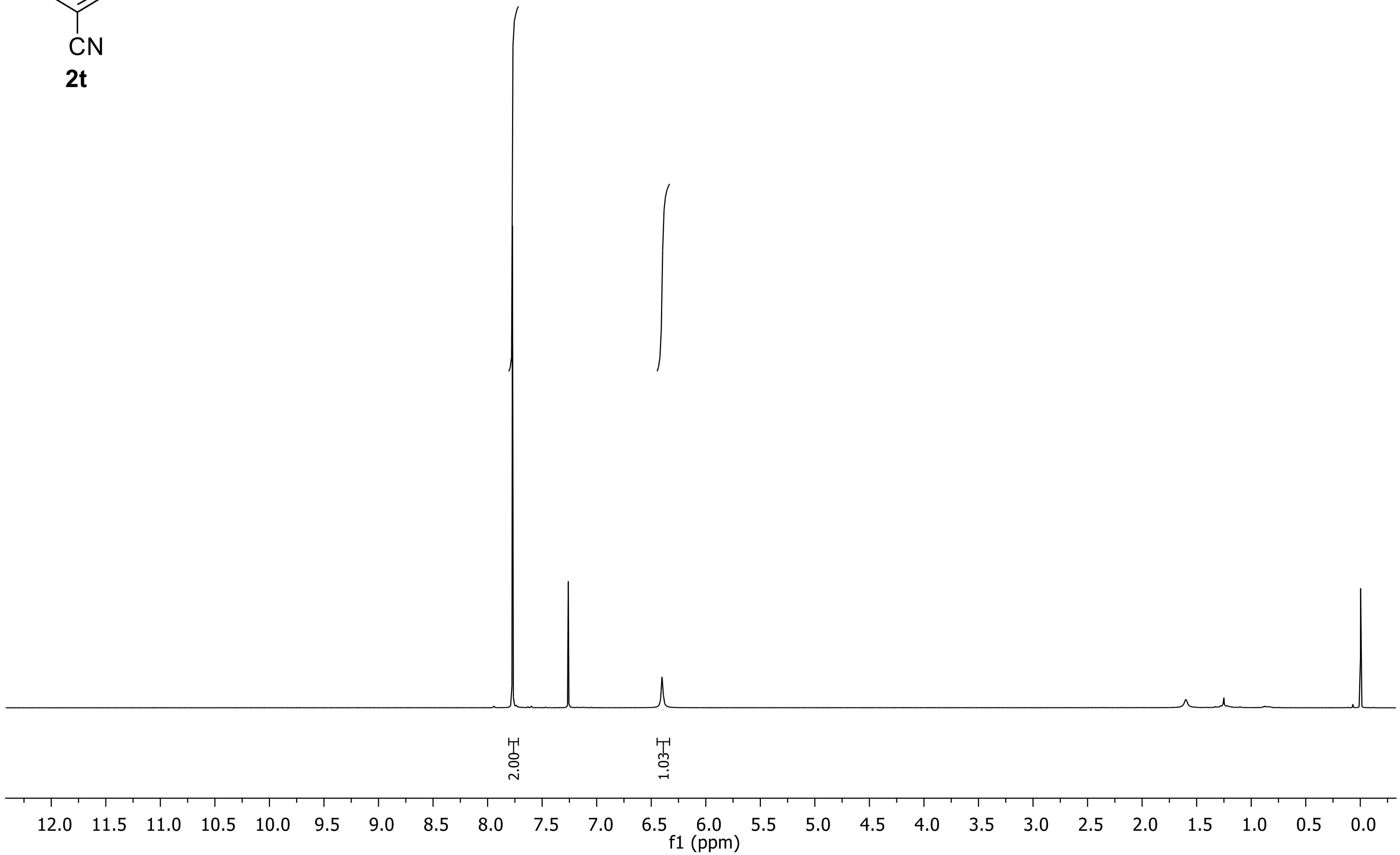
2.00
1.03
12.0 11.5 11.0 10.5 10.0 9.5 9.0 8.5 8.0 7.5 7.0 6.5 6.0 5.5 5.0 4.5 4.0 3.5 3.0 2.5 2.0 1.5 1.0 0.5 0.0
f1 (ppm)

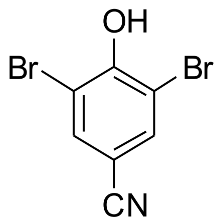
OH
Br
Br
CN

2t

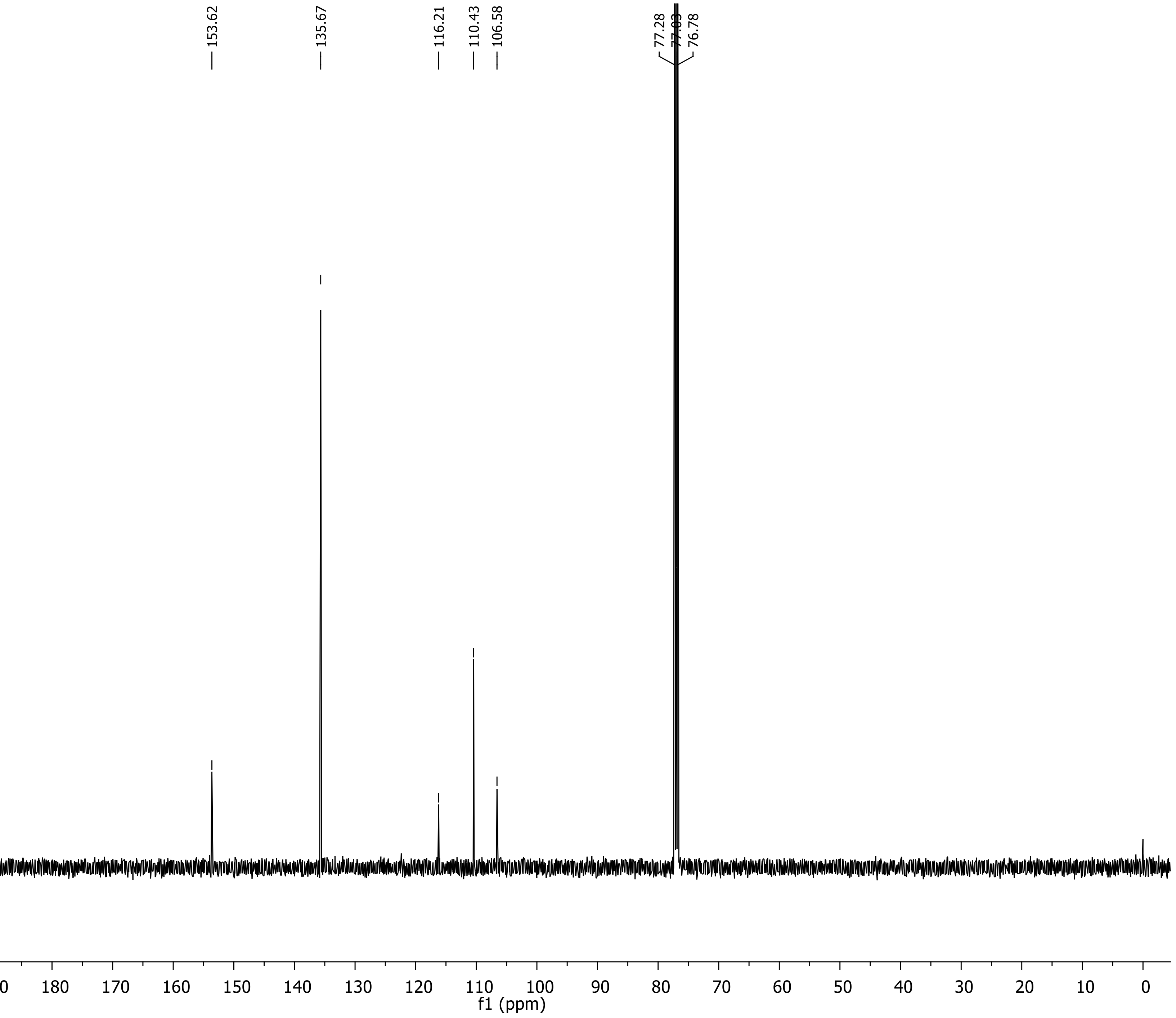
153.62
135.67
116.21
110.43
106.58
77.28
77.03
76.78
210
200
190
180
170
160
150
140
130
120
110
100
90
80
70
60
50
40
30
20
10
0
f1 (ppm)

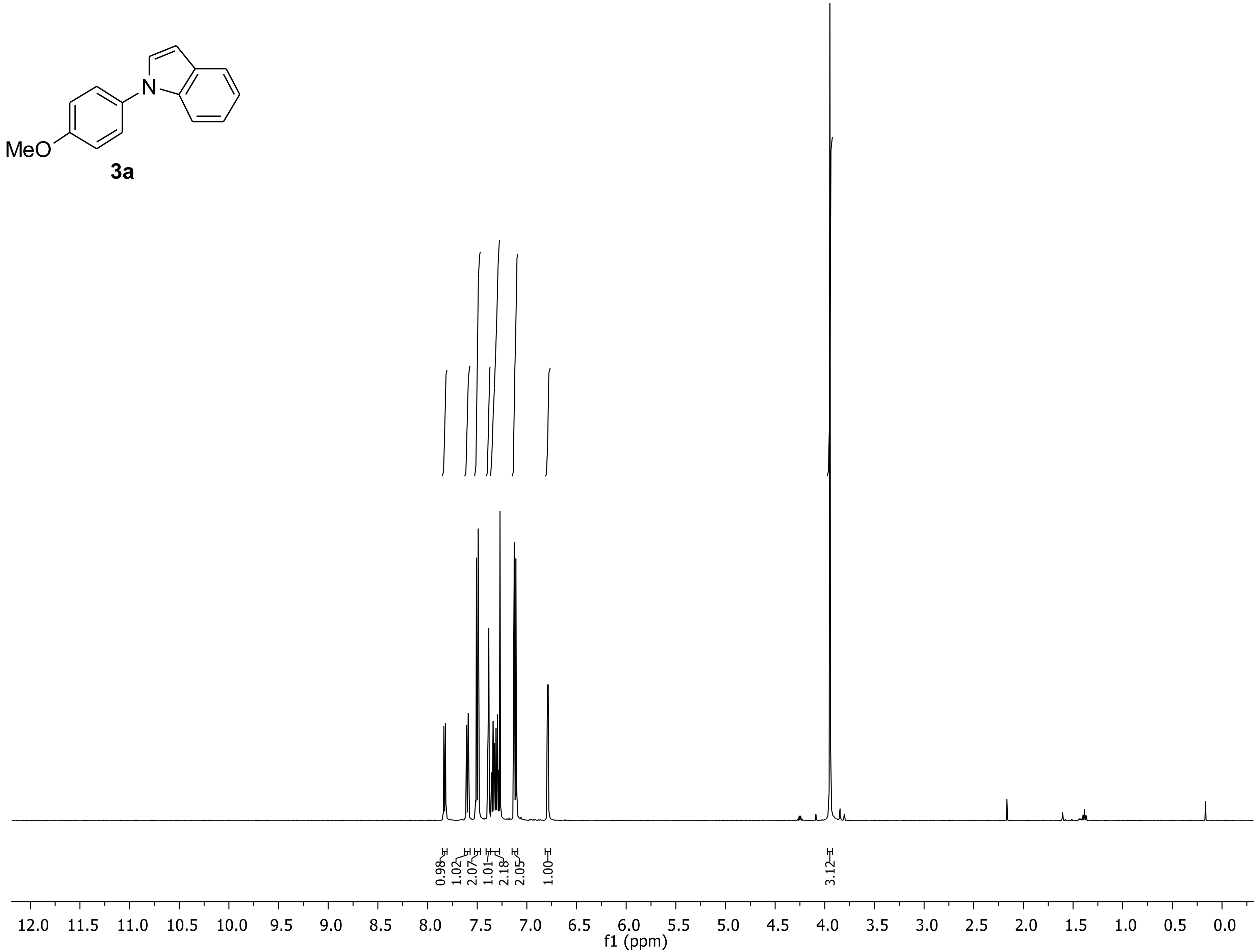
MeO
N
3a
0.98
1.02
2.07
1.01
2.18
2.05
1.00
3.12
12.0 11.5 11.0 10.5 10.0 9.5 9.0 8.5 8.0 7.5 7.0 6.5 6.0 5.5 5.0 4.5 4.0 3.5 3.0 2.5 2.0 1.5 1.0 0.5 0.0
f1 (ppm)

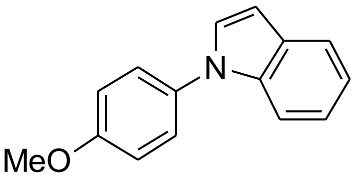

3a

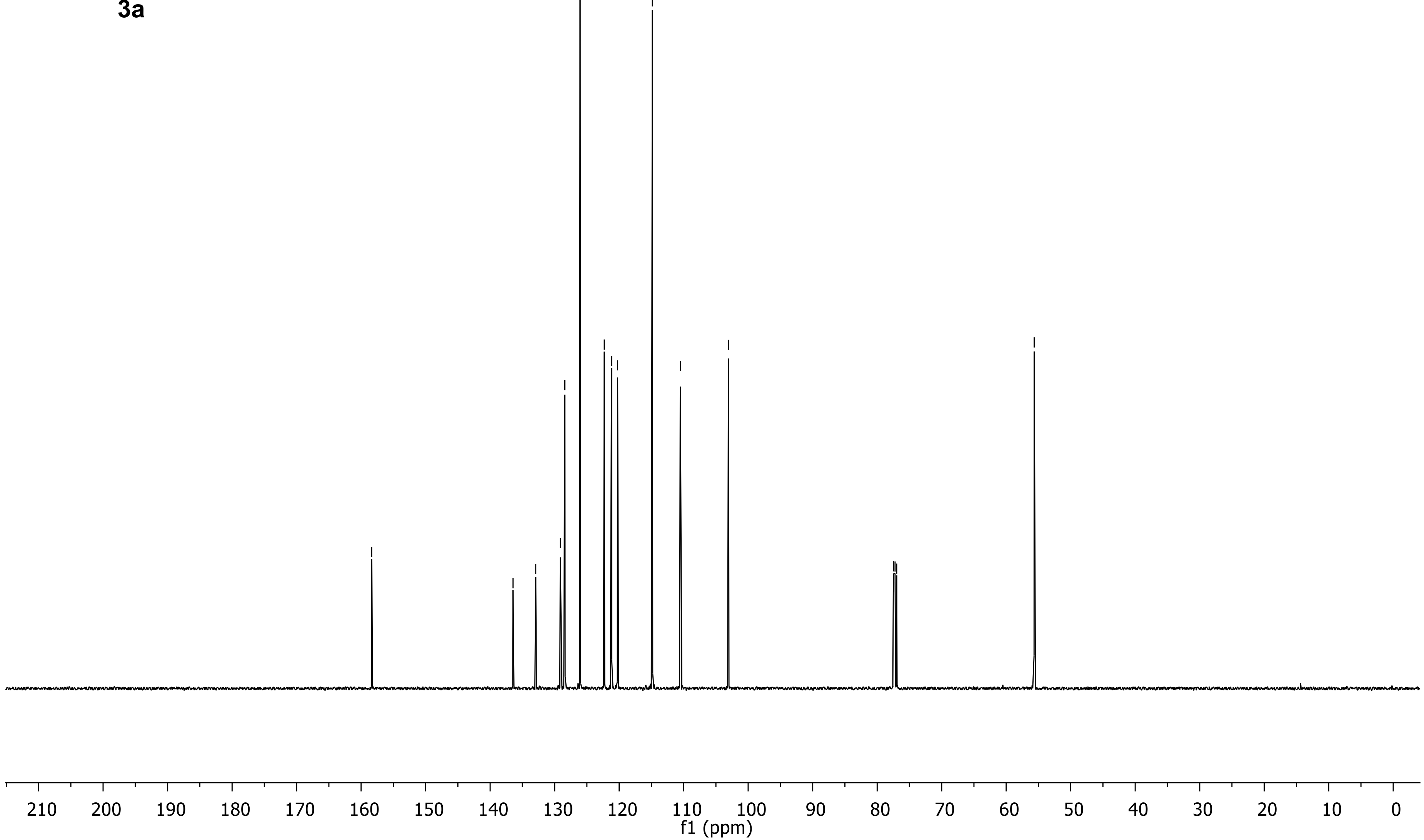

158.34
136.44
132.93
129.12
128.41
126.06
122.30
121.17
120.24
114.85
110.52
103.05
77.48
77.43
77.23
76.97
55.66
210 200 190 180 170 160 150 140 130 120 110 100 90 80 70 60 50 40 30 20 10 0
f1 (ppm)

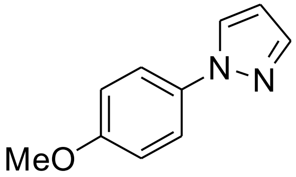
N
N
MeO

3b

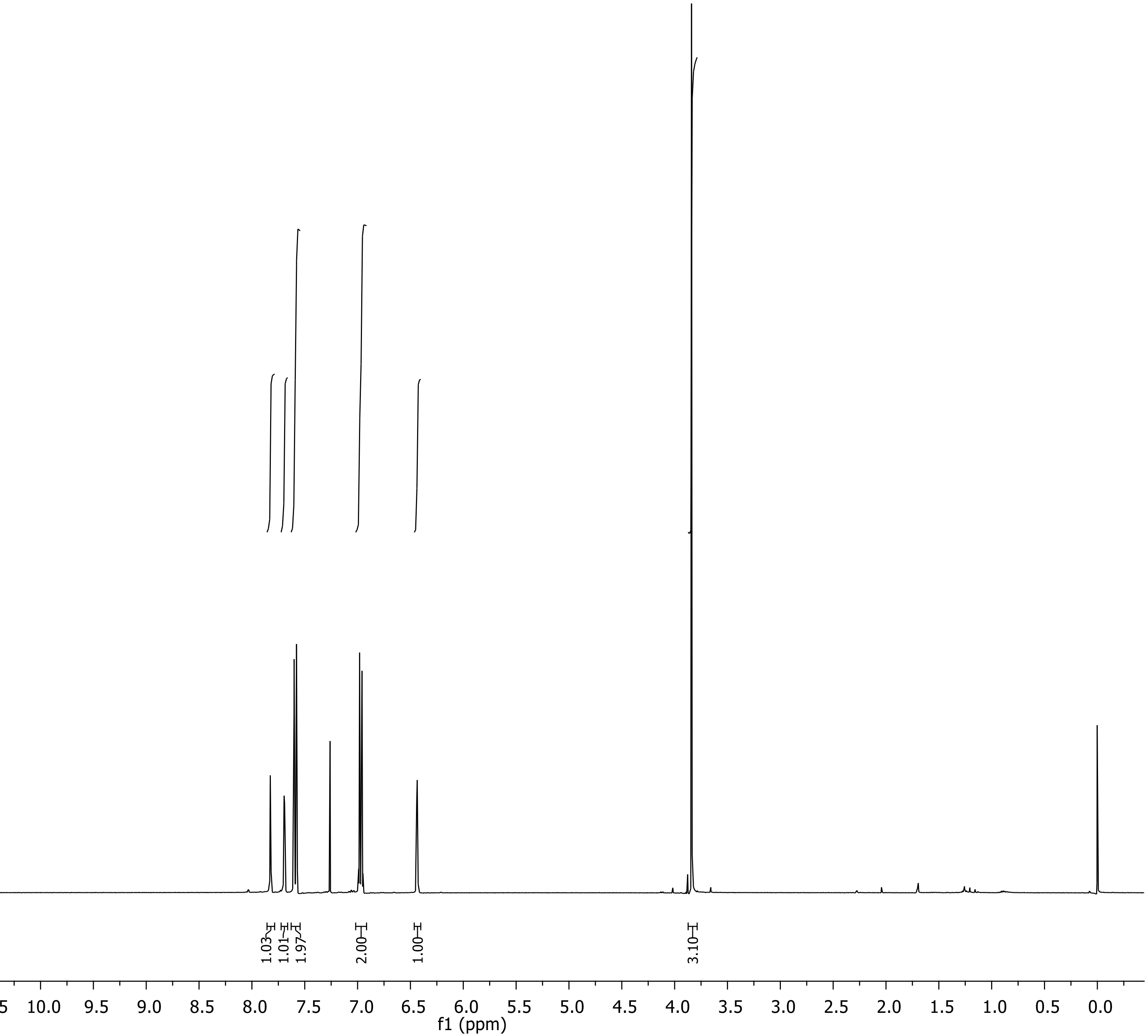
1.03
1.01
1.97
2.00
1.00
3.10
12.0 11.5 11.0 10.5 10.0 9.5 9.0 8.5 8.0 7.5 7.0 6.5 6.0 5.5 5.0 4.5 4.0 3.5 3.0 2.5 2.0 1.5 1.0 0.5 0.0
f1 (ppm)

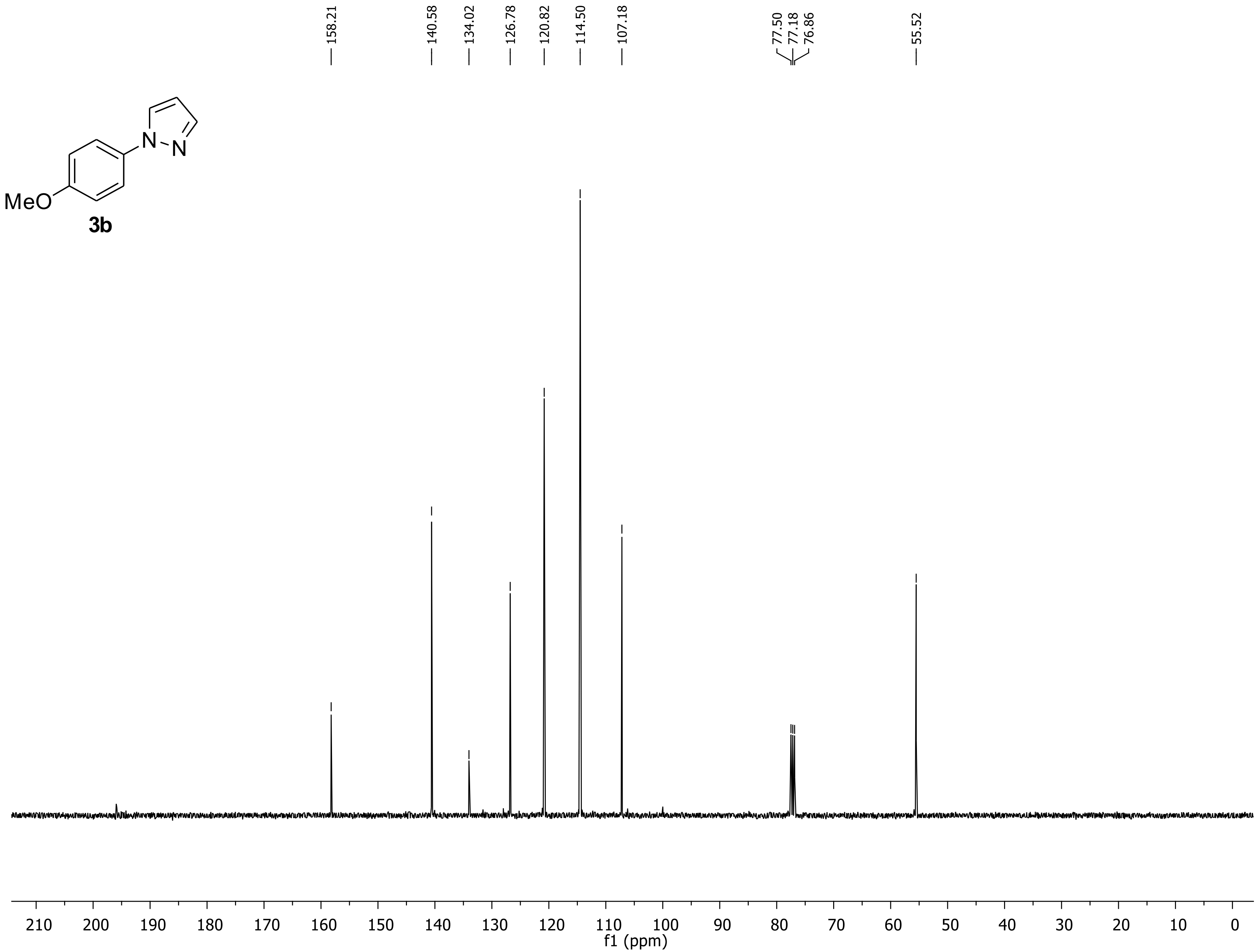
158.21
140.58
134.02
126.78
120.82
114.50
107.18
77.50
77.18
76.86
55.52
MeO
N
N
3b
210
200
190
180
170
160
150
140
130
120
110
100
90
80
70
60
50
40
30
20
10
0
f1 (ppm)

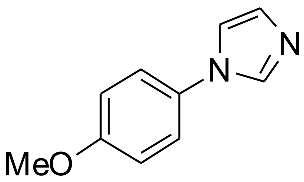

3c

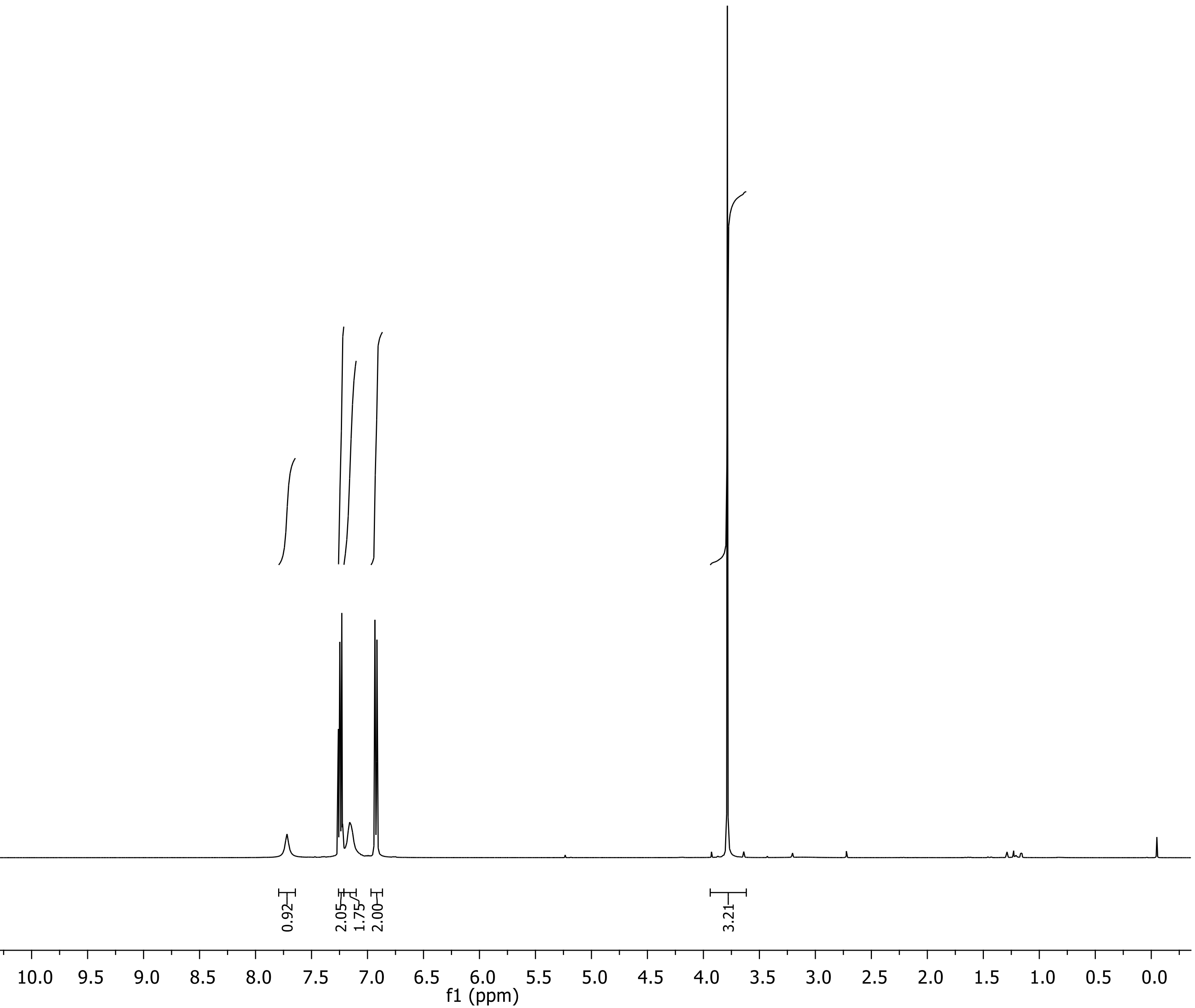

0.92
2.05
1.75
2.00
3.21
12.0 11.5 11.0 10.5 10.0 9.5 9.0 8.5 8.0 7.5 7.0 6.5 6.0 5.5 5.0 4.5 4.0 3.5 3.0 2.5 2.0 1.5 1.0 0.5 0.0
f1 (ppm)

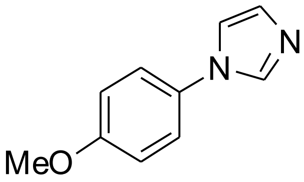
N
N
MeO

3c

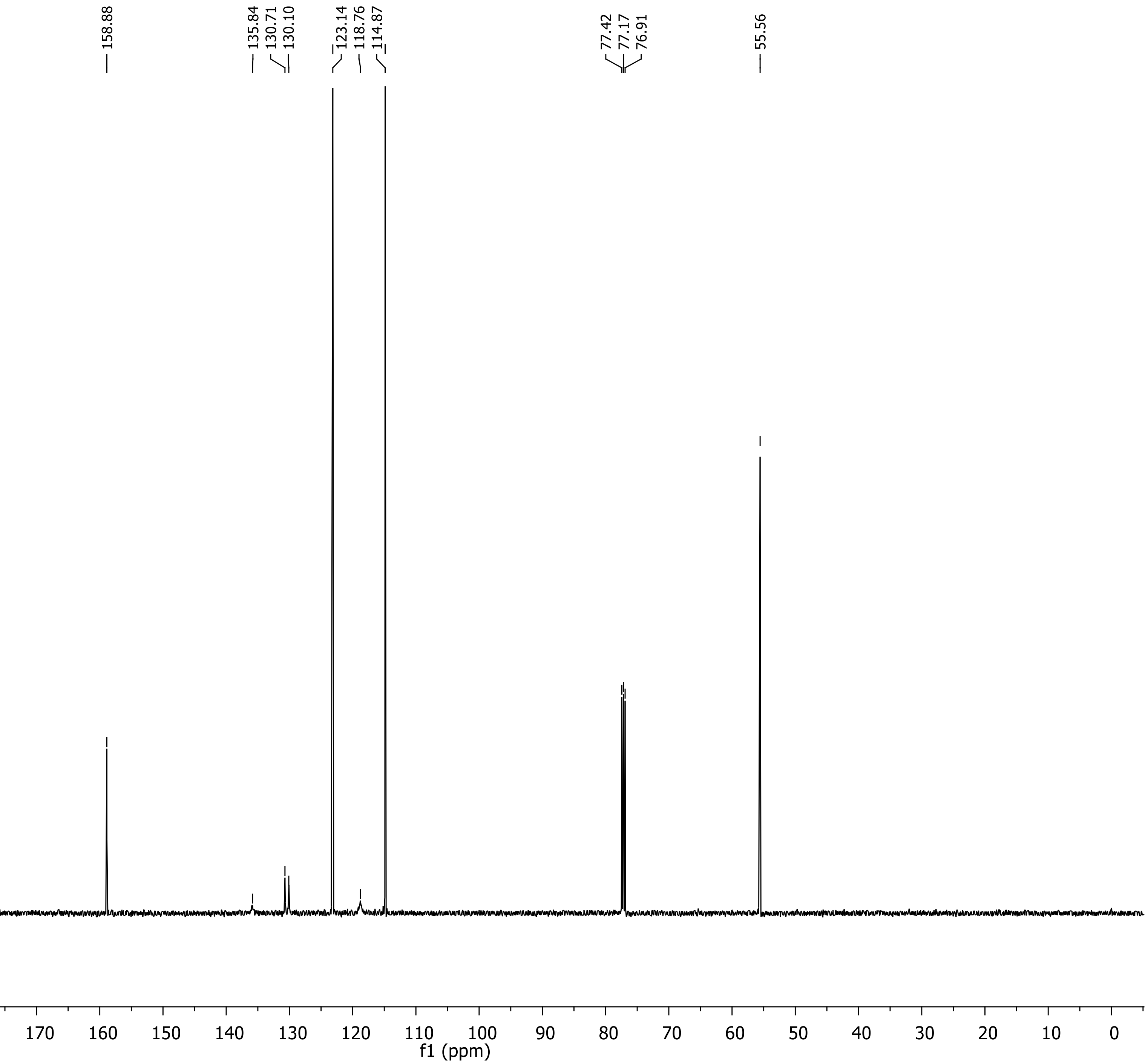
158.88
135.84
130.71
130.10
123.14
118.76
114.87
77.42
77.17
76.91
55.56
210 200 190 180 170 160 150 140 130 120 110 100 90 80 70 60 50 40 30 20 10 0
f1 (ppm)

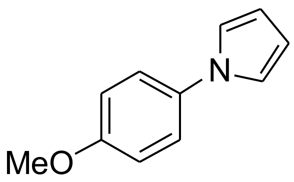
MeO
N

3d

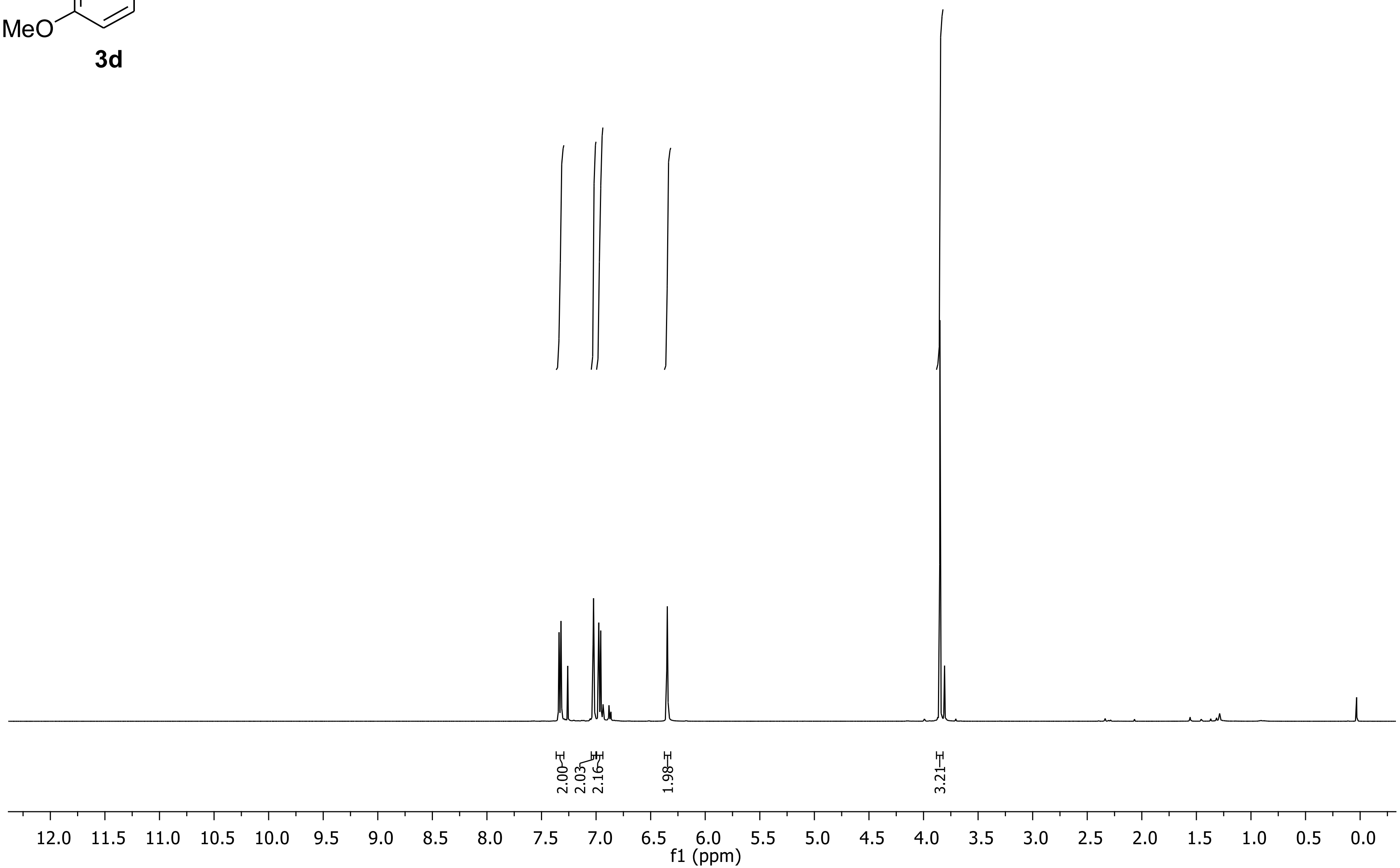
2.00
2.03
2.16
1.98
3.21
12.0 11.5 11.0 10.5 10.0 9.5 9.0 8.5 8.0 7.5 7.0 6.5 6.0 5.5 5.0 4.5 4.0 3.5 3.0 2.5 2.0 1.5 1.0 0.5 0.0
f1 (ppm)

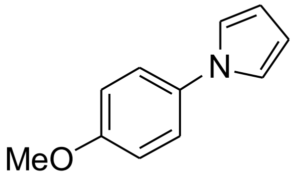
N
MeO

3d

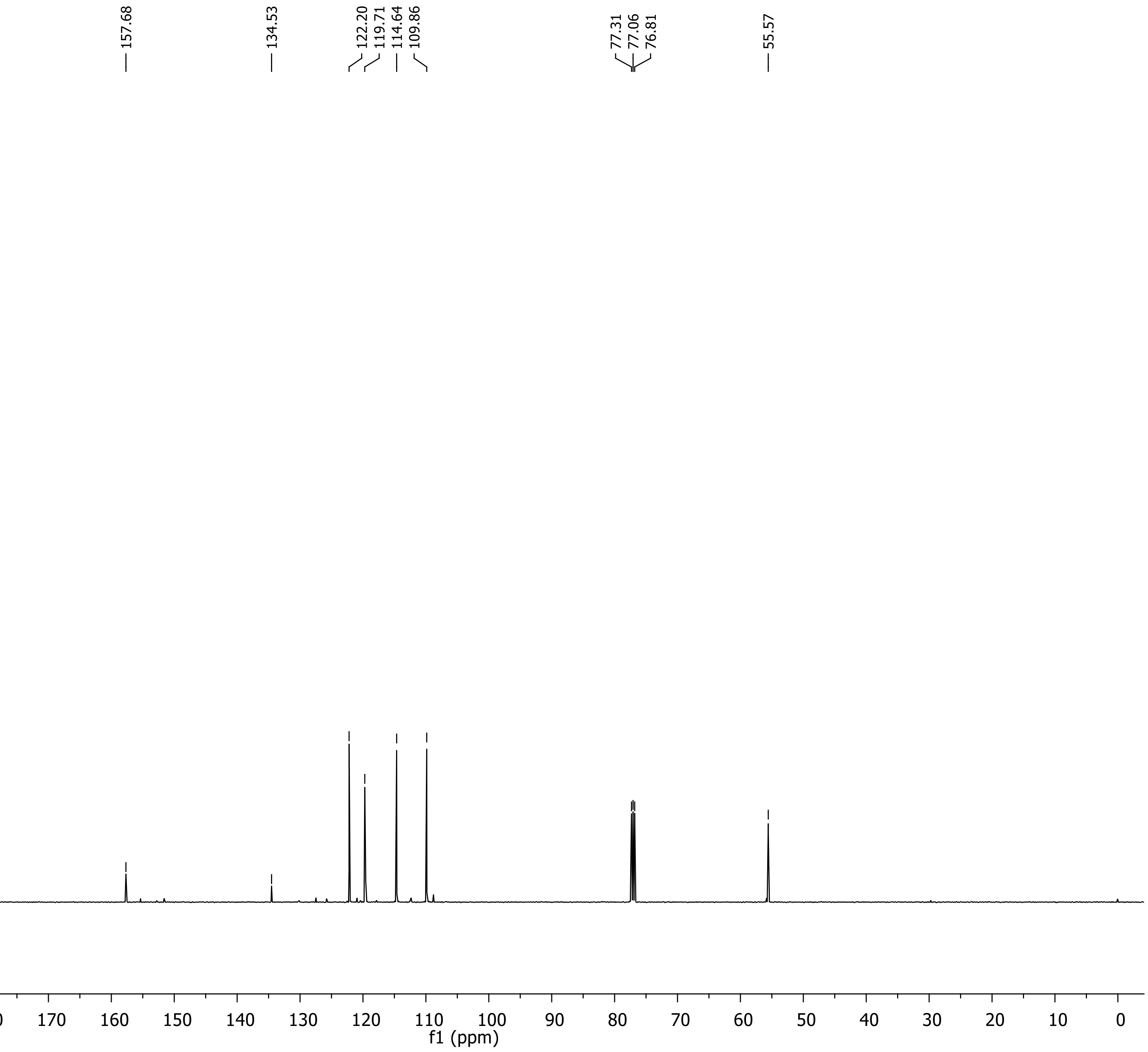
157.68
134.53
122.20
119.71
114.64
109.86
77.31
77.06
76.81
55.57
210 200 190 180 170 160 150 140 130 120 110 100 90 80 70 60 50 40 30 20 10 0
f1 (ppm)

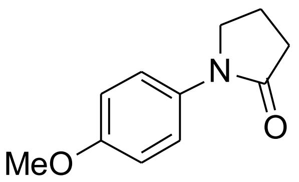
MeO
N
O

3e

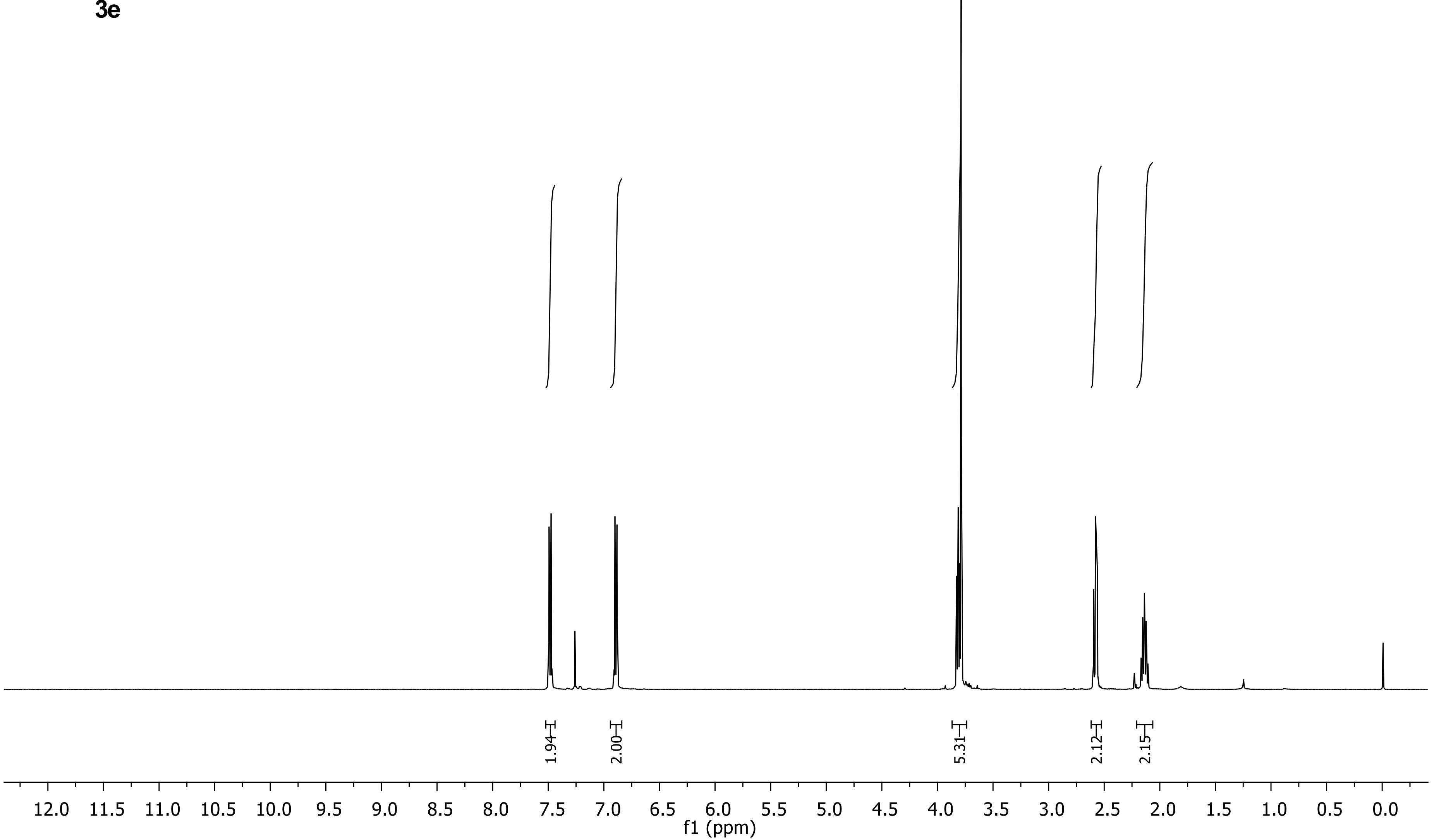
1.94
2.00
5.31
2.12
2.15
12.0 11.5 11.0 10.5 10.0 9.5 9.0 8.5 8.0 7.5 7.0 6.5 6.0 5.5 5.0 4.5 4.0 3.5 3.0 2.5 2.0 1.5 1.0 0.5 0.0
f1 (ppm)

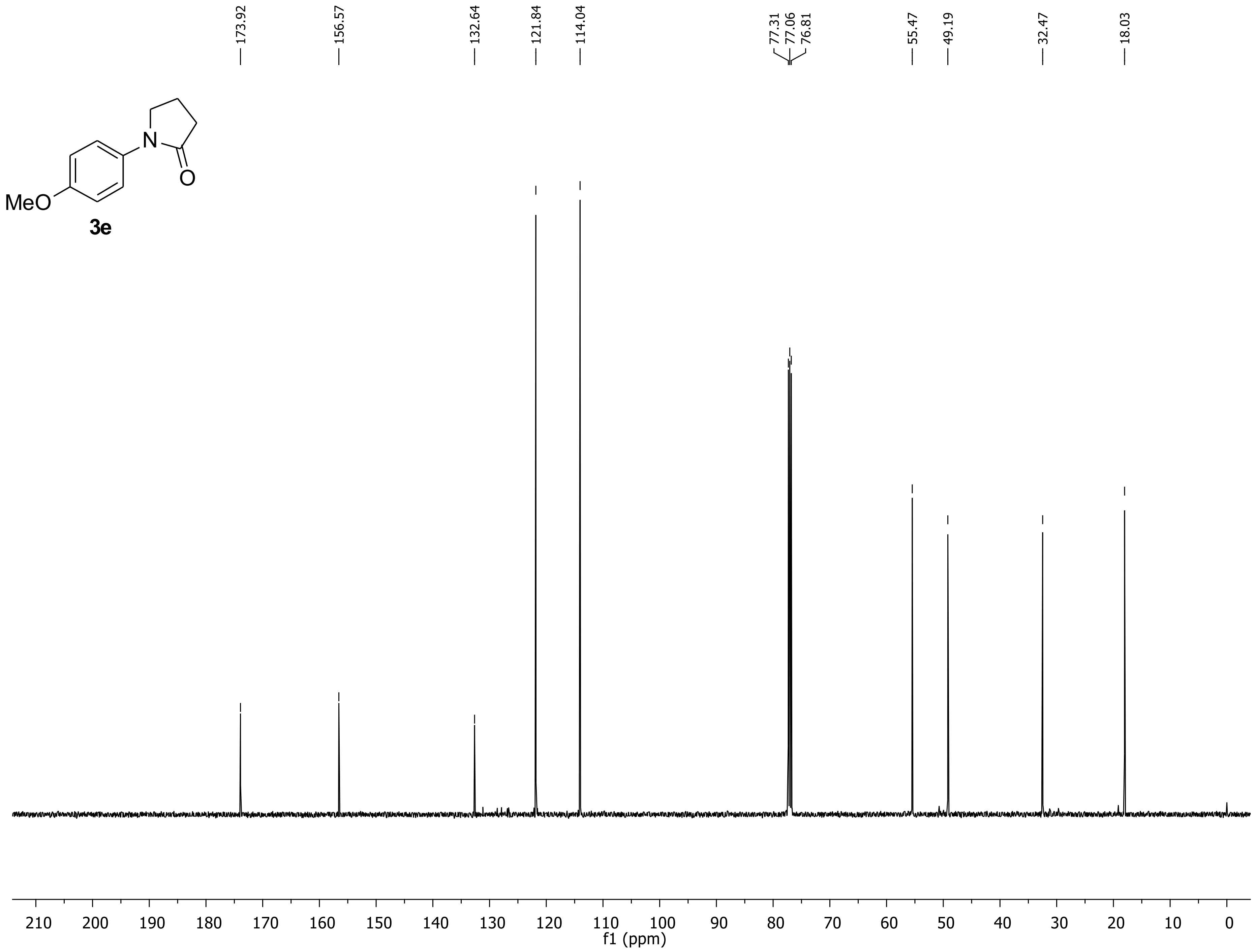
173.92
156.57
132.64
121.84
114.04
77.31
77.06
76.81
55.47
49.19
32.47
18.03
MeO
N
O
3e
210 200 190 180 170 160 150 140 130 120 110 100 90 80 70 60 50 40 30 20 10 0
f1 (ppm)

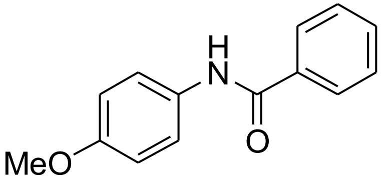

3f

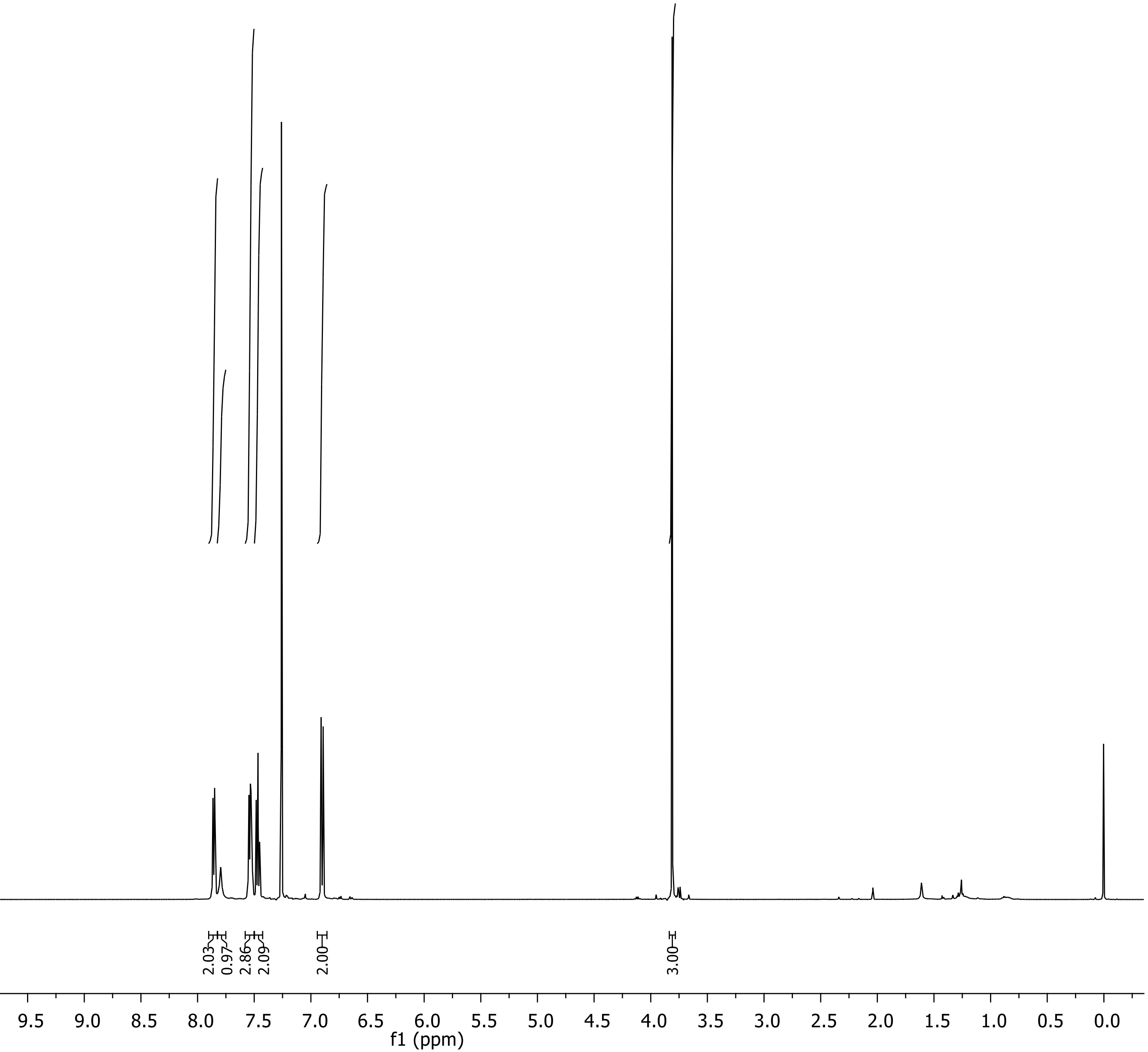
2.03
0.97
2.86
2.09
2.00
3.00
12.0 11.5 11.0 10.5 10.0 9.5 9.0 8.5 8.0 7.5 7.0 6.5 6.0 5.5 5.0 4.5 4.0 3.5 3.0 2.5 2.0 1.5 1.0 0.5 0.0
f1 (ppm)

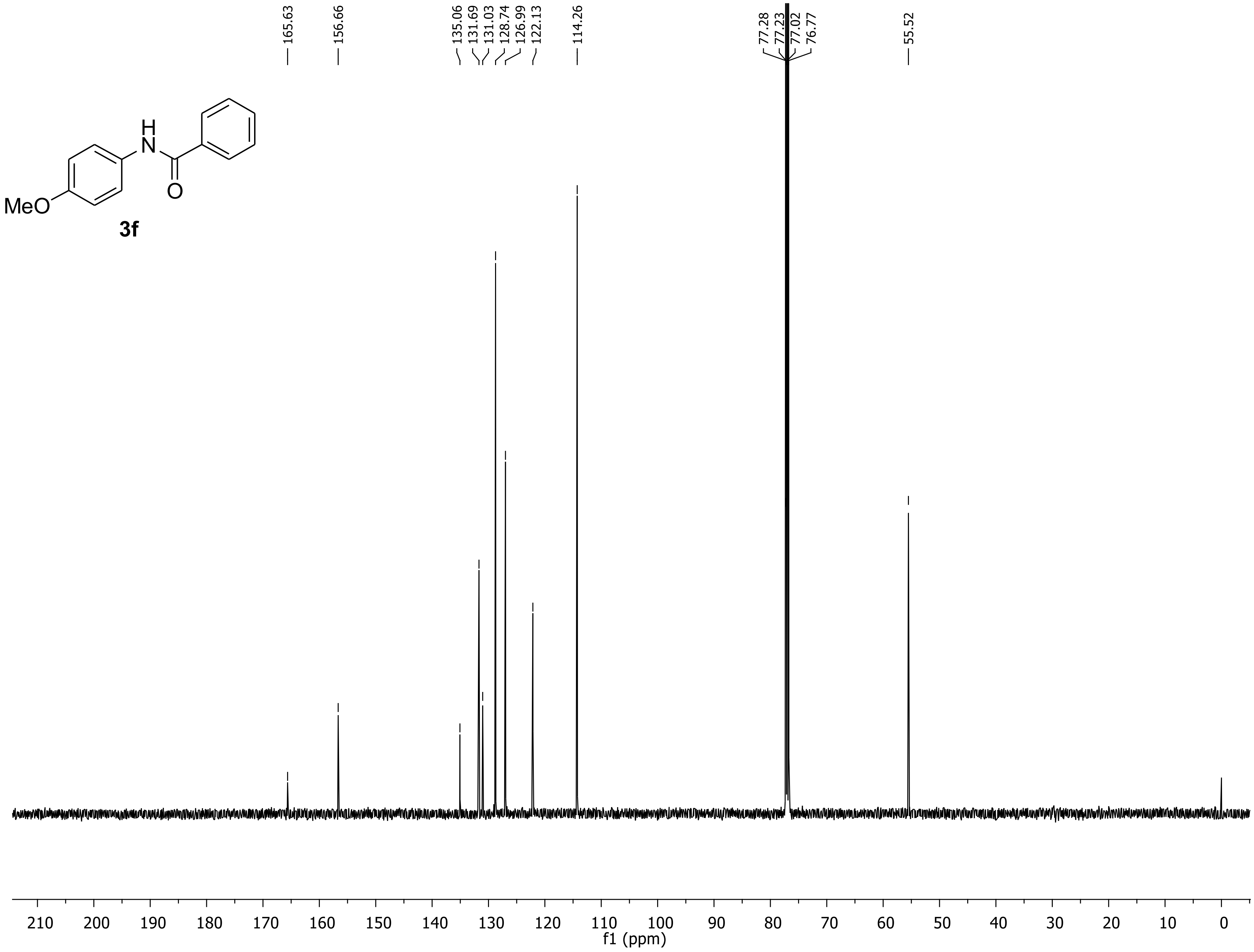
165.63
156.66
135.06
131.69
131.03
128.74
126.99
122.13
114.26
77.28
77.23
77.02
76.77
55.52
MeO
H
N
O
3f
210
200
190
180
170
160
150
140
130
120
110
100
90
80
70
60
50
40
30
20
10
0
f1 (ppm)

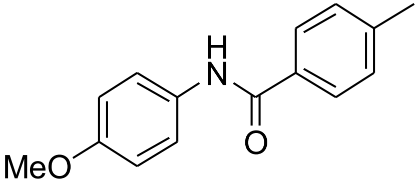

3g

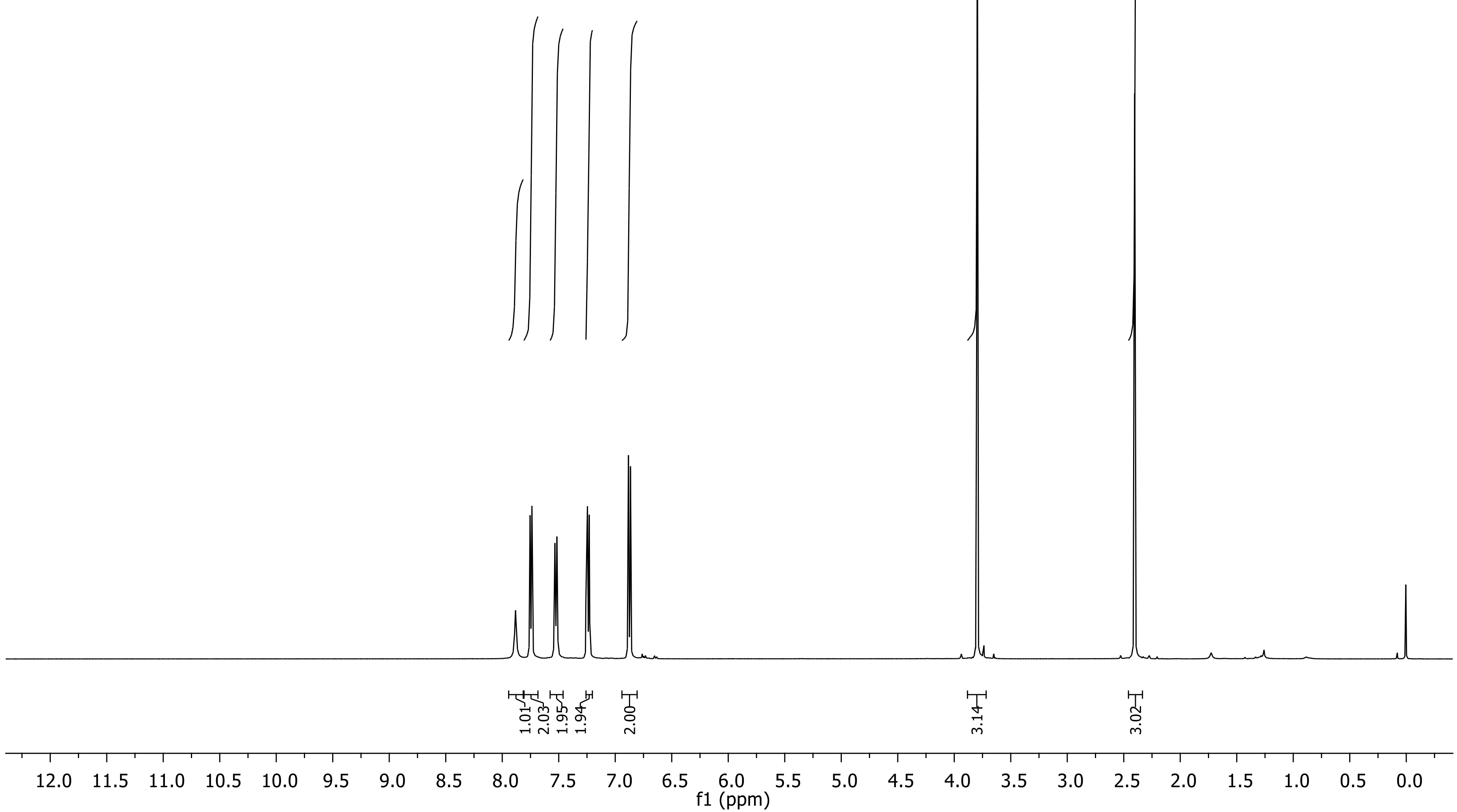

1.01
2.03
1.95
1.94
2.00
3.14
3.02
12.0 11.5 11.0 10.5 10.0 9.5 9.0 8.5 8.0 7.5 7.0 6.5 6.0 5.5 5.0 4.5 4.0 3.5 3.0 2.5 2.0 1.5 1.0 0.5 0.0
f1 (ppm)

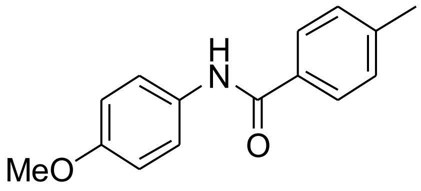
H
N
O
MeO

3g

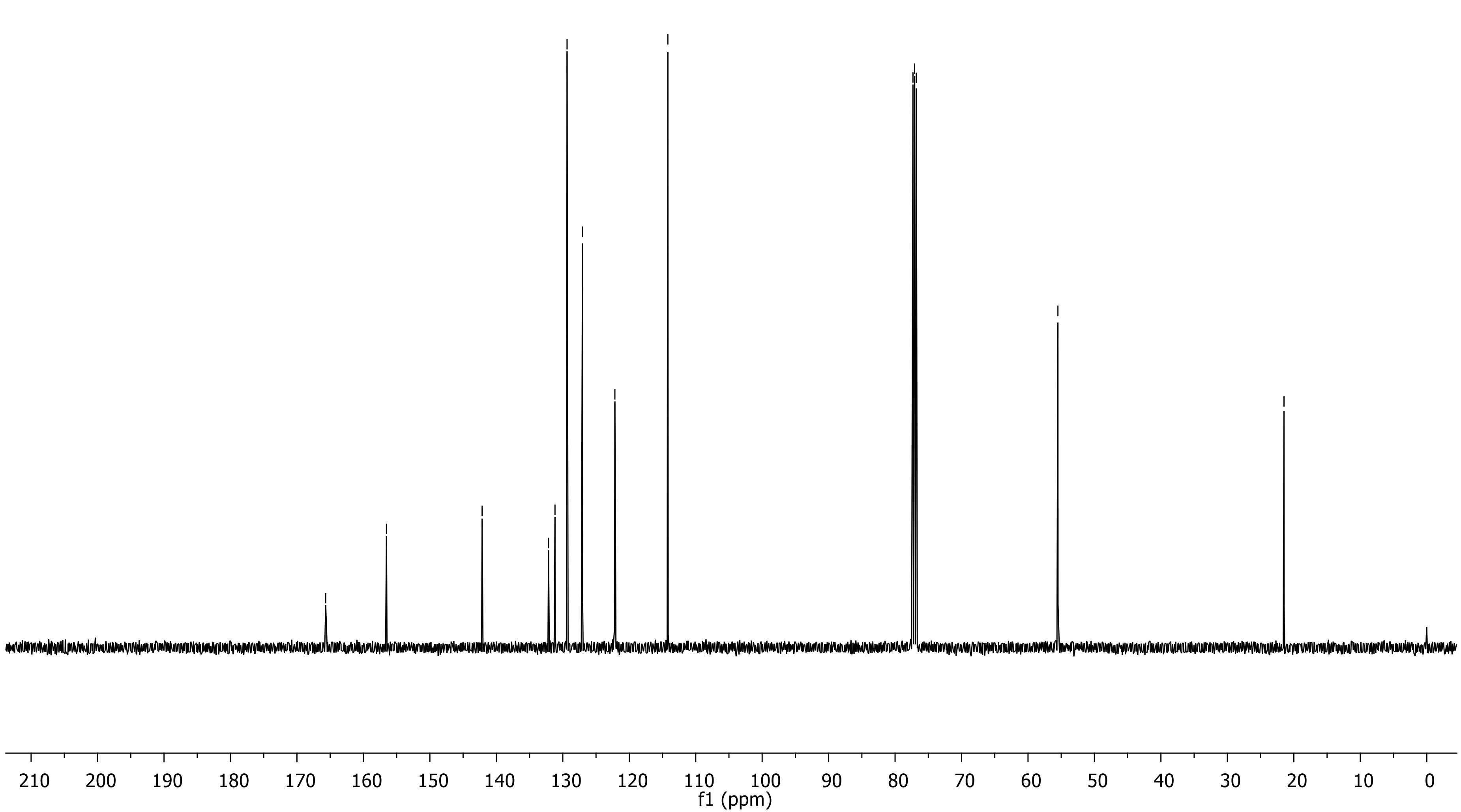
165.67
156.53
142.14
132.15
131.17
129.35
127.04
122.16
114.19
77.31
77.06
76.80
55.50
21.48
210 200 190 180 170 160 150 140 130 120 110 100 90 80 70 60 50 40 30 20 10 0
f1 (ppm)

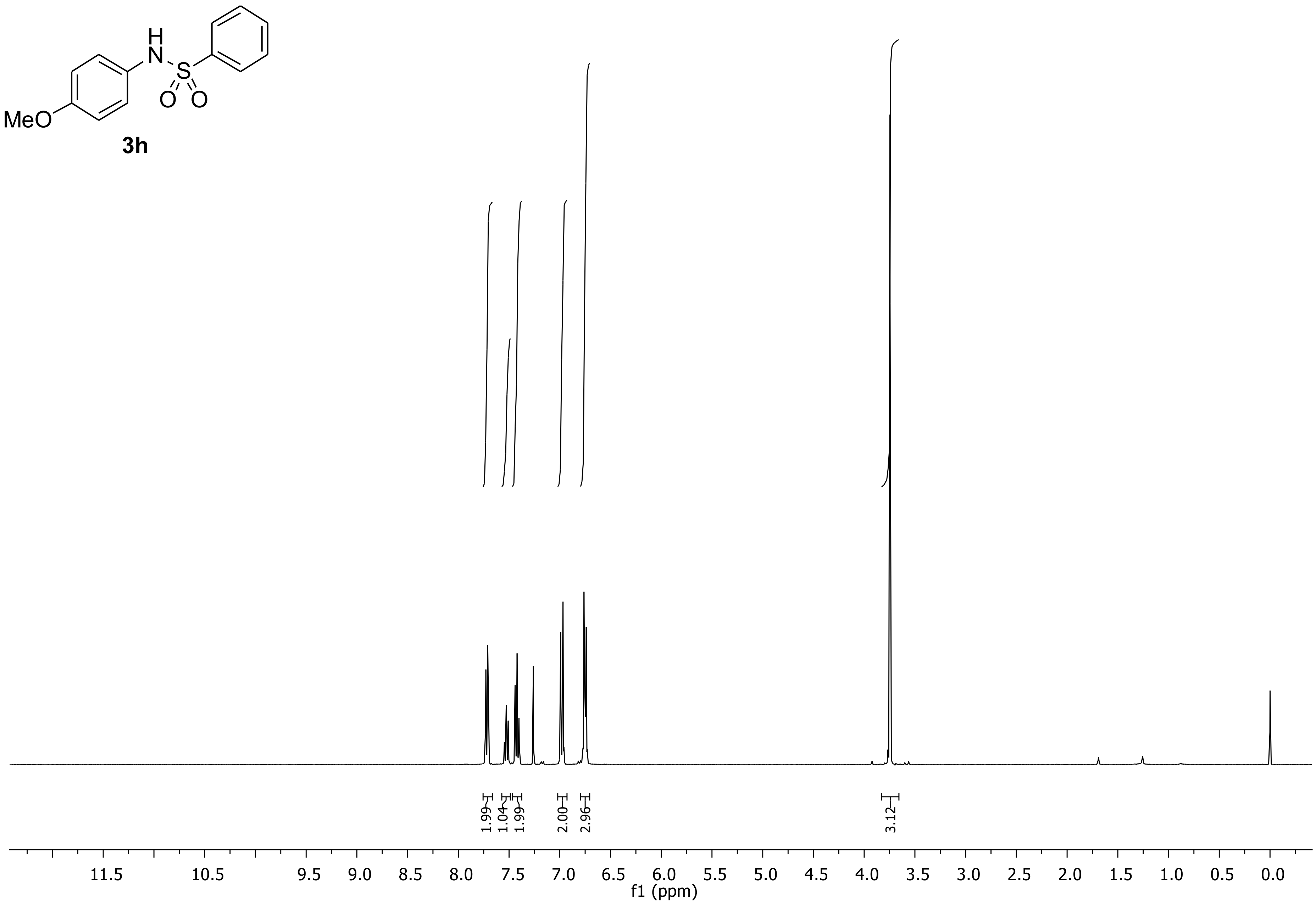
MeO
3h
1.99
1.04
1.99
2.00
2.96
3.12
11.5
10.5
9.5
9.0
8.5
8.0
7.5
7.0
6.5
6.0
5.5
5.0
4.5
4.0
3.5
3.0
2.5
2.0
1.5
1.0
0.5
0.0
f1 (ppm)

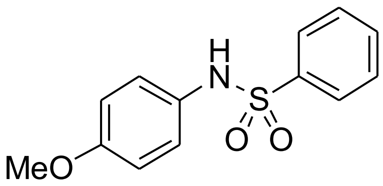
H
N
S
O
O
MeO

3h

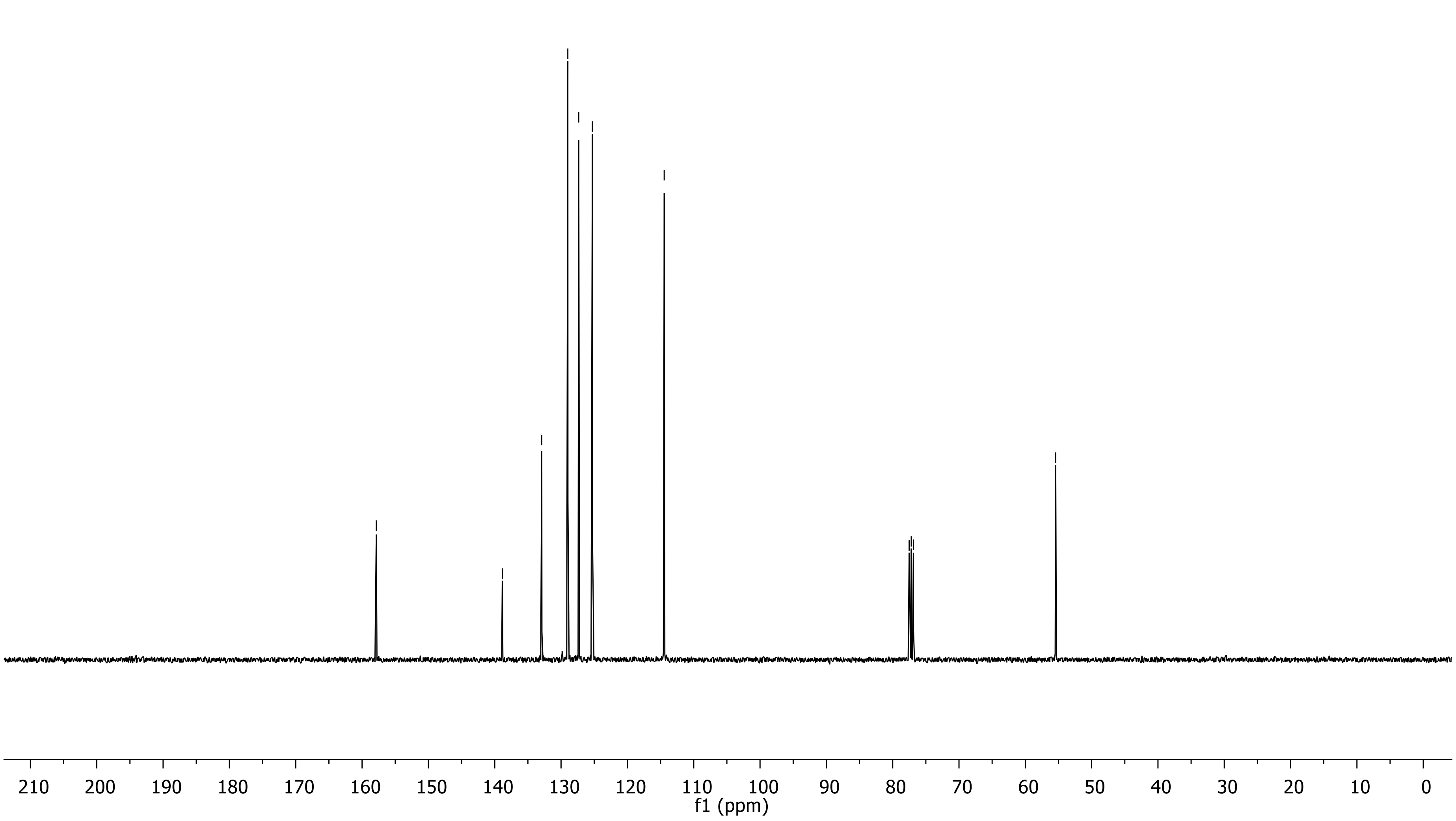
157.86
138.86
132.91
128.98
127.33
125.27
114.45
77.51
77.19
76.88
55.42
210 200 190 180 170 160 150 140 130 120 110 100 90 80 70 60 50 40 30 20 10 0
f1 (ppm)

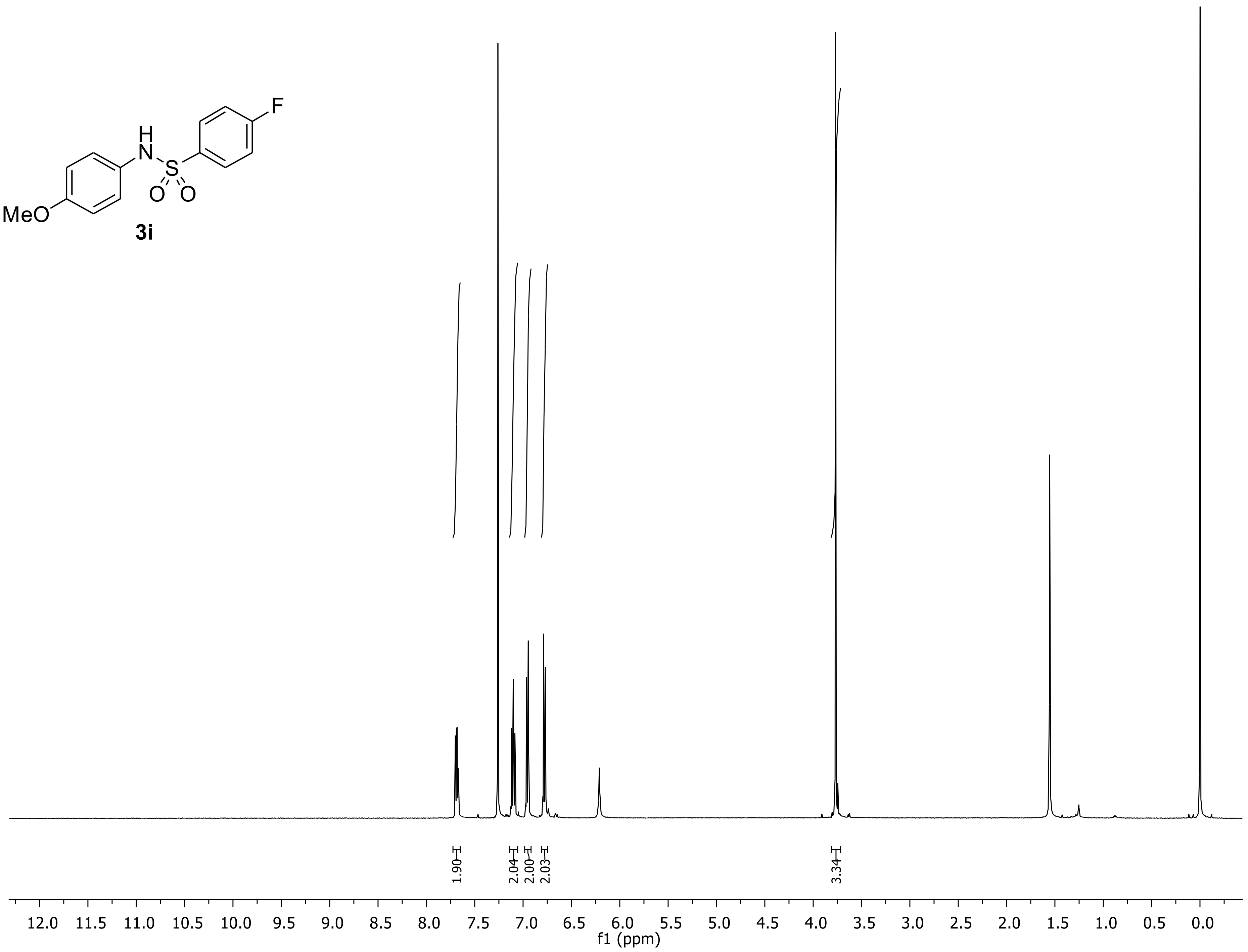
F
H
N
S
O O
MeO
3i
1.90
2.04
2.00
2.03
3.34
12.0 11.5 11.0 10.5 10.0 9.5 9.0 8.5 8.0 7.5 7.0 6.5 6.0 5.5 5.0 4.5 4.0 3.5 3.0 2.5 2.0 1.5 1.0 0.5 0.0
f1 (ppm)

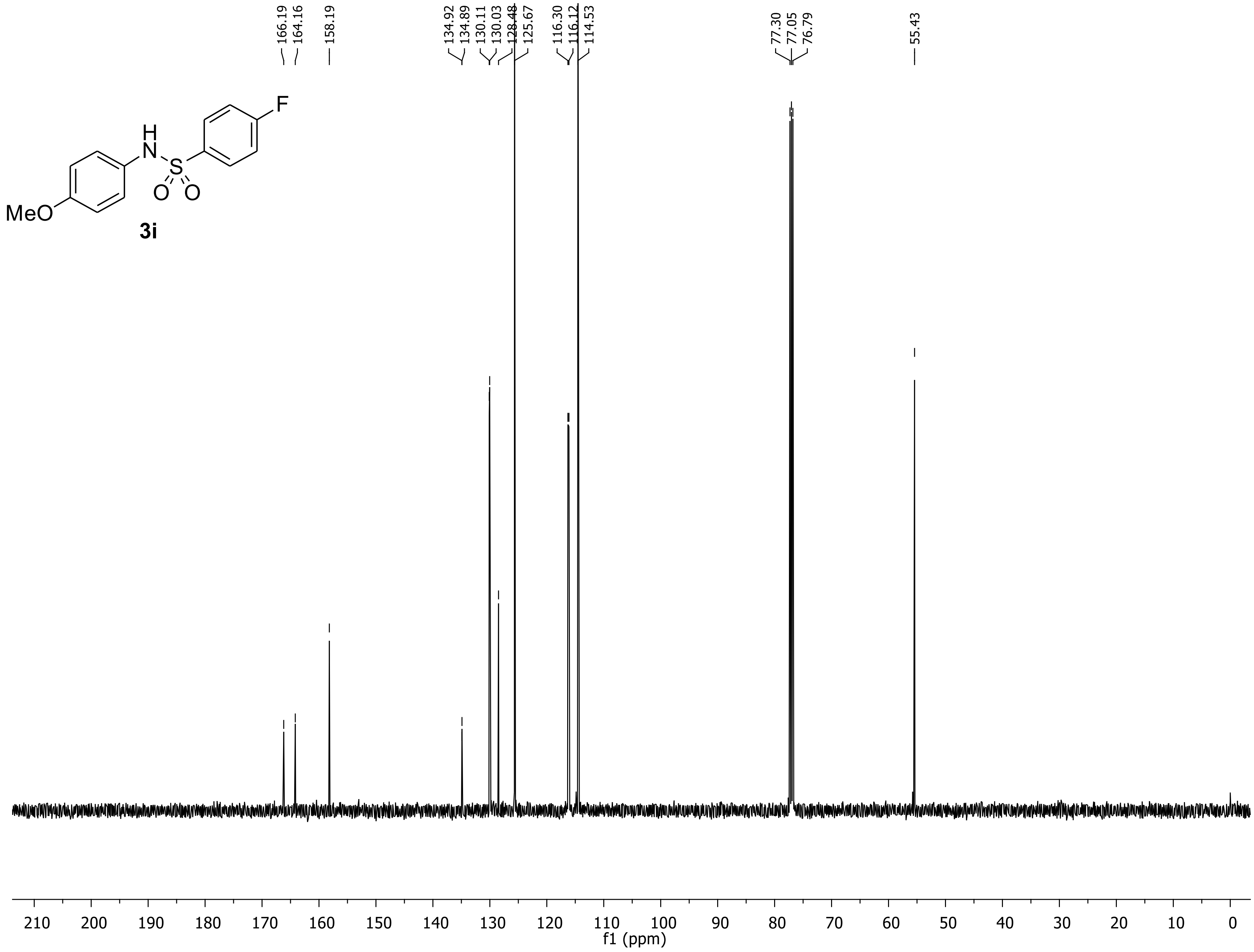
166.19
164.16
158.19
134.92
134.89
130.11
130.03
128.48
125.67
116.30
116.12
114.53
77.30
77.05
76.79
55.43
MeO
H
N
S
O
O
F
3i
210 200 190 180 170 160 150 140 130 120 110 100 90 80 70 60 50 40 30 20 10 0
f1 (ppm)

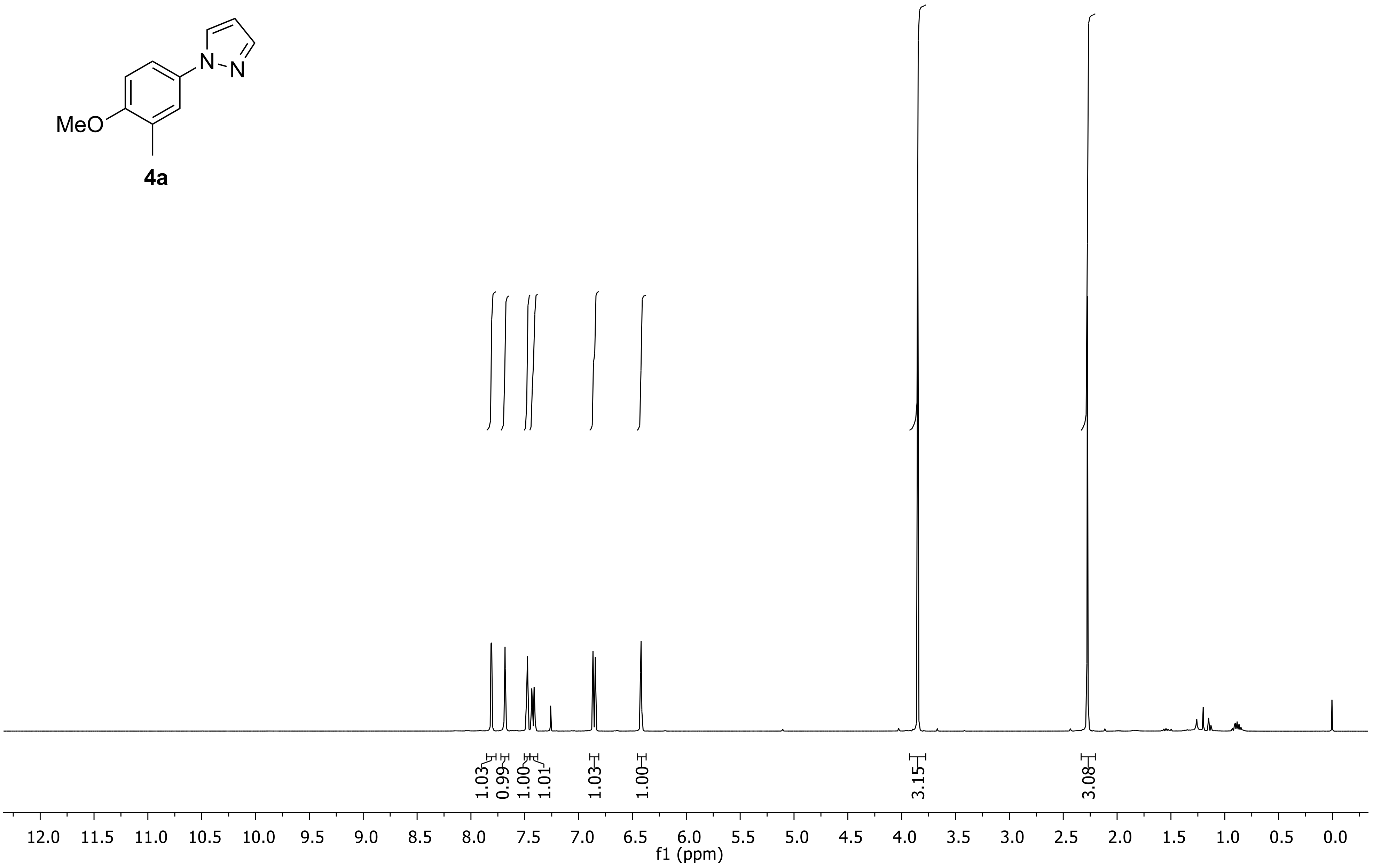
MeO
N
N
4a
1.03
0.99
1.00
1.01
1.03
1.00
3.15
3.08
12.0
11.5
11.0
10.5
10.0
9.5
9.0
8.5
8.0
7.5
7.0
6.5
6.0
5.5
5.0
4.5
4.0
3.5
3.0
2.5
2.0
1.5
1.0
0.5
0.0
f1 (ppm)

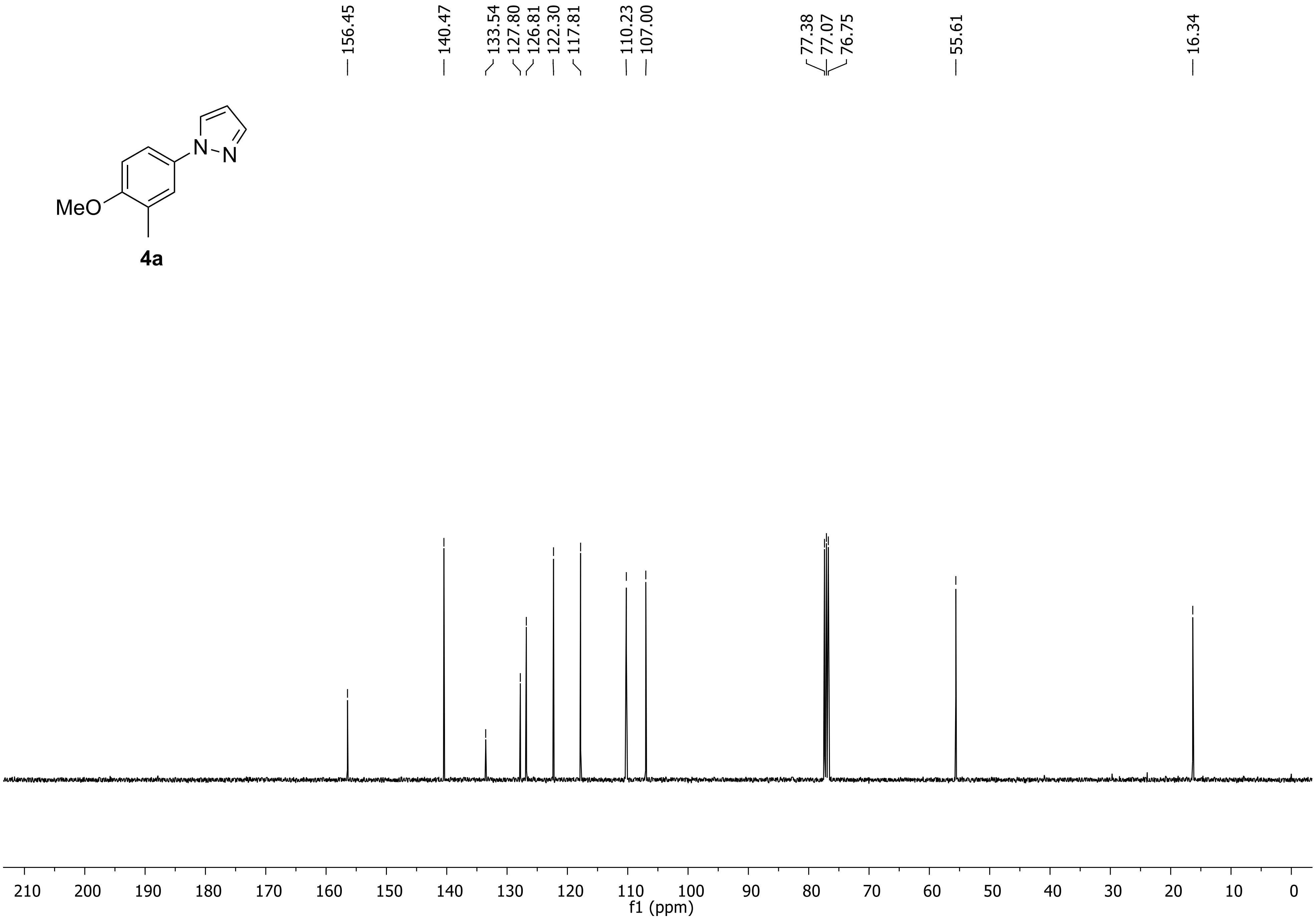

156.45
140.47
133.54
127.80
126.81
122.30
117.81
110.23
107.00
77.38
77.07
76.75
55.61
16.34
MeO
N
N
210 200 190 180 170 160 150 140 130 120 110 100 90 80 70 60 50 40 30 20 10 0
f1 (ppm)

4a

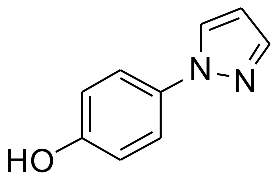
HO
N
N

4b

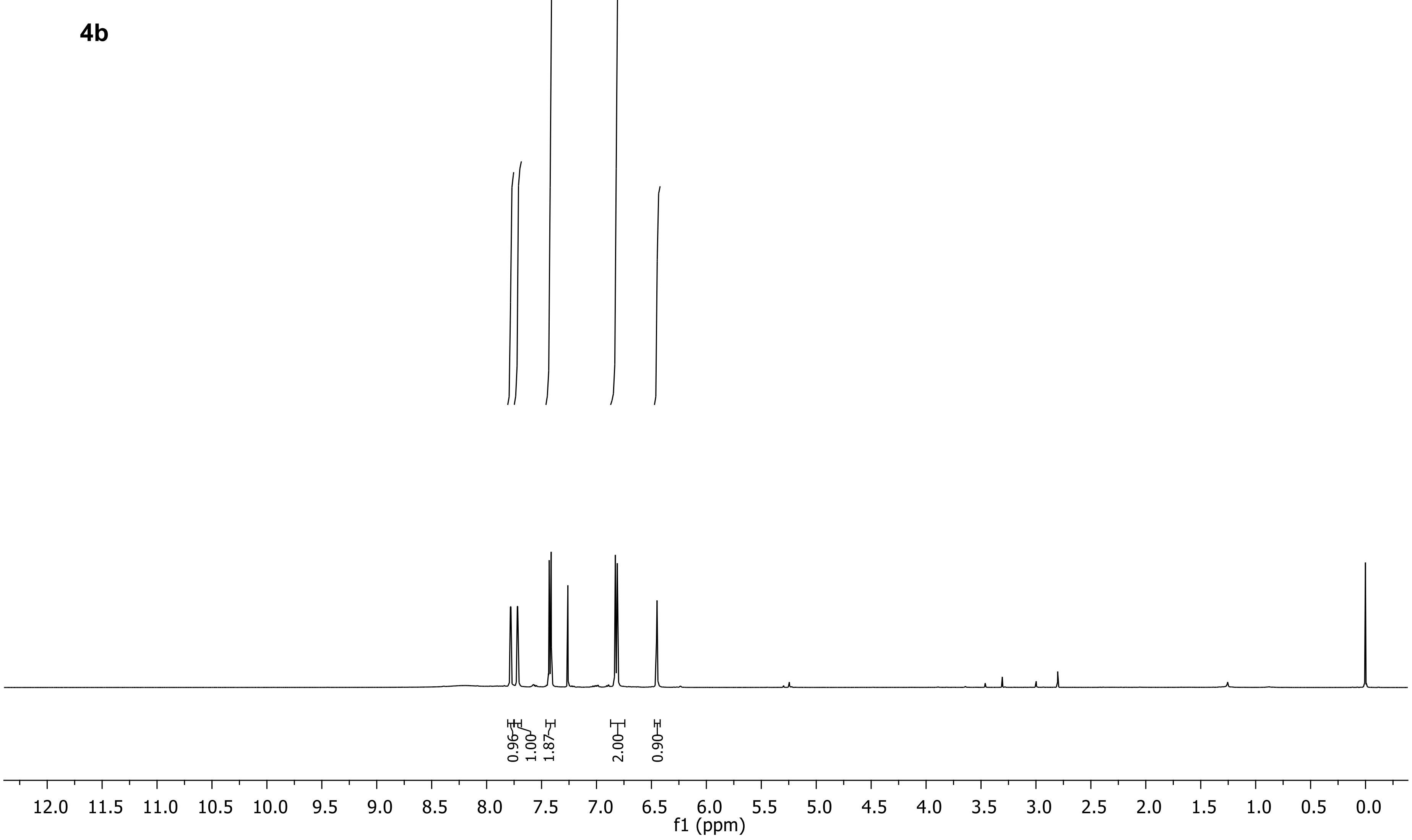
0.96
1.00
1.87
2.00
0.90
12.0 11.5 11.0 10.5 10.0 9.5 9.0 8.5 8.0 7.5 7.0 6.5 6.0 5.5 5.0 4.5 4.0 3.5 3.0 2.5 2.0 1.5 1.0 0.5 0.0
f1 (ppm)

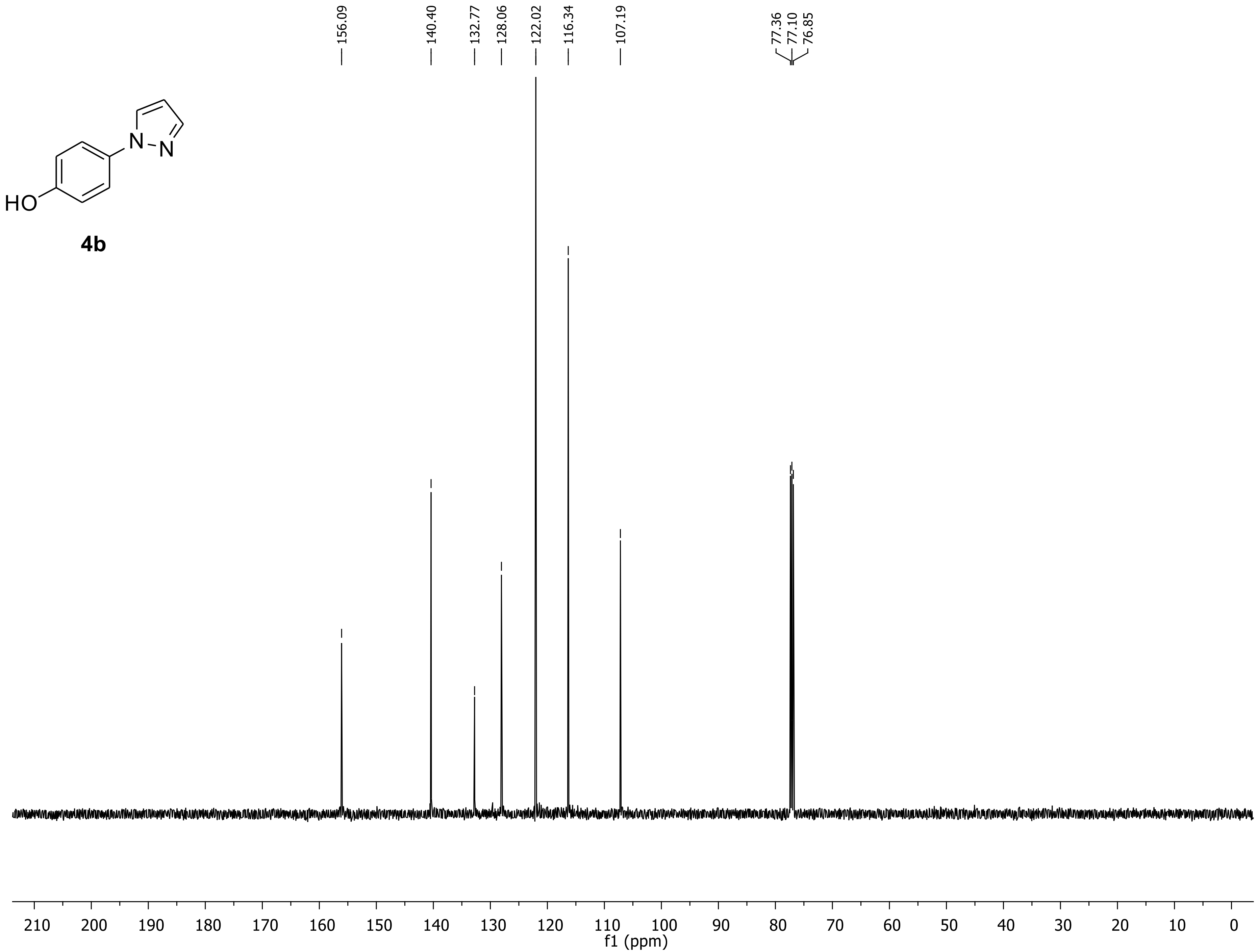
156.09
140.40
132.77
128.06
122.02
116.34
107.19
77.36
77.10
76.85
N
N
HO
4b
210 200 190 180 170 160 150 140 130 120 110 100 90 80 70 60 50 40 30 20 10 0
f1 (ppm)

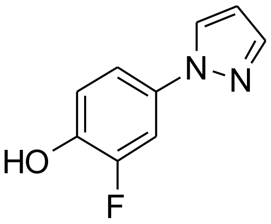
HO
F
N
N

4c

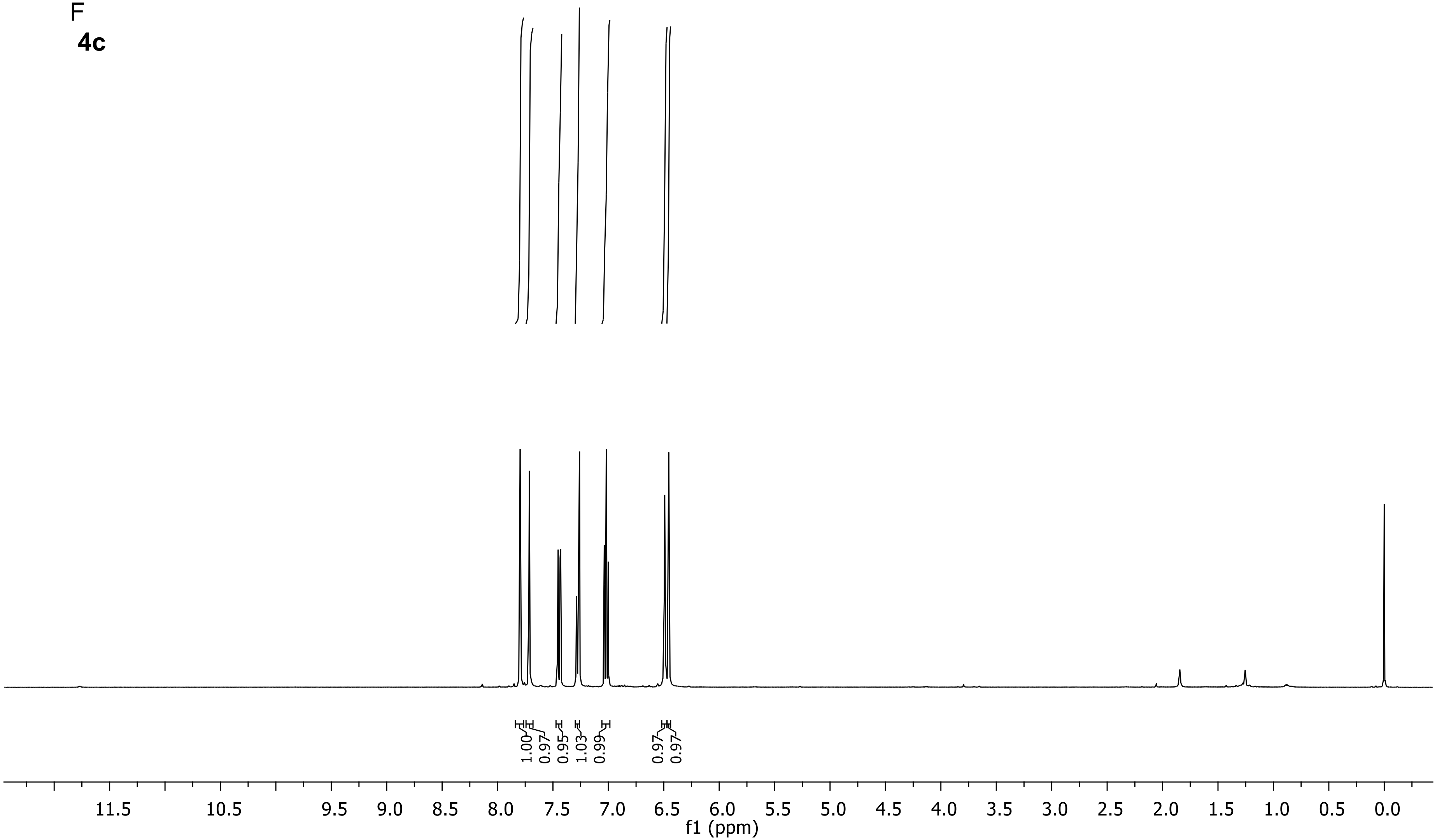
1.00
0.97
0.95
1.03
0.99
0.97
0.97
11.5
10.5
9.5
9.0
8.5
8.0
7.5
7.0
6.5
6.0
5.5
5.0
4.5
4.0
3.5
3.0
2.5
2.0
1.5
1.0
0.5
0.0
f1 (ppm)

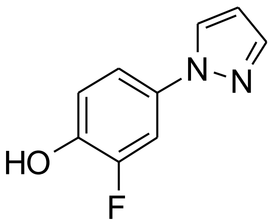
HO
F
N
N

4c

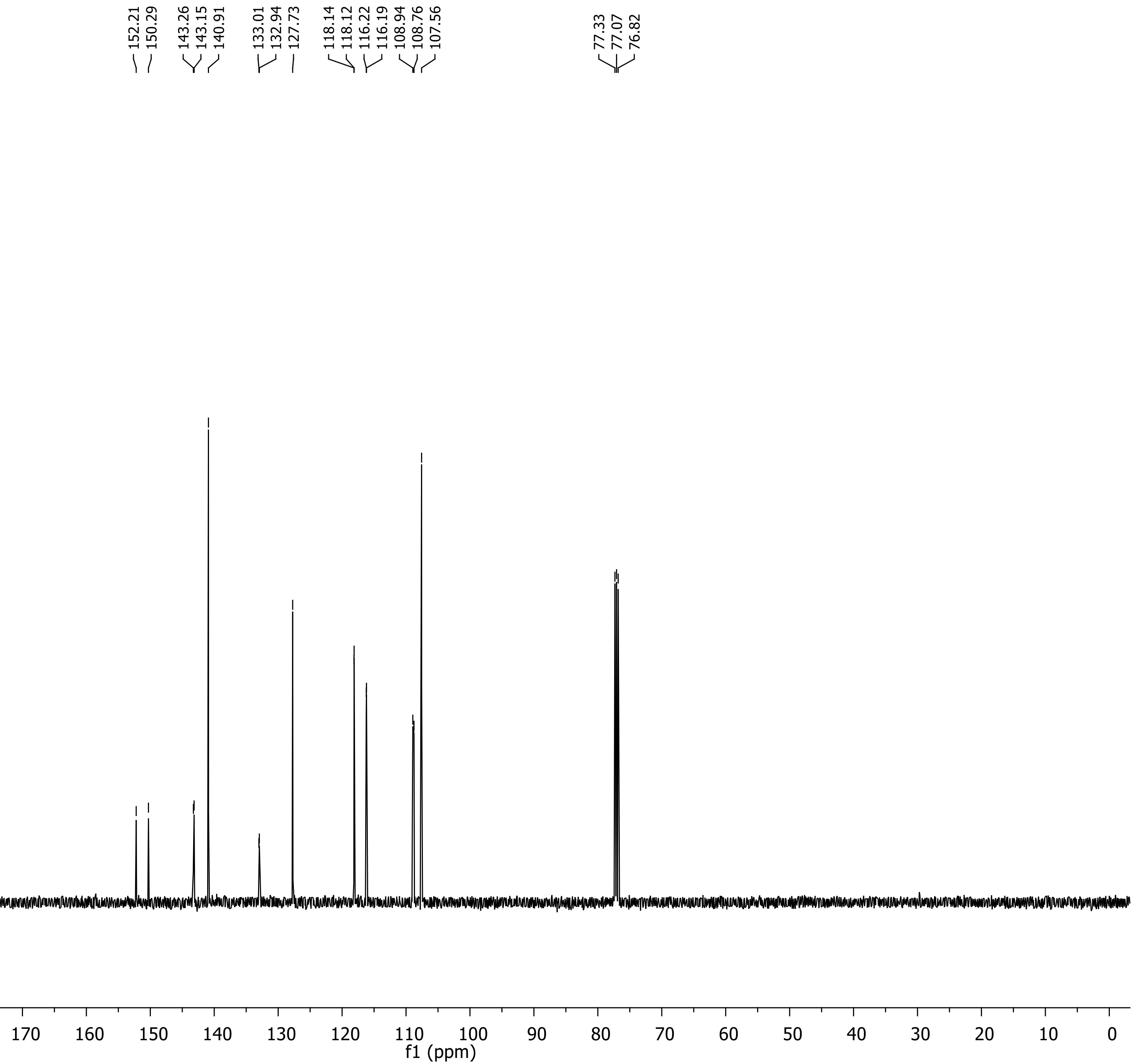
152.21
150.29
143.26
143.15
140.91
133.01
132.94
127.73
118.14
118.12
116.22
116.19
108.94
108.76
107.56
77.33
77.07
76.82
210 200 190 180 170 160 150 140 130 120 110 100 90 80 70 60 50 40 30 20 10 0
f1 (ppm)

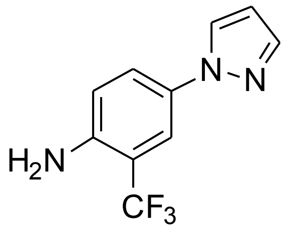
H2N
N
N
CF3

4d

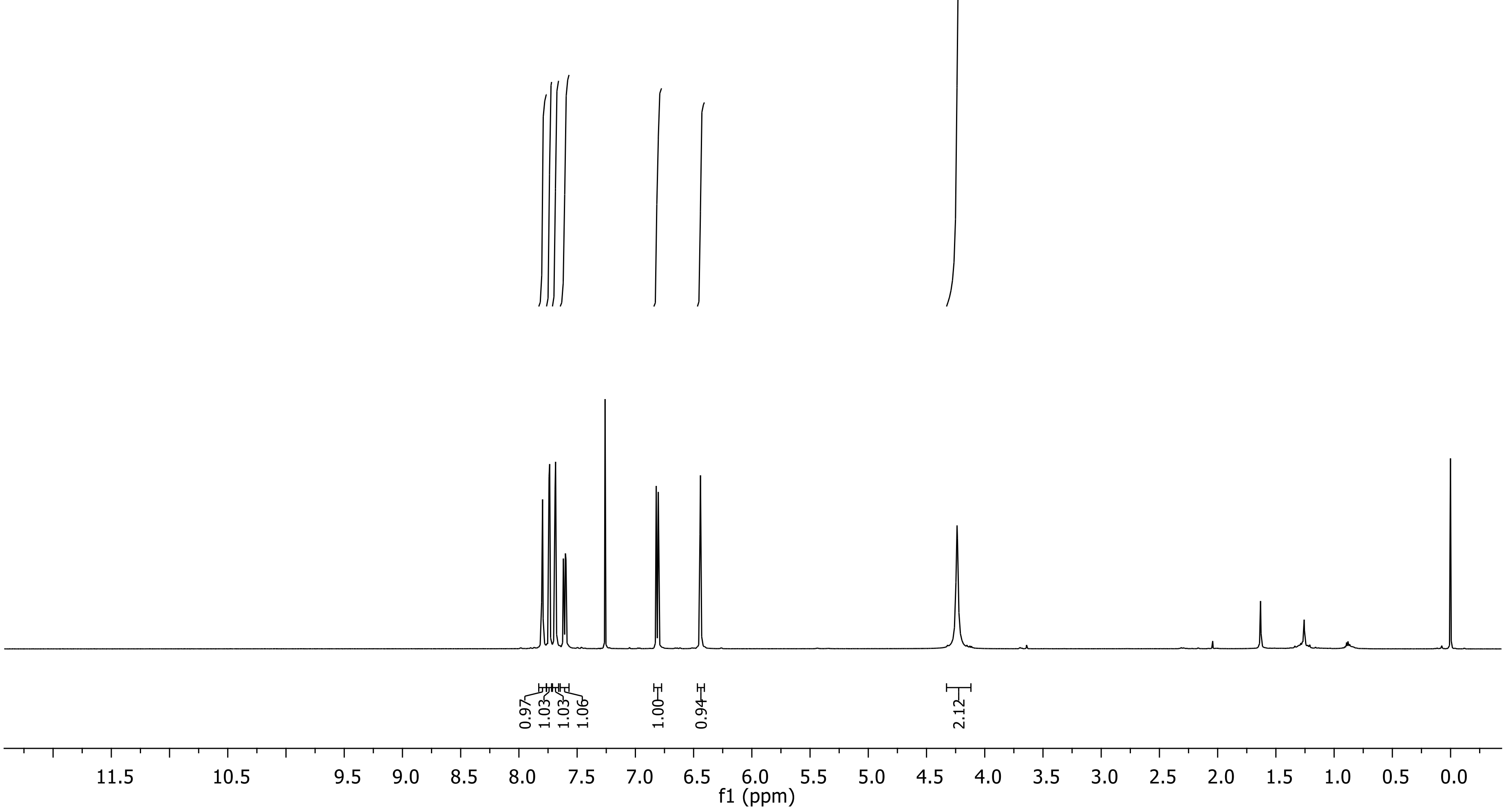
0.97
1.03
1.03
1.06
1.00
0.94
2.12
11.5
10.5
9.5
9.0
8.5
8.0
7.5
7.0
6.5
6.0
5.5
5.0
4.5
4.0
3.5
3.0
2.5
2.0
1.5
1.0
0.5
0.0
f1 (ppm)

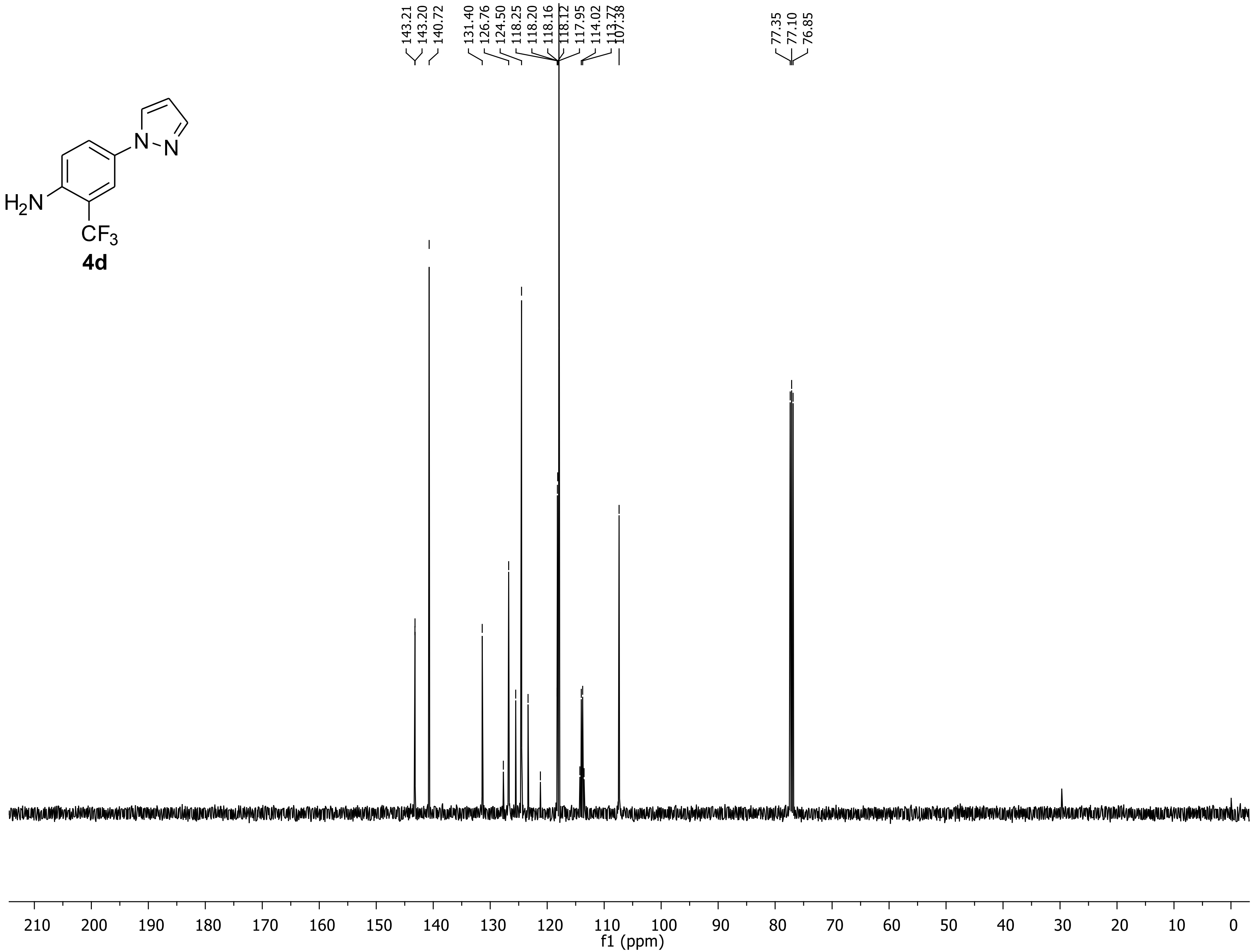
143.21
143.20
140.72
131.40
126.76
124.50
118.25
118.20
118.16
118.12
117.95
114.02
113.77
107.38
77.35
77.10
76.85
H_2N
N
N
CF_3
4d
210 200 190 180 170 160 150 140 130 120 110 100 90 80 70 60 50 40 30 20 10 0
f1 (ppm)

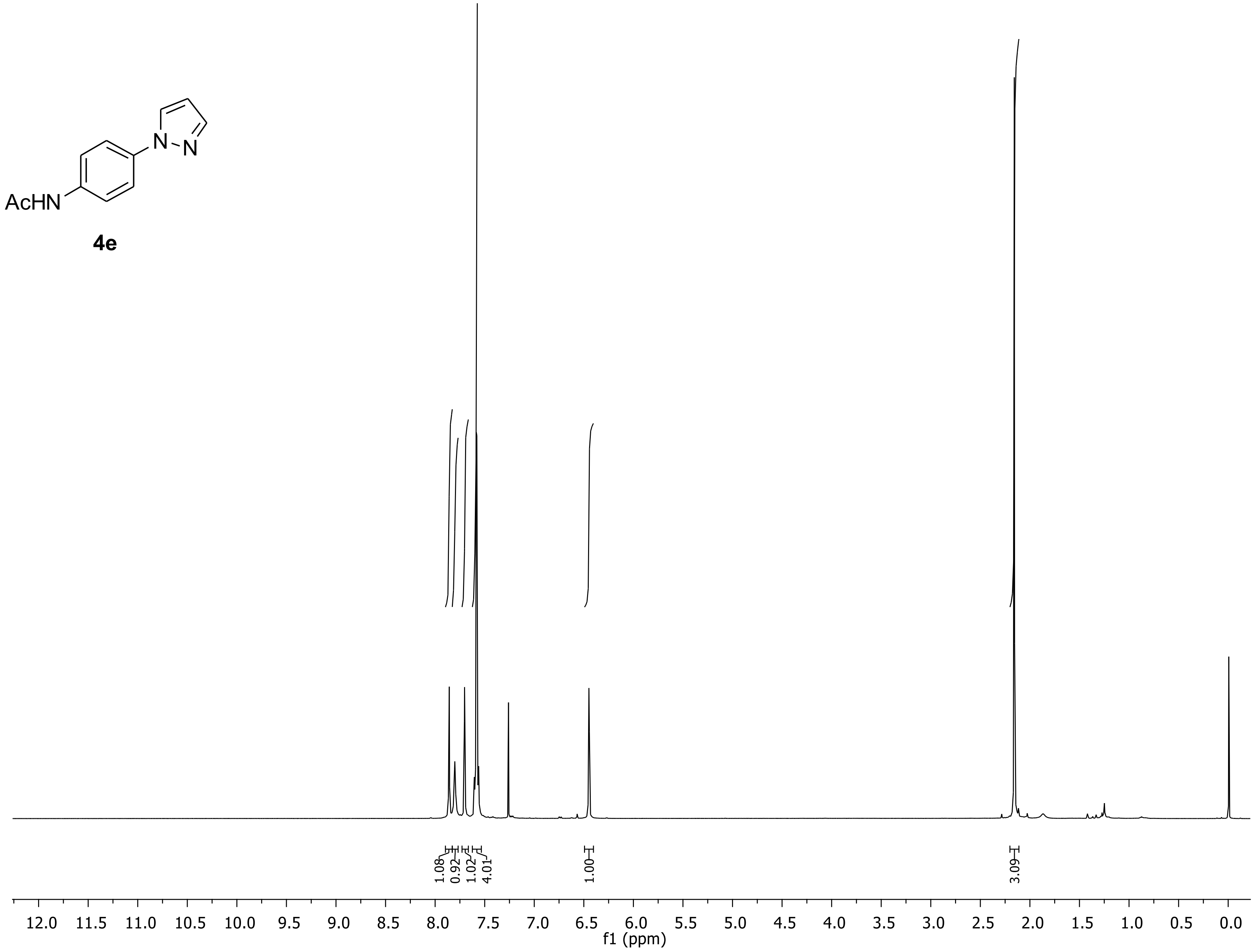
AcHN
N
N
4e
1.08
0.92
1.02
4.01
1.00
3.09
12.0 11.5 11.0 10.5 10.0 9.5 9.0 8.5 8.0 7.5 7.0 6.5 6.0 5.5 5.0 4.5 4.0 3.5 3.0 2.5 2.0 1.5 1.0 0.5 0.0
f1 (ppm)

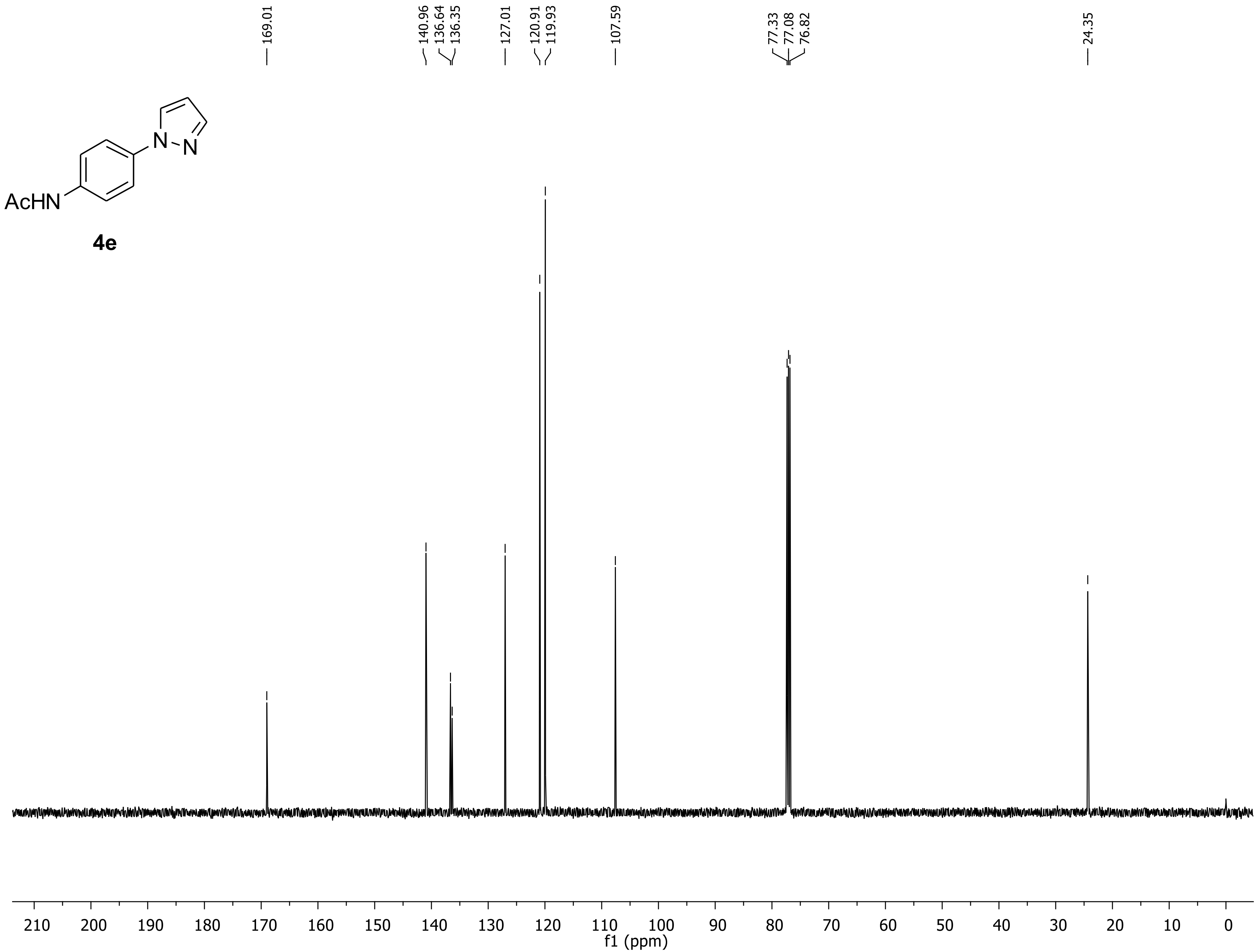
AcHN
N
N
4e
169.01
140.96
136.64
136.35
127.01
120.91
119.93
107.59
77.33
77.08
76.82
24.35
210 200 190 180 170 160 150 140 130 120 110 100 90 80 70 60 50 40 30 20 10 0
f1 (ppm)

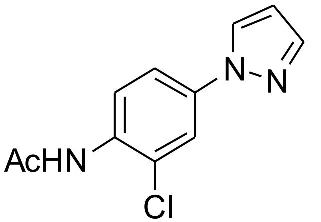
AcHN
Cl
N
N

4f

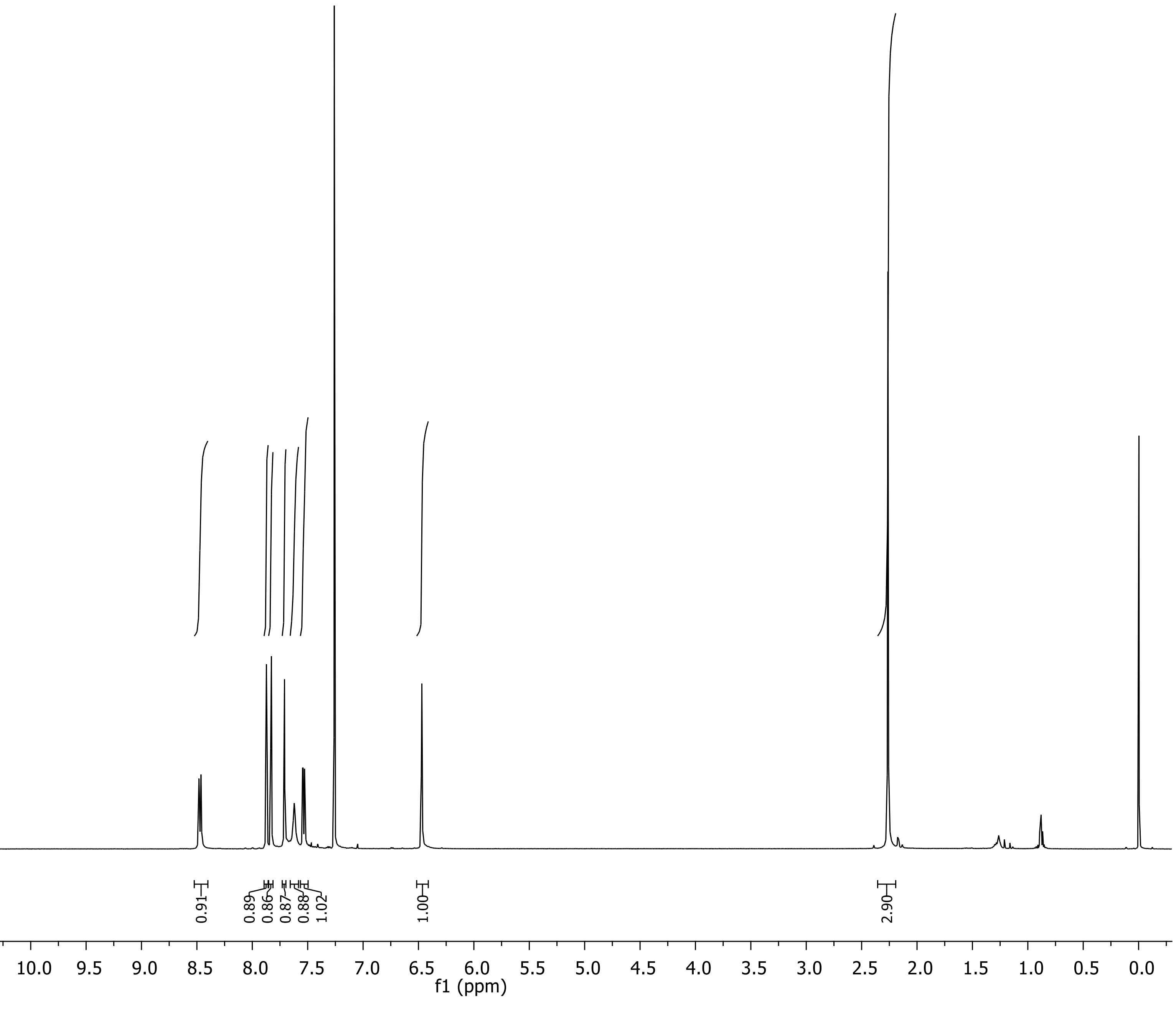
0.91
0.89
0.86
0.87
0.88
1.02
1.00
2.90
12.0 11.5 11.0 10.5 10.0 9.5 9.0 8.5 8.0 7.5 7.0 6.5 6.0 5.5 5.0 4.5 4.0 3.5 3.0 2.5 2.0 1.5 1.0 0.5 0.0
f1 (ppm)

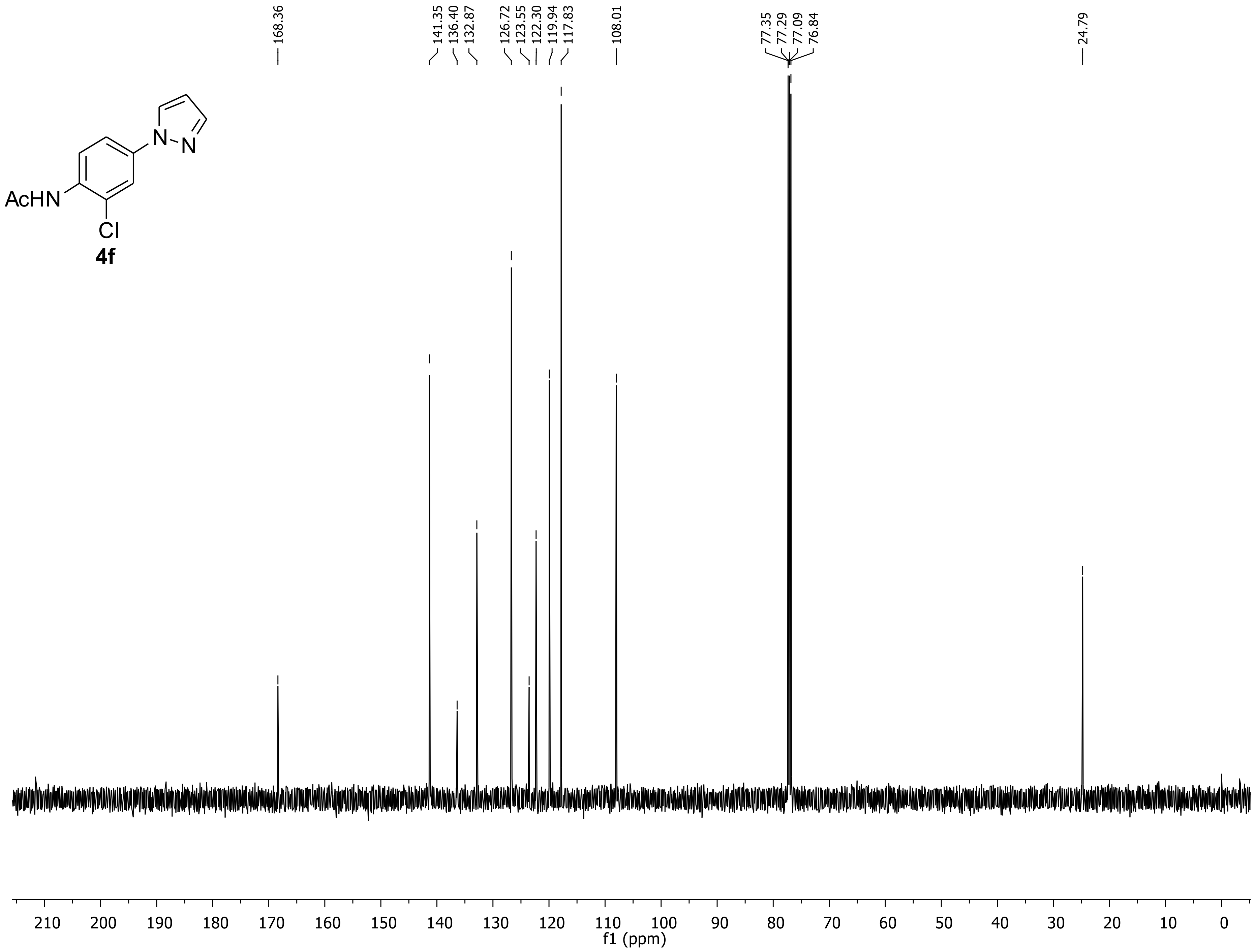
168.36
141.35
136.40
132.87
126.72
123.55
122.30
119.94
117.83
108.01
77.35
77.29
77.09
76.84
24.79
AcHN
Cl
N
N
4f
210 200 190 180 170 160 150 140 130 120 110 100 90 80 70 60 50 40 30 20 10 0
f1 (ppm)

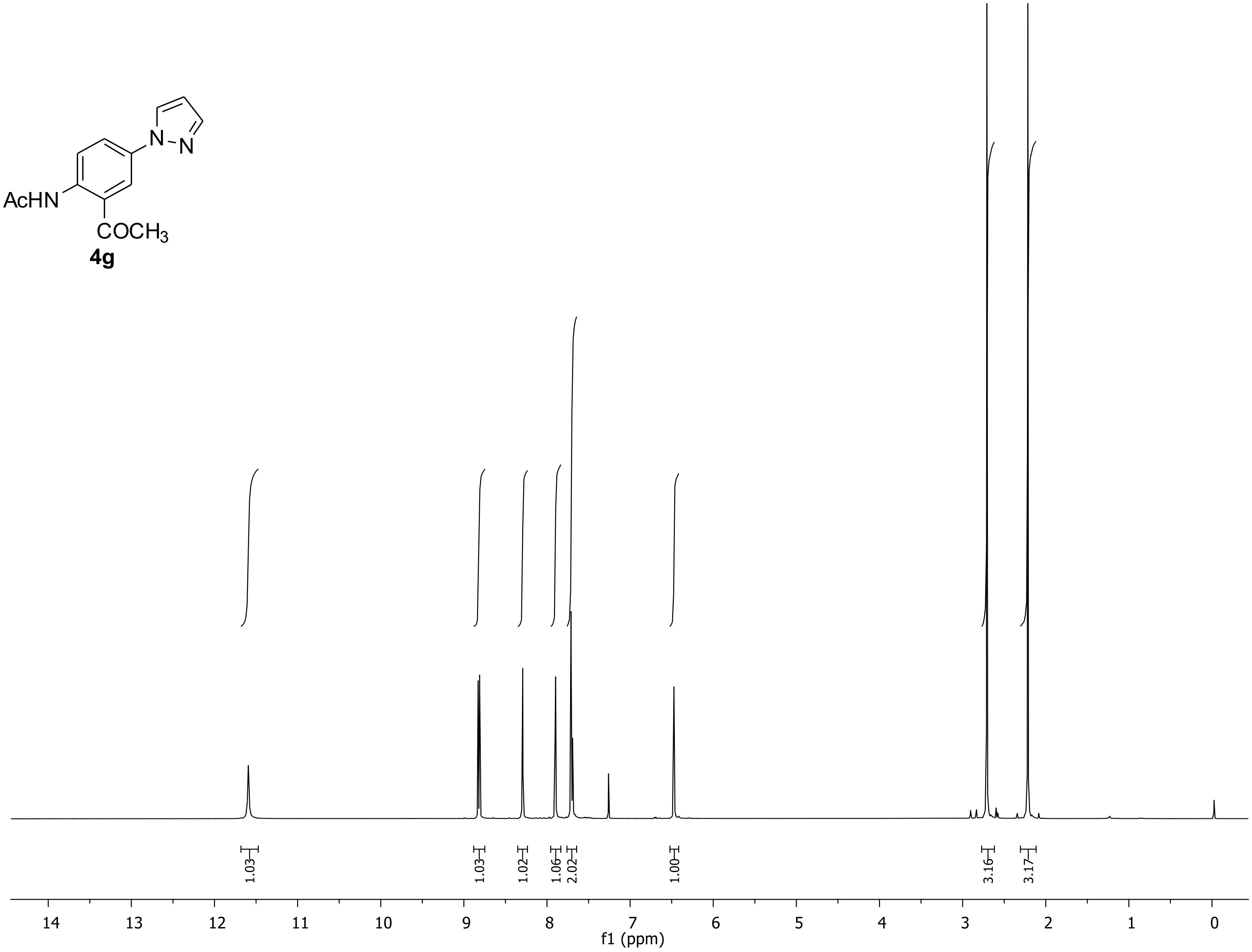
AcHN
N
N
COCH3
4g
1.03
1.03
1.02
1.06
2.02
1.00
3.16
3.17
14
13
12
11
10
9
8
7
6
5
4
3
2
1
0
f1 (ppm)

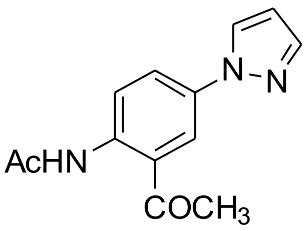
AcHN
COCH3
N
N

4g

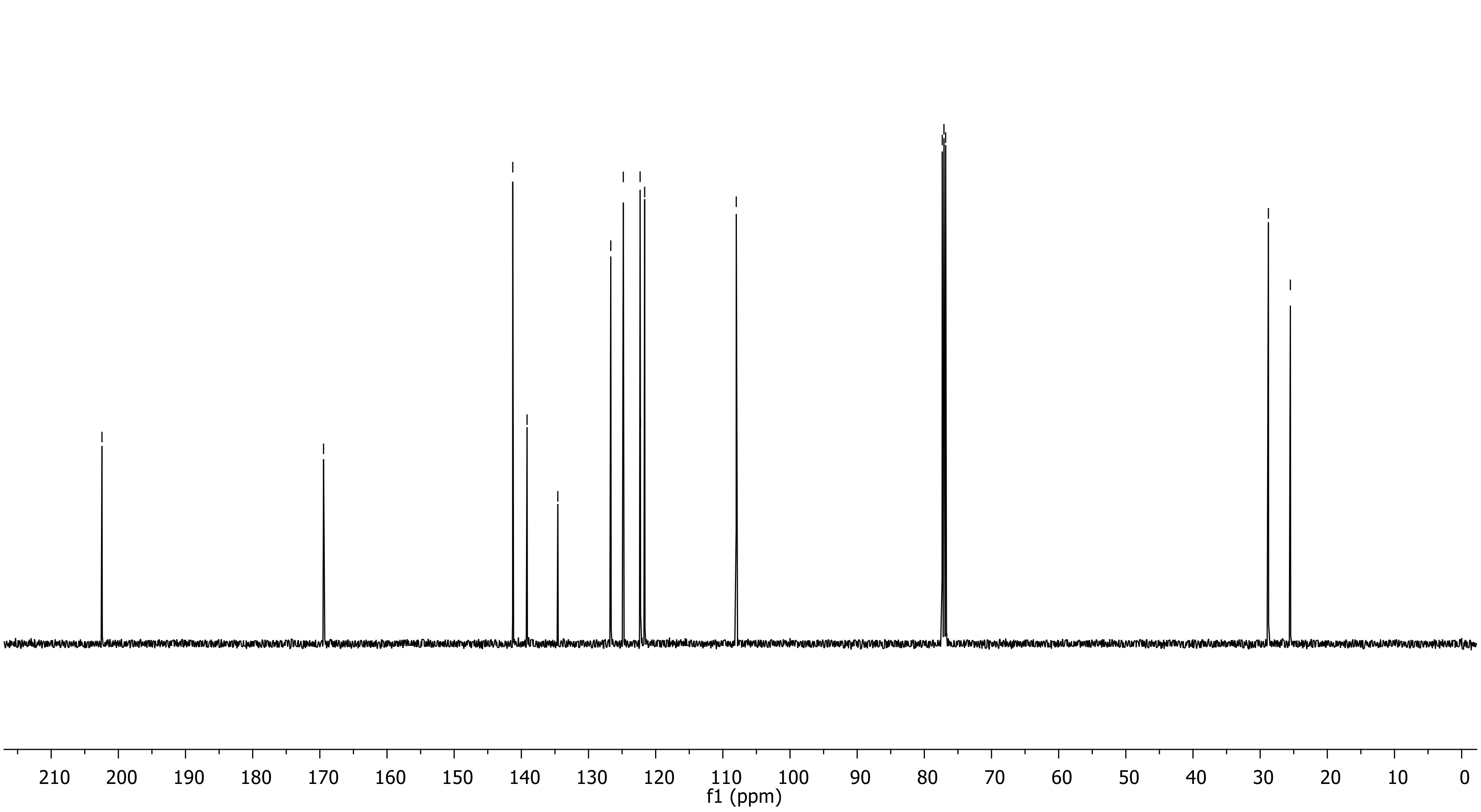
202.44
169.45
141.28
139.15
134.57
126.68
124.82
122.32
122.27
121.64
108.00
77.33
77.08
76.82
28.77
25.51
210 200 190 180 170 160 150 140 130 120 110 100 90 80 70 60 50 40 30 20 10 0
f1 (ppm)

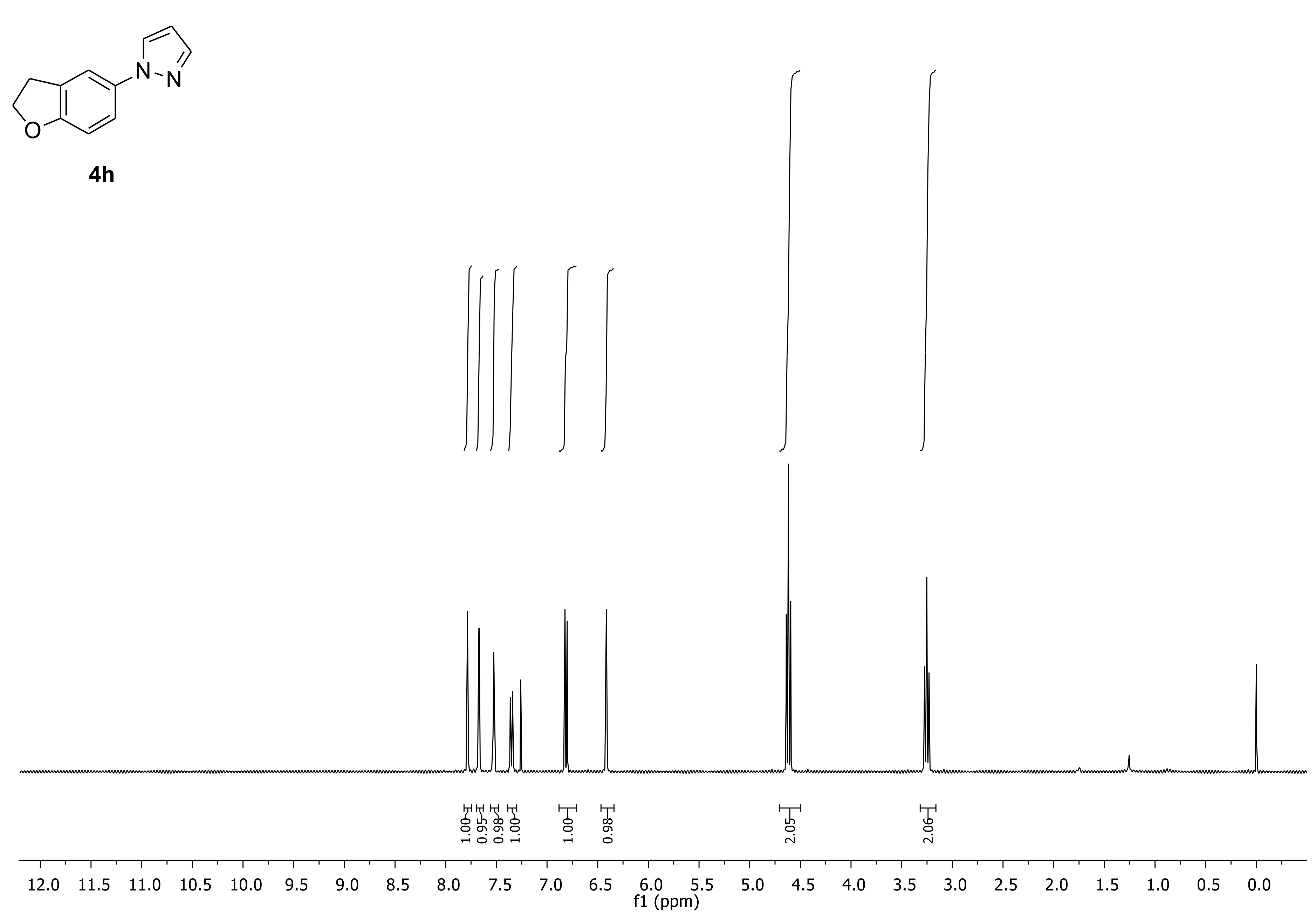

4h
1.00
0.95
0.98
1.00
1.00
0.98
2.05
2.06
12.0 11.5 11.0 10.5 10.0 9.5 9.0 8.5 8.0 7.5 7.0 6.5 6.0 5.5 5.0 4.5 4.0 3.5 3.0 2.5 2.0 1.5 1.0 0.5 0.0
f1 (ppm)

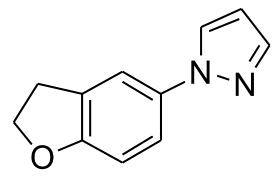

4h

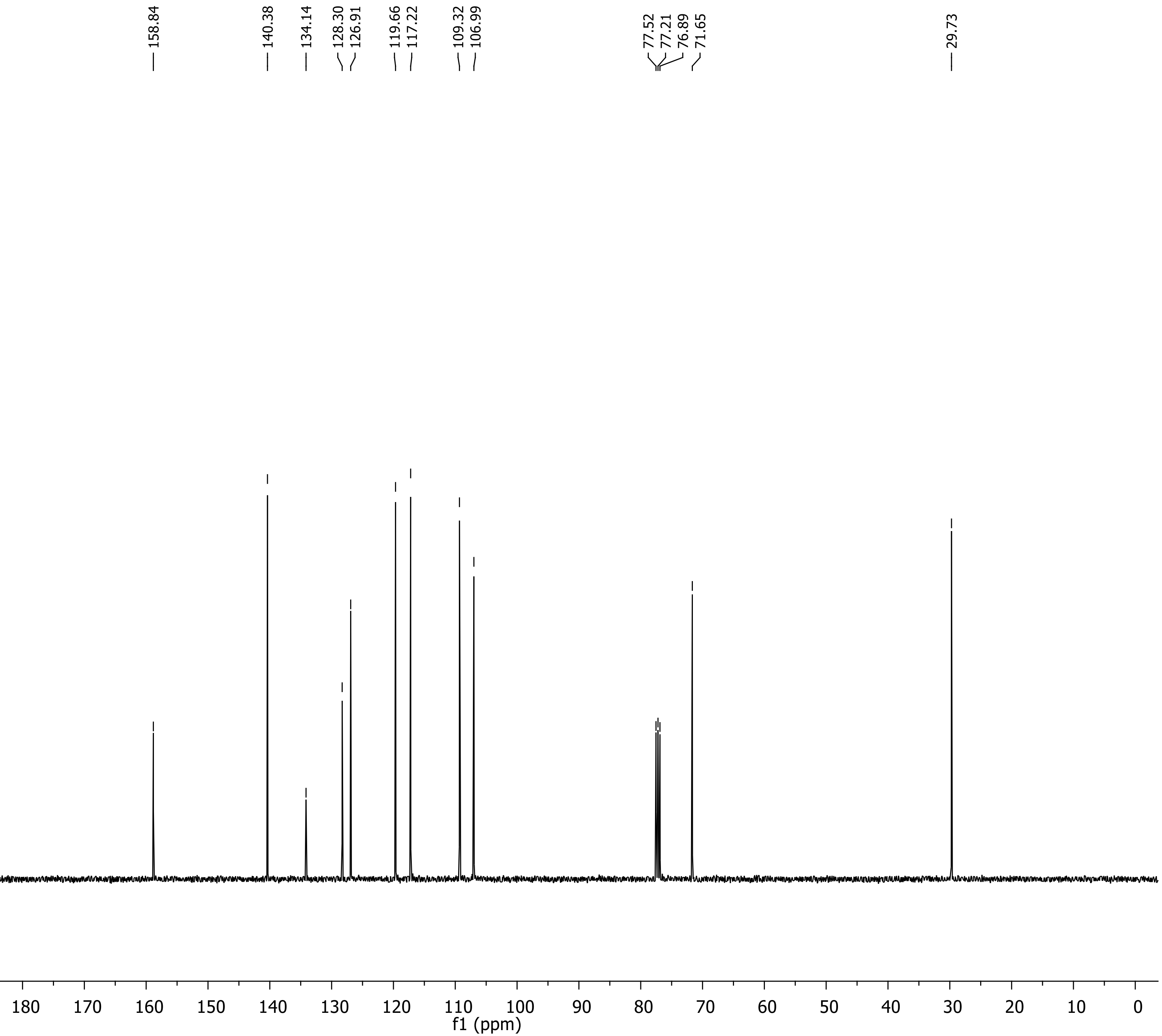

158.84
140.38
134.14
128.30
126.91
119.66
117.22
109.32
106.99
77.52
77.21
76.89
71.65
29.73
210 200 190 180 170 160 150 140 130 120 110 100 90 80 70 60 50 40 30 20 10 0
f1 (ppm)

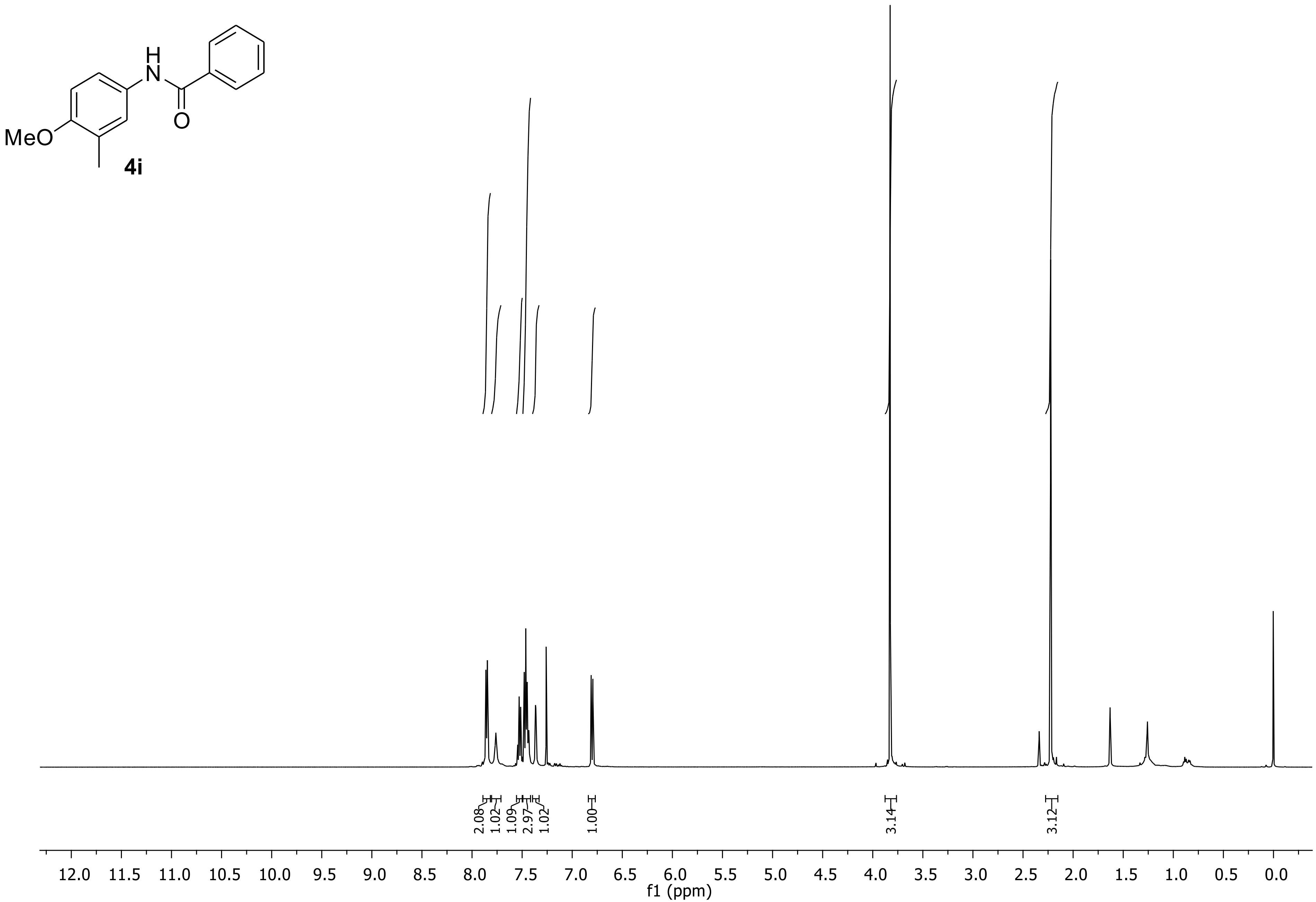
H
N
O
MeO
4i
2.08
1.02
1.09
2.97
1.02
1.00
3.14
3.12
12.0 11.5 11.0 10.5 10.0 9.5 9.0 8.5 8.0 7.5 7.0 6.5 6.0 5.5 5.0 4.5 4.0 3.5 3.0 2.5 2.0 1.5 1.0 0.5 0.0
f1 (ppm)

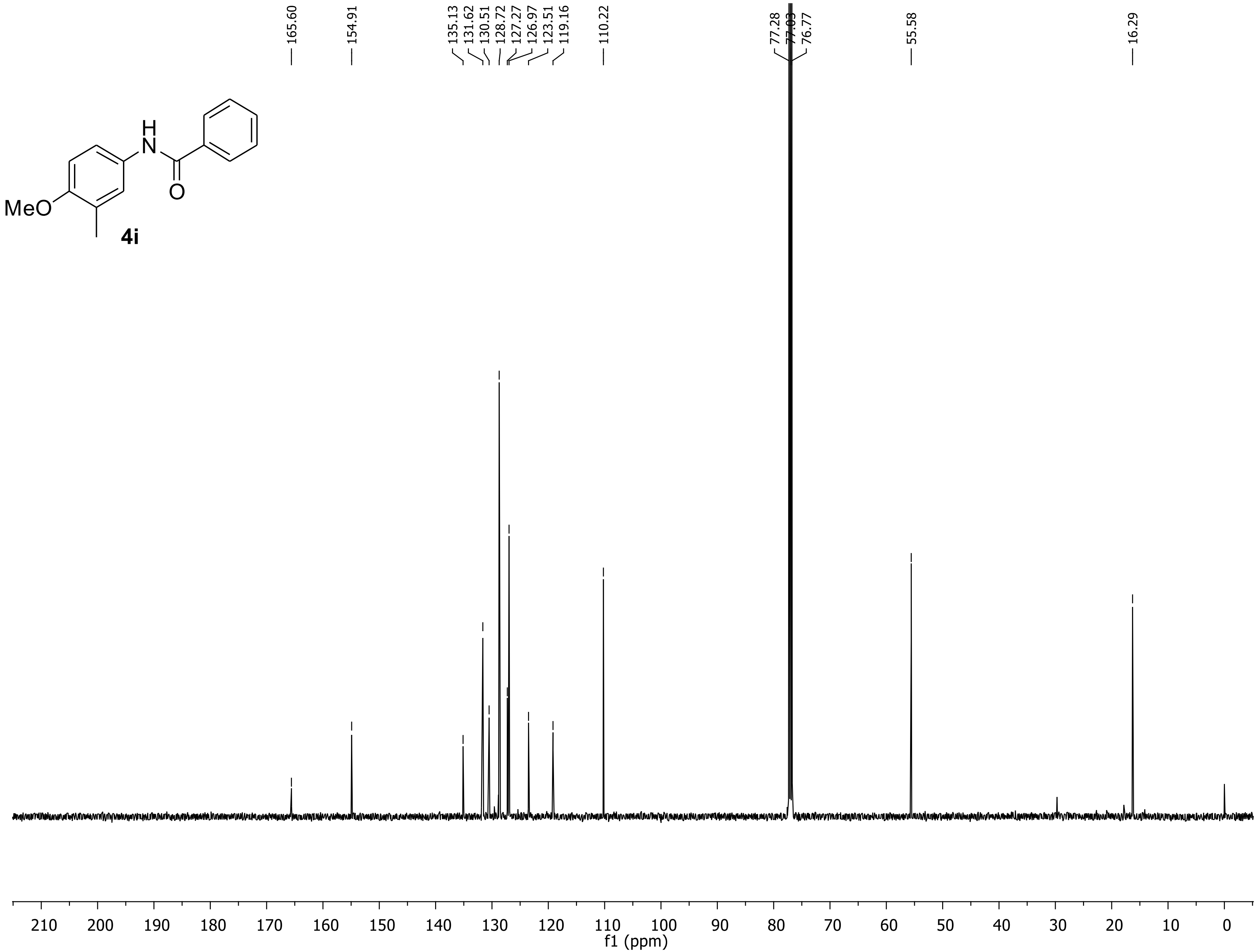
165.60
154.91
135.13
131.62
130.51
128.72
127.27
126.97
123.51
119.16
110.22
77.28
77.03
76.77
55.58
16.29
MeO
H
N
O
4i
210 200 190 180 170 160 150 140 130 120 110 100 90 80 70 60 50 40 30 20 10 0
f1 (ppm)

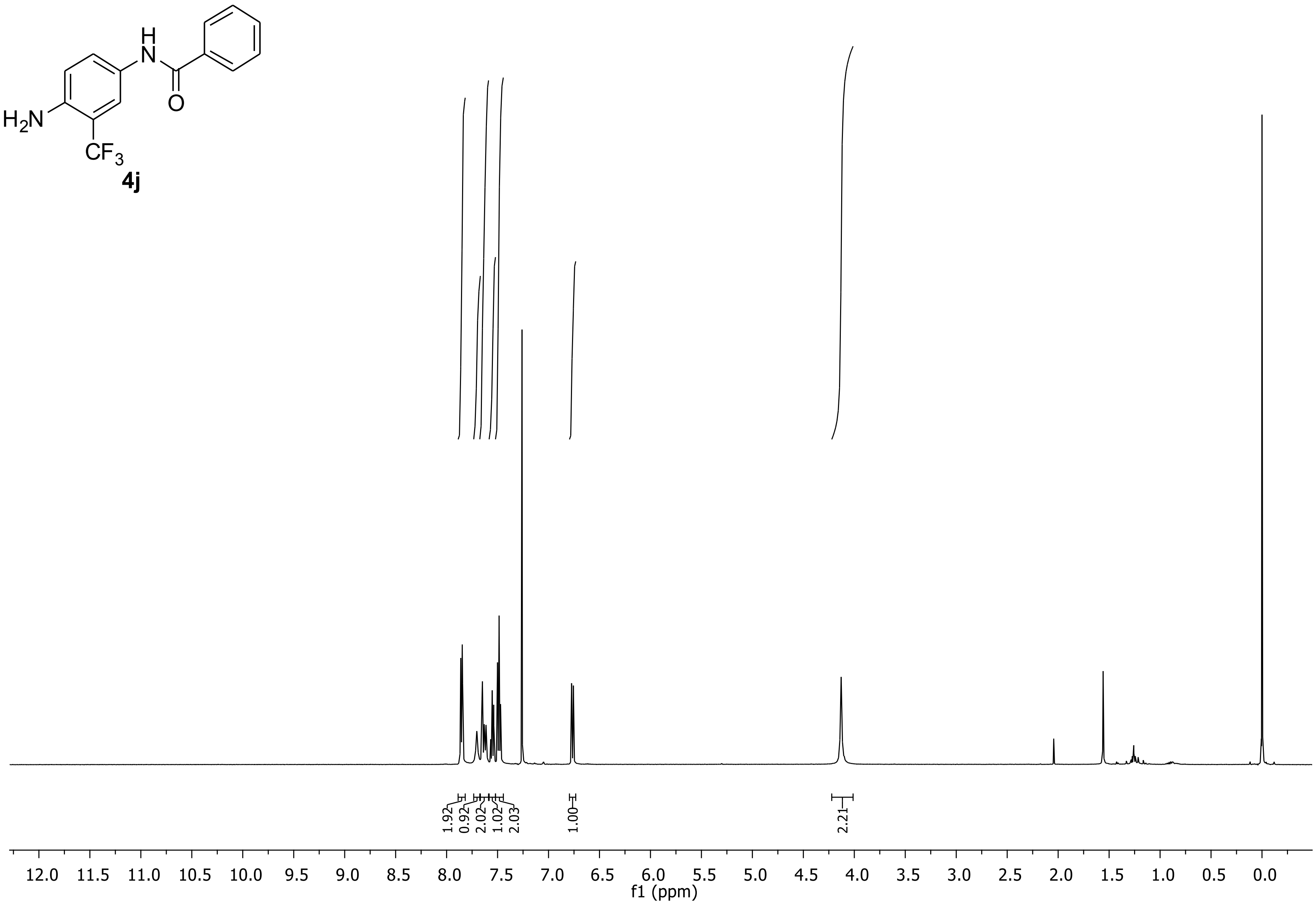
H
N
O
H2N
CF3
1.92
0.92
2.02
1.02
2.03
1.00
2.21
12.0
11.5
11.0
10.5
10.0
9.5
9.0
8.5
8.0
7.5
7.0
6.5
6.0
5.5
5.0
4.5
4.0
3.5
3.0
2.5
2.0
1.5
1.0
0.5
0.0
f1 (ppm)

4j

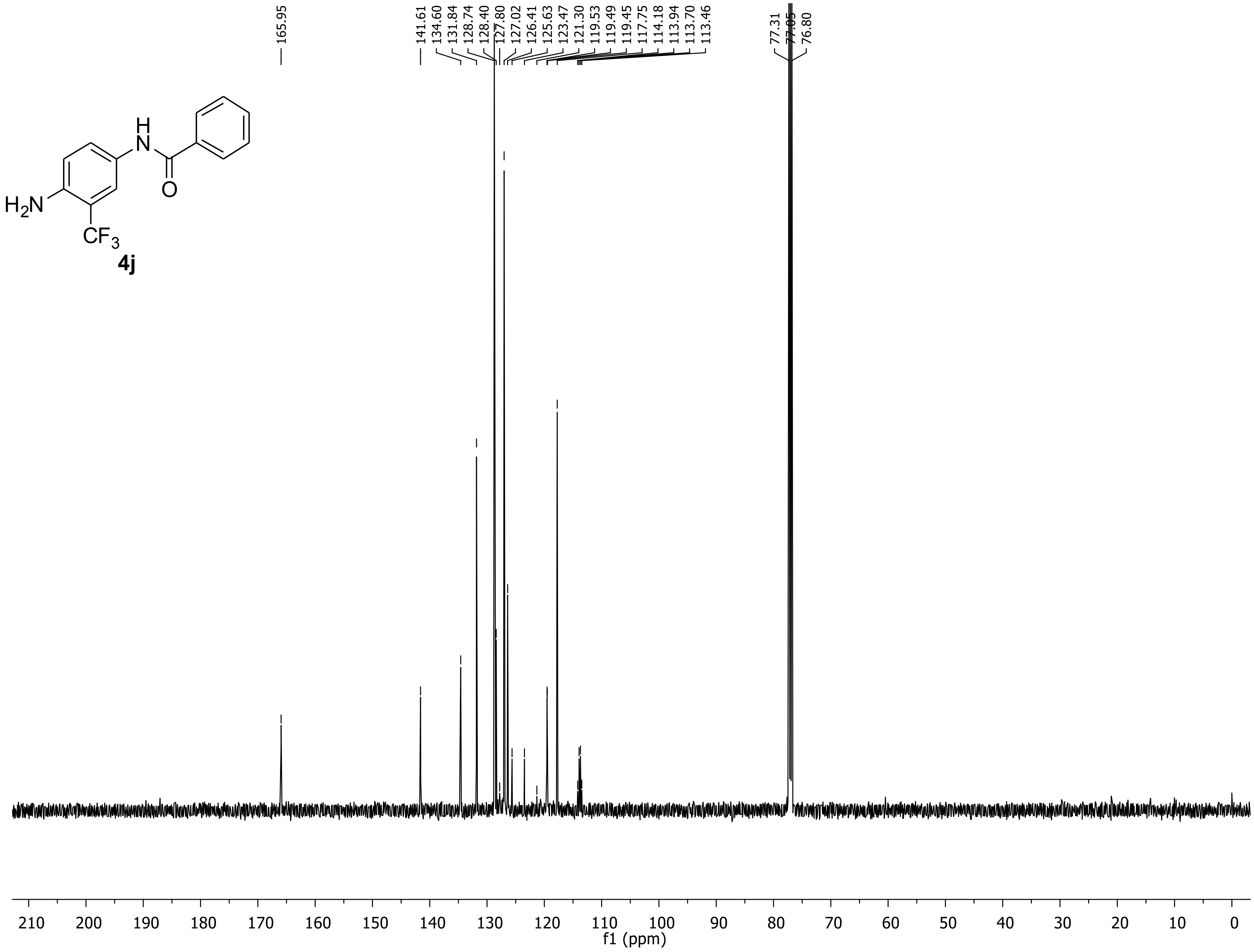
165.95
141.61
134.60
131.84
128.74
128.40
127.80
127.02
126.41
125.63
123.47
121.30
119.53
119.49
119.45
117.75
114.18
113.94
113.70
113.46
77.31
77.05
76.80
H
N
O
H_2N
CF_3
4j
210
200
190
180
170
160
150
140
130
120
110
100
90
80
70
60
50
40
30
20
10
0
f1 (ppm)

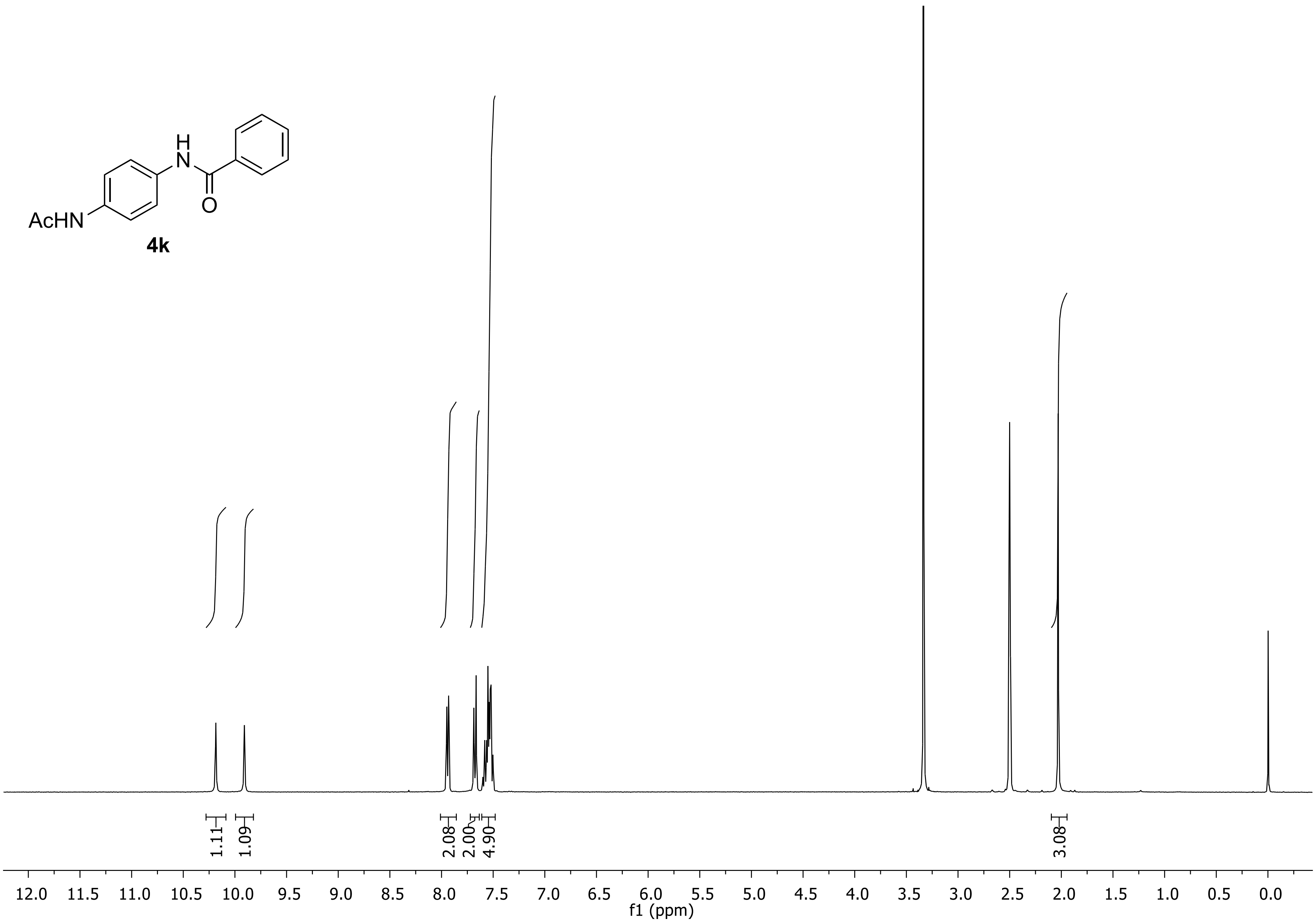
AcHN
H
N
O
4k
1.11
1.09
2.08
2.00
4.90
3.08
12.0 11.5 11.0 10.5 10.0 9.5 9.0 8.5 8.0 7.5 7.0 6.5 6.0 5.5 5.0 4.5 4.0 3.5 3.0 2.5 2.0 1.5 1.0 0.5 0.0
f1 (ppm)

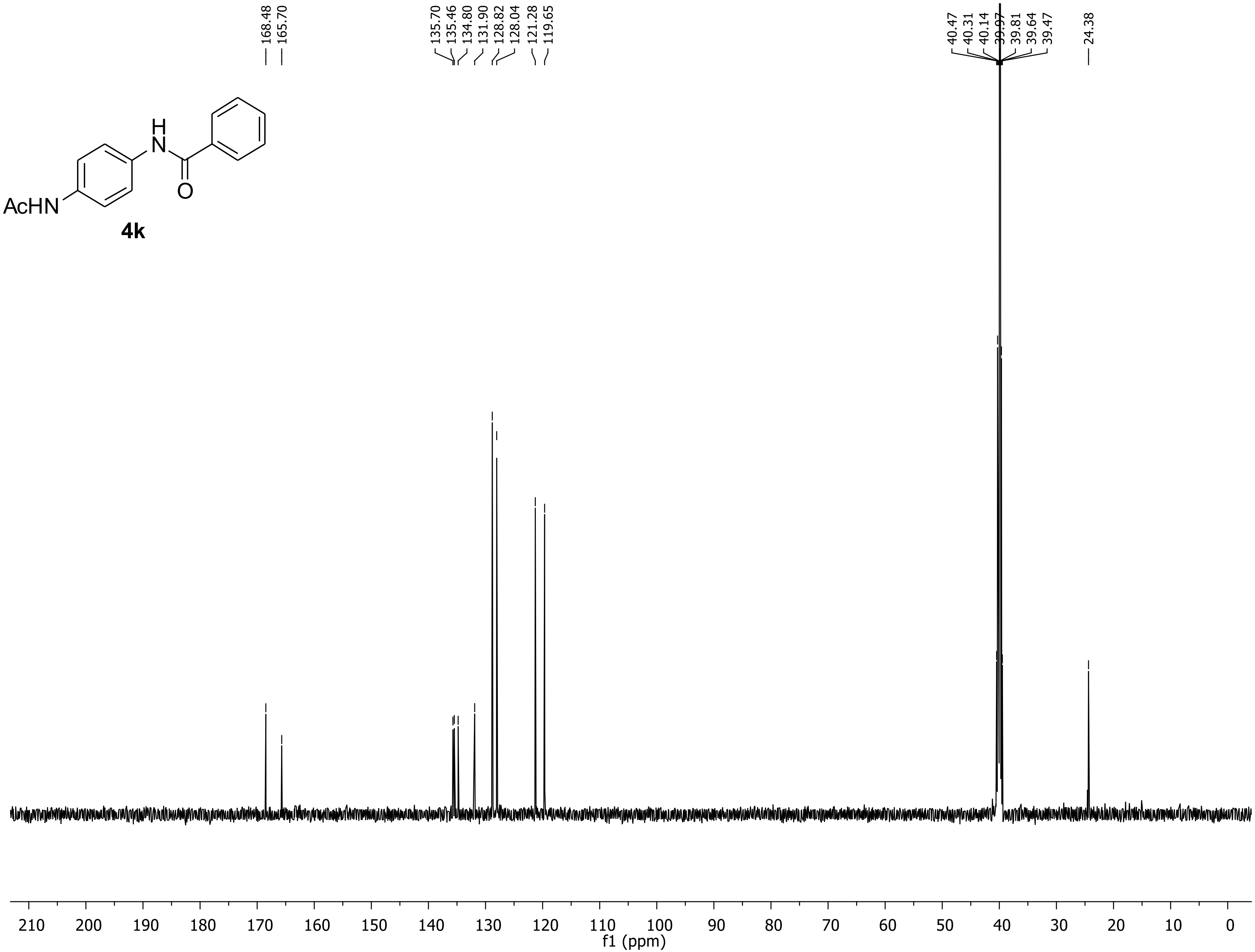
AcHN
H
N
O
168.48
165.70
135.70
135.46
134.80
131.90
128.82
128.04
121.28
119.65
40.47
40.31
40.14
39.97
39.81
39.64
39.47
24.38
210 200 190 180 170 160 150 140 130 120 110 100 90 80 70 60 50 40 30 20 10 0
f1 (ppm)

4k

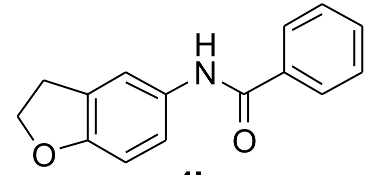
H
N
O
O

4l

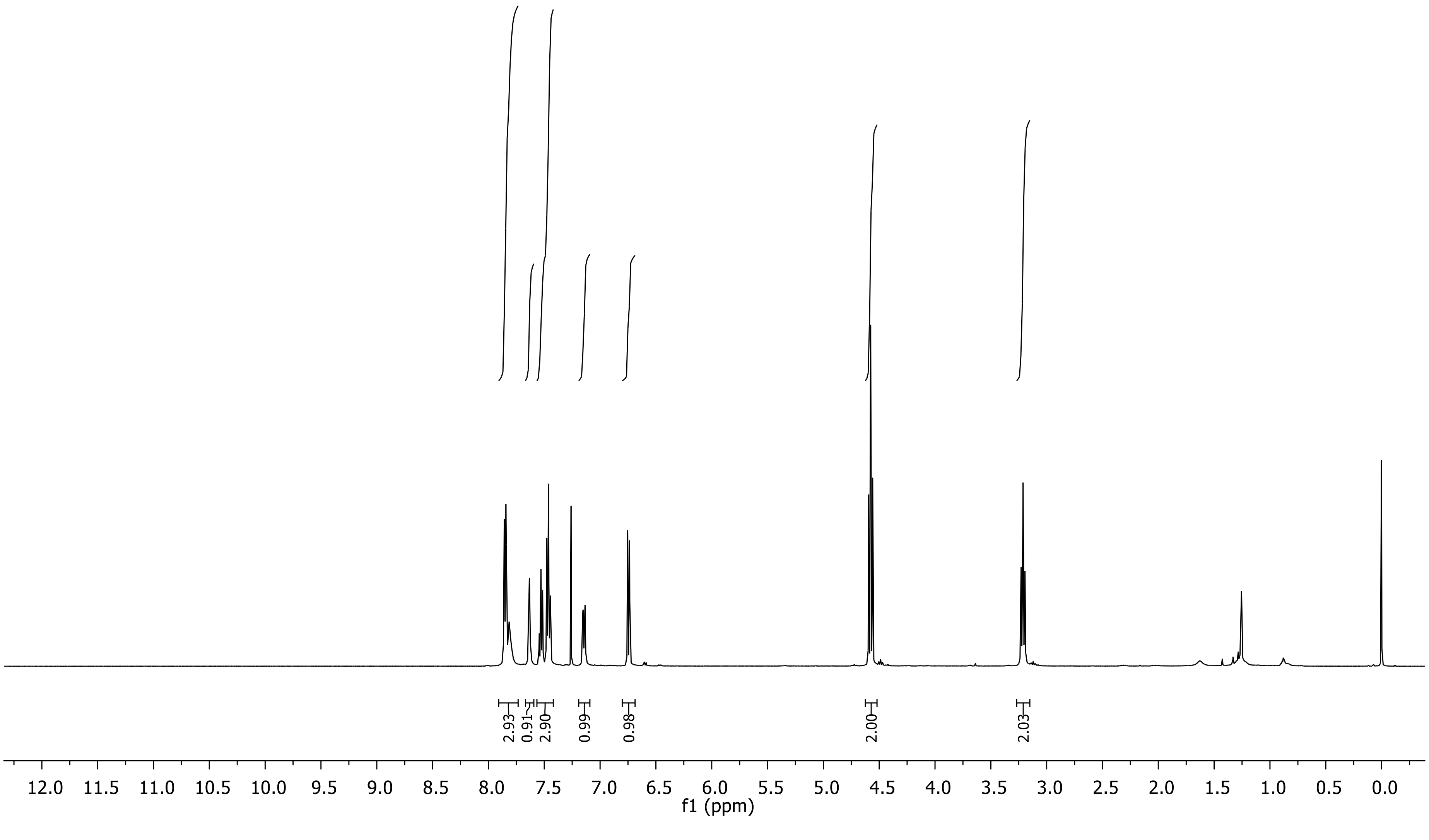
2.93
0.91
2.90
0.99
0.98
2.00
2.03
12.0 11.5 11.0 10.5 10.0 9.5 9.0 8.5 8.0 7.5 7.0 6.5 6.0 5.5 5.0 4.5 4.0 3.5 3.0 2.5 2.0 1.5 1.0 0.5 0.0
f1 (ppm)

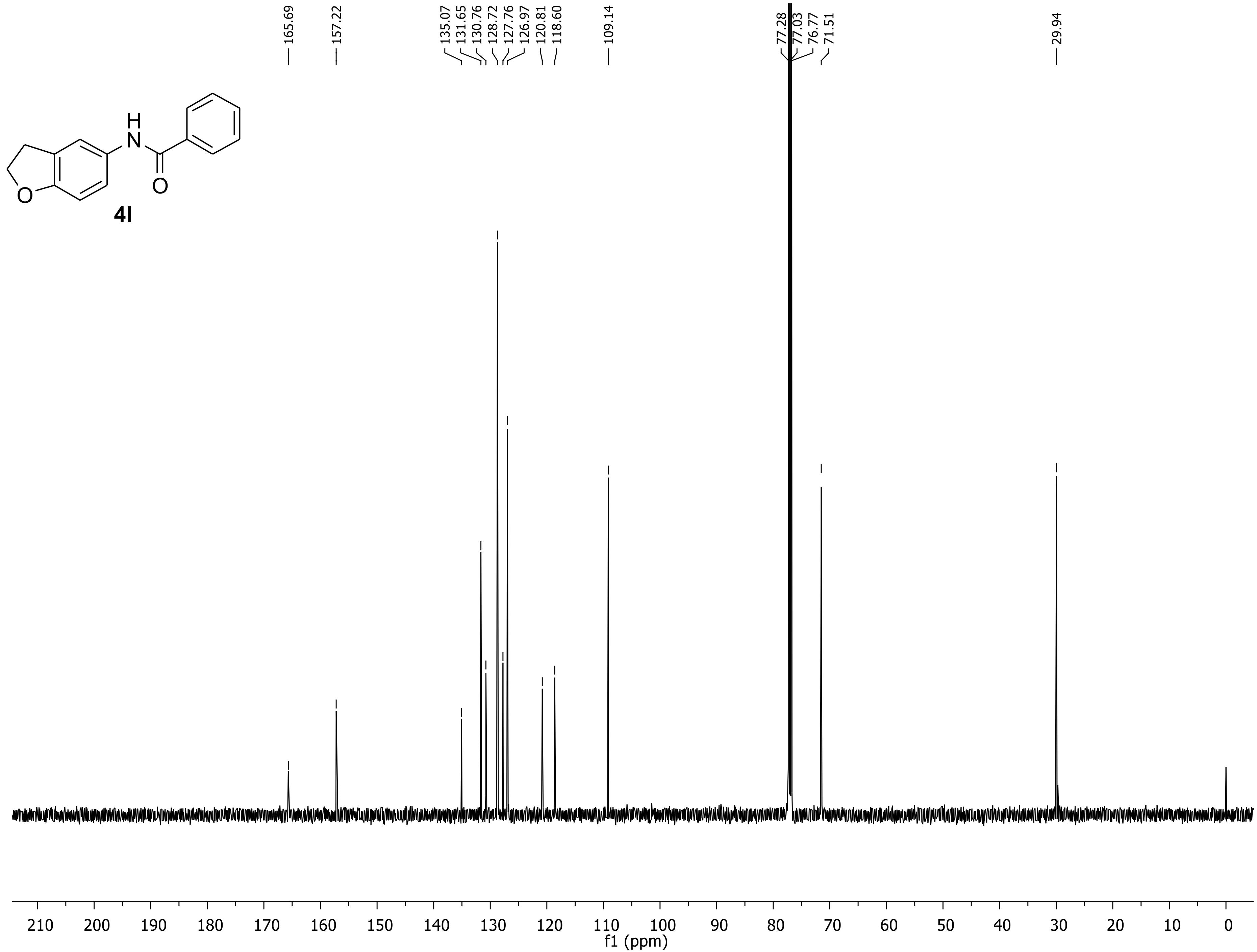

165.69
157.22
135.07
131.65
130.76
128.72
127.76
126.97
120.81
118.60
109.14
77.28
77.03
76.77
71.51
29.94
H
N
O
O
210 200 190 180 170 160 150 140 130 120 110 100 90 80 70 60 50 40 30 20 10 0
f1 (ppm)

4l

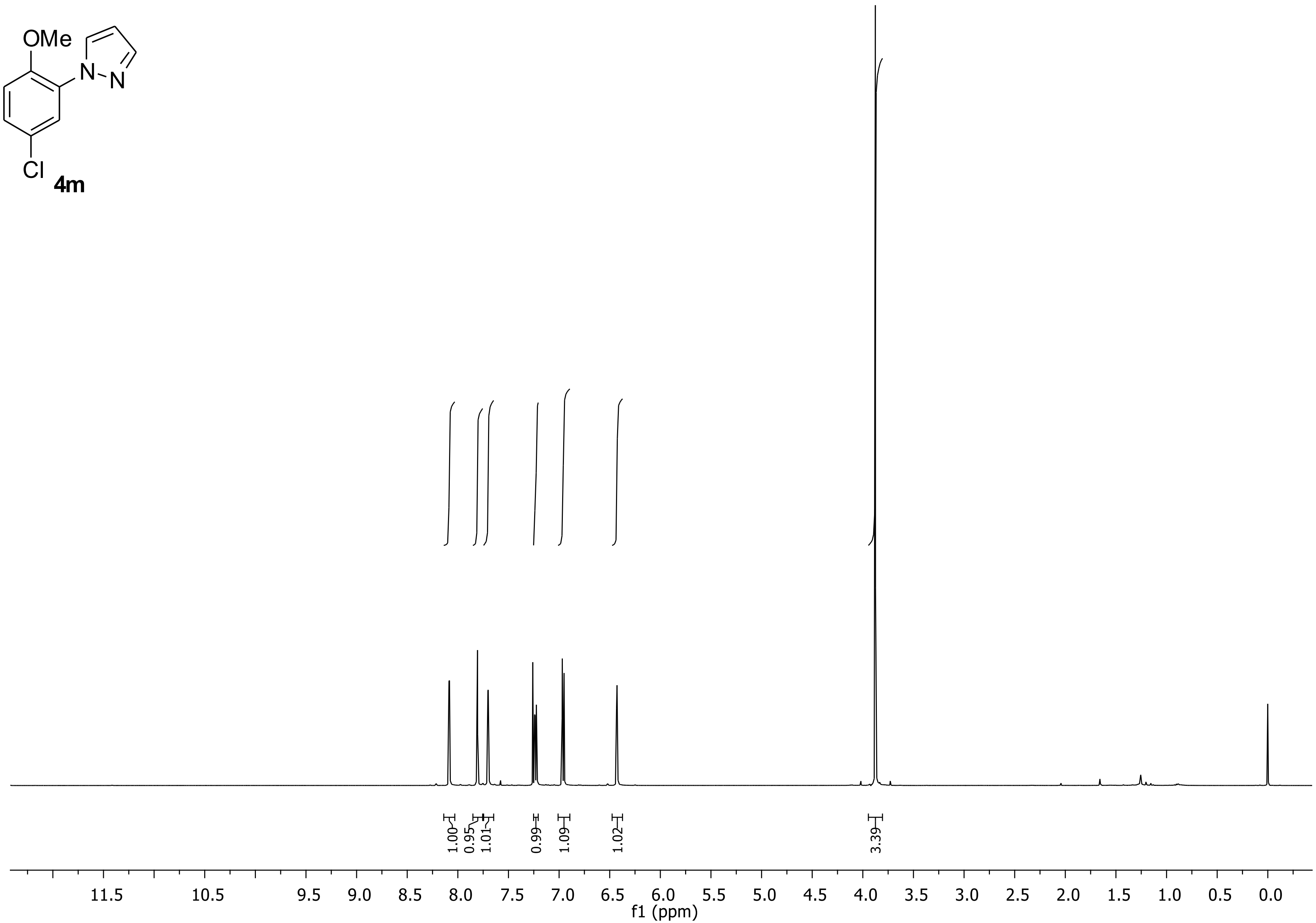

OMe
N
N
Cl
4m
1.00
0.95
1.01
0.99
1.09
1.02
3.39
11.5
10.5
9.5
9.0
8.5
8.0
7.5
7.0
6.5
6.0
5.5
5.0
4.5
4.0
3.5
3.0
2.5
2.0
1.5
1.0
0.5
0.0
f1 (ppm)

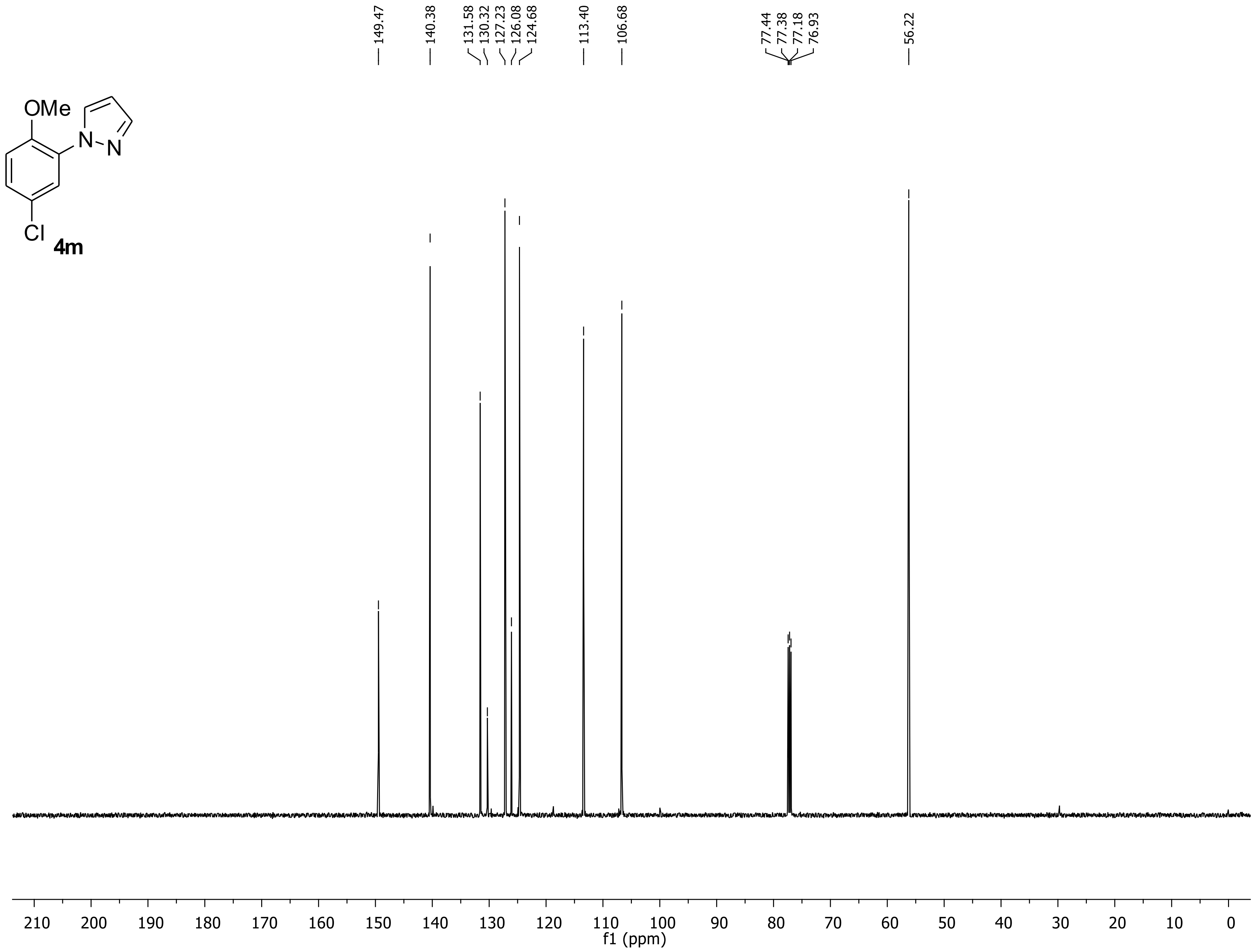
149.47
140.38
131.58
130.32
127.23
126.08
124.68
113.40
106.68
77.44
77.38
77.18
76.93
56.22
OMe
N
N
Cl
4m
210
200
190
180
170
160
150
140
130
120
110
100
90
80
70
60
50
40
30
20
10
0
f1 (ppm)
